# Green light powered molecular state motor enabling eight-shaped unidirectional rotation

**DOI:** 10.1038/s41467-019-12463-4

**Published:** 2019-10-01

**Authors:** Aaron Gerwien, Peter Mayer, Henry Dube

**Affiliations:** Department of Chemistry and Center for Integrated Protein Science CIPSM, Ludwig Maximilians-Universität München, Butenandtstrasse 5-13, 81377 München, Germany

**Keywords:** Organic chemistry, Molecular machines and motors

## Abstract

Molecular motors convert external energy into directional motions at the nano-scales. To date unidirectional circular rotations and linear motions have been realized but more complex directional trajectories remain unexplored on the molecular level. In this work we present a molecular motor powered by green light allowing to produce an eight-shaped geometry change during its unidirectional rotation around the central molecular axis. Motor motion proceeds in four different steps, which alternate between light powered double bond isomerizations and thermal hula-twist isomerizations. The result is a fixed sequence of populating four different isomers in a fully unidirectional trajectory possessing one crossing point. This motor system opens up unexplored avenues for the construction and mechanisms of molecular machines and will therefore not only significantly expand the toolbox of responsive molecular devices but also enable very different applications in the field of miniaturized technology than currently possible.

## Abstract (web summary)

Molecular motors have predominantly been limited to circular or linear directional motions. Here, the authors design a molecular motor that moves unidirectionally along a more complex figure-eight trajectory by a distinct four-step mechanism that alternates between photochemical and thermal reaction steps.

## Introduction

Synthetic molecular motors are miniaturized versions of macroscopic motors and convert external energy input into directional motions at the molecular level. Since the first examples have been developed by the groups of Feringa^1^ and Leigh^2^ in the late 1990s and early 2000s, a growing number of different systems have been established. These include chemically driven motors as well as light-driven ones^3–16^. We have contributed to the field with visible light-driven molecular motors^17–21^, which are based on the hemithioindigo (HTI) chromophore^22–32^. Most recently we have introduced a light-only powered molecular motor enabling stepwise directional motions without thermal ratcheting in the ground state^33^. To this end, the hula-twist (HT) photoreaction^34–38^, as recently experimentally evidenced by our group^38^, was employed in combination with simple double-bond isomerization (DBI) and single bond rotation (SBR) photoreactions. The HT motion was originally proposed as volume conserving photoreaction for the isomerization of retinal inside the opsin protein framework and proceeding as fully concerted process wherein a double bond and adjacent single bond rotate at the same time after photoexcitation^34–38^. According to Saltiel the HT photoreaction proceeds stepwise with the double-bond photoisomerizing first in the excited state and the single bond rotating afterwards in a hot ground-state reaction^37^. The term “double-bond isomerization (DBI)” refers here to the sole *E*–*Z* isomerization of the central double bond. This reaction is also called one-bond-flip (OBF) or one-bond-twist (OBT) in the literature^35,37^.

Despite this great progress in the development of molecular motors, the types of directional geometry changes available at present are almost exclusively simple circular rotations or linear locomotions. More complex directional molecular motions have only been realized in a combinatorial multi-switch fashion resulting in flexible and thus less defined geometrical changes in each motion step^39,40^. Herein, we present a simple molecular setup **1** that allows realization of a very precise and repetitive eight-shaped geometry change during unidirectional rotation of one molecular fragment around the other within a single switch architecture. To this end, an unusual type of thermal HT motion is employed alternating with photoinduced DBI. The term “thermal hula-twist (HT)” motion refers here to a 180° rotation of both the double bond and the adjacent single bond in the ground state leading to concomitant *E*–*Z* isomerization, as well as atropisomerization without observable intermediates.

## Results

### Ground-state properties of motor 1

**Fig. 1:**
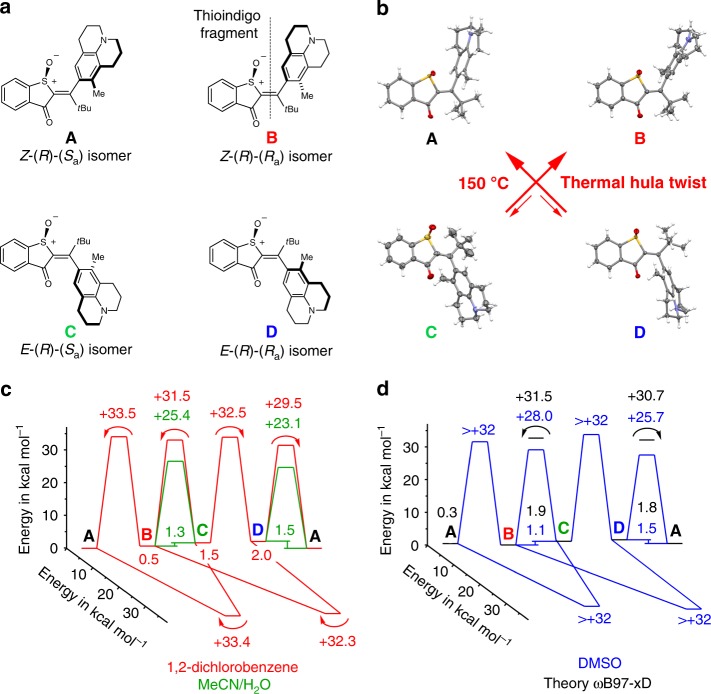
Structures of isomers **A** to **D** of motor **1** and ground-state properties. **a** Schematic representation of the molecular structures of **A** to **D**. **b** Structures of isomers **A** to **D** in the crystalline state and thermal interconversion in solution. **c** Ground-state energy profile of **1** experimentally determined in 12DCB-*d*_4_ (red) and in acetonitrile-*d*_3_/D_2_O (8/2, green). **d** Ground-state energy profile of **1** calculated at the ω-B97XD/6-311 G(d,p) PCM(DMSO) level of theory (black) and experimentally determined in DMSO-*d*_*6*_ (blue)

Motor **1** is derived from the parent HTI chromophore, and is equipped with a permanent sulfoxide stereocenter. In addition, a chiral axis is established via introduction of a nonsymmetric julolidine unit, the atropisomerization of which is slowed down by increased sterical hindrance in the molecule. Therefore, all four different diastereomeric states of **1 **— denoted **A** to **D** as shown in Fig. 1a — can be isolated, characterized, and their photoreactions as well as thermal reactions can be scrutinized individually. Synthesis of **1** follows an established protocol for the generation of sterically hindered, fourfold double-bond substituted HTI photoswitches^30^. Isolation of all individual racemic isomers **A** to **D** was achieved using HPLC separation, and their structures in the crystalline state were determined (Fig. 1b) to enable assignment of the corresponding solution spectra (Supplementary Figs. 1–4).

When heating solutions of pure isomers **A**, **B**, **C**, and **D** in different solvents, their thermal interconversions could be monitored directly. Interestingly, these isomers do not randomly interconvert at elevated temperatures but undergo a HT-like combined 180° rotation of both the central double bond and the adjacent single bond, which establishes the chiral axis. This behavior is unknown to the best of our knowledge, and also distinctively different to related HTI molecules reported earlier, showing first only SBR at elevated temperatures and after further temperature increase interconversion between all isomers^33,38^. In this case, isomer **D** thermally interconverts predominantly to **A** (e.g., in 93% at 27 °C in acetonitrile-*d*_3_/D_2_O (8/2) solution) in the thermal equilibrium. Likewise, **C** predominantly converts to **B** (e.g., in 87% at 60 °C in acetonitrile-*d*_3_/D_2_O (8/2) solution) in the thermal equilibrium. Again, this behavior is different to related oxidized HTI systems, where thermal equilibria with substantial fractions of different isomers being present are established^17,33,38^. From first-order kinetic analyses of thermal isomer interconversions and observed thermal equilibria, the ground-state energy profiles of **1** could experimentally be quantified in acetonitrile-*d*_3_/D_2_O (8/2), 1,2-dichlorobenzene-*d*_4_ (12DCB-*d*_4_), and DMSO-*d*_6_ solutions (Fig. 1c, d). In 12DCB-*d*_4_, it could be established that isomer **A** is the global minimum structure. In more apolar solvents such as cyclohexane or 12DCB-*d*_4_ energy barriers for thermal HT-bond rotations are considerably increased (by about 6 kcal/mol in 12DCB-*d*_4_) and more similar to the energy barriers for the simpler one-bond rotations. As a result higher temperatures are required for isomer interconversion and a mixture of mainly **A** and **B** isomers are obtained in thermal equilibrium (Supplementary Figs. 5–14 and Supplementary Tables 1–3).

The theoretical description on the ω-B97XD/6-311G(d,p) level of theory (Fig. 1d; Supplementary Fig. 26, Supplementary Tables 5–9) was found to be in good agreement with these experiments. However, the calculated energy barriers are in general higher than in the experiments. The calculations predict four possible transition states for the thermal HT reaction, showing only one imaginary vibrational mode. In all of them, the double bond is twisted by 90° and is now orthogonal to the thioindigo part of the molecule. At the same time, the adjacent single bond is also twisted by 90° and receives a large double-bond character. Given the similar degree of rotation of both adjacent bonds in the transition states, a fully concerted thermal HT-like rotation is predicted from the theoretical description. From the four possibilities, two transition states with the methyl group facing away from the tertiary butyl group are energetically significantly favored. As the experiments established two distinct and non-crossing thermal HT isomerizations (**C** to **B** with the larger energy barrier and **D** to **A** with the lower energy barrier), the two theoretically found transition states could be assigned to a particular process (Supplementary Fig. 26). In the transition state reached from isomer **C**, the julolidine rotated with its methyl group below the thioindigo fragment residing on the opposite face of the sulfoxide oxygen. This motion is then continued by further 90° rotation around the single and double bond leading to isomer **B**. The thermal HT reaction of isomer **D** leads to a transition state with the julolidine rotated with its methyl group above the thioindigo part residing on the same face of the sulfoxide oxygen. Again, this motion is completed by further 90° rotation around the former single and double bonds leading to isomer **A** (see also Supplementary Movies 1–4).

### Photoreactions of motor 1

**Fig. 2:**
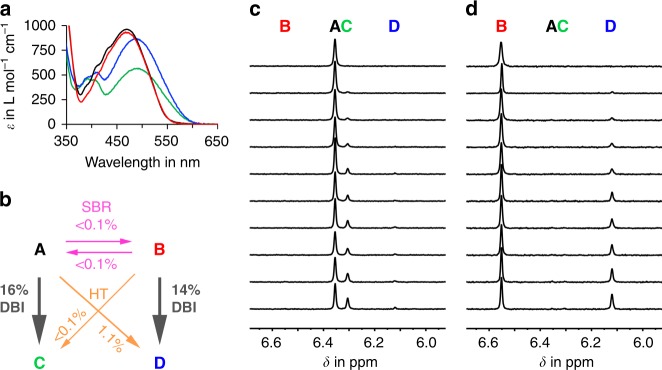
Photoreactions of HTI **1** during 520 nm irradiation. **a** Molar absorption coefficients in cyclohexane solution. **b** Experimental quantum yields determined for the photoreactions of isomers **A** and **B** in cyclohexane-*d*_12_ solution at 27 °C under 520 nm irradiation. **c** Beginning of the photoreaction of isomer **A** in cyclohexane-*d*_12_ at 27 °C. **d** Beginning of the photoreaction of isomer **B** in cyclohexane-*d*_12_ at 27 °C

**Fig. 3:**
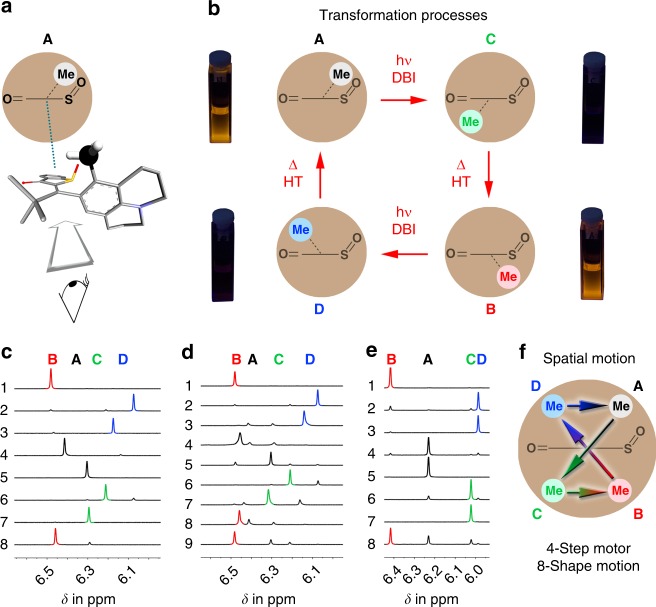
Molecular motor properties of **1**. **a** Schematic representation of the geometry of **A**. **b** Four-step process of the motor motion and associated fluorescence changes. **c** Individual steps of motor operation followed by ^1^H NMR spectroscopy, (1) **B** in cyclohexane-*d*_12_, (2) after 520 nm irradiation, (3) **D** in acetonitrile-*d*_3_/D_2_O (8/2), (4) after heating to 27 °C, (5) **A** in cyclohexane-*d*_12_, (6) after 520 nm irradiation, (7) **C** in acetonitrile-*d*_3_/D_2_O (8/2), (8) after heating to 60 °C. **d** One full cycle of motor operation followed by ^1^H NMR spectroscopy, (1) **B** in cyclohexane-*d*_12_, (2) after 520 nm irradiation, (3) after solvent change to acetonitrile-*d*_3_/D_2_O (8/2), (4) after heating to 60 °C, (5) after solvent change to cyclohexane-*d*_12_, (6) after irradiation with 520 nm, (7) after solvent change to acetonitrile-*d*_3_/D_2_O (8/2), (8) after heating to 60 °C, (9) after solvent change to cyclohexane-*d*_12_. **e** Individual steps of autonomous motor operation followed by ^1^H NMR spectroscopy in 12DCB-*d*_4_, (1) pure **B**, (2) after 520 nm irradiation, (3) pure **D**, (4) after heating to 130 °C, (5) pure **A**, (6) after 520 nm irradiation, (7) pure **C**, (8) after heating to 130 °C. **f** Positional changes of the methyl group with respect to the static thioindigo fragment during one full cycle of directional motion

After establishing the thermal reactions of **1**, its photoreactions were analyzed separately for each isomer **A**, **B**, **C**, and **D** at 25 °C in cyclohexane-*d*_12_ (Fig. 2a–d) and 12DCB solutions. In more polar solvents, the photoreactions are strongly reduced rendering them impractical. Three different photoreactions are possible for each isomer: a simple DBI, a SBR around the chiral axis, and a combined HT photoreaction where both adjacent bonds are rotated. Since thermal isomer interconversion is halted completely at 25 °C, the primary photoproducts are directly observed in the irradiation experiments and are not obscured by fast thermal follow-up isomerizations. Quantum yields for individual transformations during 520 nm irradiation were measured in cyclohexane-*d*_12_ and 12DCB (see Fig. 2b; Supplementary Figs. 18–21, Supplementary Table 4). Again, stark differences to previously reported related HTIs, which do in fact undergo all three photoreactions for each isomer^33,40^, are observed. In this case, DBI is performed with very high selectivity leading to efficient photoconversion of **A** to **C** as well as of **B** to **D** while all other photoreactions are at least one order of magnitude less efficient. The corresponding backward photoreactions of **C** and **D** are even less efficient as neither **A** or **B** photoproducts were observed when irradiating pure **C** or **D** and can thus be regarded as non-interfering. Consequently, strong photoenrichment of **C** and **D** is possible in the photostationary state (pss, see Fig. 3; Supplementary Figs. 18–21, Supplementary Table 4).

## Discussion

Taking both thermal interconversions and photoreactions into account, a repetitive unidirectional motion of HTI **1** can be established in four distinct steps (Fig. 3a, b). Every individual step was first conducted separately starting from the respective pure isomer to determine its completeness (see Fig. 3c and Supplementary Fig. 23). Afterward, the whole sequence was conducted in a row starting from pure isomer **B** (see Fig. 3d and Supplementary Fig. 22). The first step consists of irradiation of isomer **B** in cyclohexane-*d*_12_ with 520 nm light resulting in DBI and enrichment of isomer **D** to 86% in the pss. Second a solvent change to acetonitrile-*d*_3_/D_2_O (8/2) solution and subsequent heating to 60 °C for 3 min leads to population of isomer **A** in 94% via thermal HT. Another solvent change back to cyclohexane-*d*_12_ and irradiation with 520 nm results in DBI of **A** to yield 86% of **C**. A final solvent change to acetonitrile-*d*_3_/D_2_O (8/2) solution and subsequent heating to 60 °C for 120 min results again in thermal HT to populate the starting isomer **B** in 88%. The overall process therefore interconverts all four isomers of **1** into each other in a fixed sequence that does not contain “backwards motions”. When this sequence is done within one experiment in a row enrichment of individual isomers in each step is slightly less complete as the minor isomers produced add up (see Supplementary Fig. 22). In this case, 63% of the molecules performed a full rotation while the other molecules did not complete the cycle as every step proceeds only with conversions of about 90% on average. Repetitive solvent changes were employed to maximize the efficiencies of each individual step in the sequence. However, using 12DCB as solvent allows to continuously power the directional motion at high temperatures of 130 °C without the need of solvent changes (Fig. 3e). 12DCB represents a good compromise between solvent polarity and high boiling point rendering photoisomerizations as well as thermal HT reactions selective and efficient enough to warrant full motor operation (Supplementary Figs. 24, 25).

To quantify unidirectionality, the selectivity of each thermal and photoreaction has to be determined. The selectivity for the thermal steps in acetonitrile-*d*_3_/D_2_O (8/2) solution can be considered as 100%, as the barriers for the other rotations are much higher and only one product isomer is formed. The photoreaction of **B** in cyclohexane-*d*_12_ occurs with 92% selectivity to the **D** isomer, while also 8% **C** is formed in the pss. The photoreaction of **A** proceeds with 89% selectivity leading to the **C** isomer, while also 11% **D** is formed. The total degree of unidirectionality is therefore 82% when changing solvents. In 12DCB, thermal steps become less selective and the degree of unidirectionality is lowered to 47%.

Next, we visualize the geometry changes occurring during this four-step process of the directional motion. HTI motor **1** establishes the fixed isomer sequence **ACBD** as shown in Fig. 3b. The clearest picture emerges when regarding the thioindigo fragment as static while the julolidine moiety represents the revolving unit. The most obvious changes are then seen for the *ortho*-methyl group of the julolidine moiety, which follows an eight-shaped spatial change during the full directional motion with respect to the static fragment (Fig. 3f). At present, it is not possible to elucidate the exact full trajectory of the directional motion as the DBI steps can either occur clockwise or counterclockwise in the photoisomerization steps. It seems, however, likely that one trajectory will be favored given the asymmetry of the molecule. For the thermal HT motions, our quantum chemical calculations clearly show that these combined rotations occur on opposite faces of the thioindigo fragment when starting from either **C** or **D** (see Supplementary Fig. 26). Only four possible trajectories remain in total, which are presented in Supplementary Movies 1–4. It is clear that all of them are in no case simple circular rotations but follow more complex directional pathways that unequivocally include one crossing point and therefore form a directional eight-shaped path. Furthermore, the remaining possibilities do not cancel each other out ensuring overall directionality if the motor is driven continuously and one-directional cycle **ACBD** is followed by another and so on. Currently, it is not possible to make a clear assertion whether one eight shape is preferred over the others.

An additional benefit of HTI motor **1** is the possibility of photonic readout for its operational state. Only isomers **A** and **B** show yellow fluorescence, while isomers **C** and **D** remain non-emissive in cyclohexane (Fig. 3b). Therefore, the stepwise motion can be followed via appearing and disappearing fluorescence offering interesting possibilities for i.a. online-monitoring of molecular motor operation (e.g., determination of the endpoint of the irradiation step) or photonic device building^41–47^.

In summary, we present herein a molecular motor allowing the realization of a well-defined repetitive eight-shaped geometry change during its directional motion. Motor **1** is based on the HTI chromophore endowed with a sulfoxide stereocenter and a sterically hindered chiral axis. The unidirectional motion is powered by green light and proceeds in four distinct steps alternating two DBI photoreactions with two thermal HT reactions. Isomer interconversion can be followed visually by associated fluorescence changes. In 12DCB solution, the directional motion can be driven continuously at high temperatures. With this type of molecular machine, more complex unidirectional trajectories than purely circular or linear motions can now be generated at the molecular level offering unique potential for future nanomachinery and controllable molecular behavior.

## Methods

### Synthesis

The synthesis of **1** from commercially available reagents took place over five linear steps (seven in total) with an overall yield of 10%. All synthetic procedures and spectroscopic characterizations of **A**-**1**, **B**-**1**, **C**-**1**, and **D**-**1** as well as of synthetic intermediates are given in the Supplementary Methods. Reagents and solvents were obtained from abcr, Acros, Fluka, Merck, Sigma-Aldrich, or TCI in the qualities puriss., p.a., or purum and used as received. Technical solvents were distilled before use for column chromatography and extraction on a rotary evaporator (Heidolph Hei VAP Value, vacuubrand CVC 3000). Reactions were monitored on Merck Silica 60 F254 TLC plates. Detection was done by irradiation with UV light (254 nm or 366 nm). Column chromatography was performed with silica gel 60 (Merck, particle size 0.063–0.200 mm) and distilled technical solvents. ^1^H NMR and ^13^C NMR spectra were measured on a Varian Mercury 200 VX, Varian 300, Inova 400, Varian 600 NMR, or Bruker Avance III HD 800 MHz spectrometer at 23 °C. Deuterated solvents were obtained from Cambridge Isotope Laboratories or Eurisotop and used without further purification. Residual solvent signals in the ^1^H and ^13^C NMR spectra were used as internal reference. For ^1^H NMR, CDCl_3_  = 7.26 ppm, CD_2_Cl_2_ = 5.32 ppm, benzene-*d*_6_ = 7.16 ppm, (CD_3_)_2_SO = 2.50 ppm, 1,2-dichlorobenzene-*d*_4_ = 6.93. For ^13^C NMR, CDCl_3_ = 77.16 ppm. The resonance multiplicity is indicated as s (singlet), d (doublet), t (triplet), q (quartet), and m (multiplet). The chemical shifts are given in parts per million (ppm) on the delta scale (*δ*). The coupling constant values (*J*) are given in hertz (Hz). Electron impact (EI) mass spectra were measured on a Finnigan MAT95Q or on a Finnigan MAT90 mass spectrometer. Electronspray ionization (ESI) mass spectra were measured on a Thermo Finnigan LTQ-FT. The most important signals are reported in m/z units with M as the molecular ion. Elemental analysis were performed in the micro analytical laboratory of the LMU department of chemistry on an Elementar Vario EL apparatus. Infrared spectra were recorded on a Perkin Elmer Spectrum BX-FT-IR instrument equipped with a Smith DuraSamplIR II ATR-device. Transmittance values are qualitatively described by wavenumber (cm^−1^) as very strong (vs), strong (s), medium (m) and weak (w). UV/Vis spectra were measured on a Varian Cary 5000 spectrophotometer. Fluorescence spectra were measured on a Varian Eclipse spectrophotometer. The spectra were recorded in a quartz cuvette (1 cm). Solvents for spectroscopy were obtained from VWR and Merck. Absorption wavelength (*λ*) are reported in nm, and the molar absorption coefficients (*ε*) in L mol^−1^ cm^−1^. Melting points (M.p.) were measured on a Stuart SMP10 melting point apparatus in open capillaries, and are not corrected.

### Thermal isomerizations

Amberized NMR tubes were charged with 1 mg to 2.5 mg of the respective isomer of **A**-**1**, **B**-**1**, **C**-**1**, and **D**-**1** and 0.7 mL of 1,2-dichlorobenzene-*d*_4_, DMSO-*d*_6_, or MeCN-*d*_4_/D_2_O: 8/2. Subsequently the NMR tubes were heated to the appropriate temperatures in a stirred oil bath for isomerization reactions to occur, and the kinetics were followed by integration of the corresponding signals in the ^1^H NMR measurements in defined time intervals. The equilibrium ratio of isomers was obtained after prolonged heating until the integration values did not change anymore. The obtained experimental data, required equations, and the resulting energy barriers are shown in Supplementary Methods, Supplementary Figs. 5–14, and Supplementary Tables 1–3.

### Quantum yield measurements

A stock solution of the respective isomer **A**-**1** and **B**-**1** in 12DCB or cyclohexane (10 mL, 0.87 mm–1.01 mm) was prepared. One milliliter of the stock solutions was transferred into a quartz cuvette (1 cm) and irradiated with a focused light beam of a 520 nm LED within the published instrumental setup from the group of E. Riedle^48^. The number of absorbed photons over time *n*(*hv*) was measured directly at the thermal photometer of the instrument. After defined time intervals, the solutions were transferred form the quartz cuvette into an amberized NMR tube, the solvent was removed in vacuo and replaced with CD_2_Cl_2_ or benzene-*d*_6_. The concentrations of photoproducts were obtained by integrations of the corresponding signals in the ^1^H NMR spectra. This procedure was repeated up to ten times with different irradiation times allowing to average over all measurements. The obtained experimental data, required equations, and the resulting quantum yields are shown in the Supplementary Methods, Supplementary Figs. 18–21, and Supplementary Table 4.

### Motor operation followed by NMR spectroscopy

With changing solvents: Isomer **B**-**1** (1 mg) was dissolved in cylcohexane-*d*_12_ and a ^1^H NMR spectrum was recorded (starting point, **B** isomer). Afterward, the NMR tube was irradiated with a 520 nm LED until the fluorescence vanished and again a ^1^H NMR spectrum was recorded (motor step 1, mainly **D** isomer was obtained). Cylcohexane-*d*_12_ was removed in vacuo, and replaced with acetonitrile-*d*_3_/D_2_O (8/2) and immediately a ^1^H NMR spectrum was recorded (motor step 1, mainly **D** isomer present). After heating to 60 °C for a few minutes, another ^1^H NMR spectrum was recorded (motor step 2, mainly **A** isomer was obtained). Acetonitrile-*d*_3_/D_2_O (8/2) was removed in vacuo and replaced with cylcohexane-*d*_12_ and a ^1^H NMR spectrum was recorded (motor step 2, mainly **A** isomer present). The NMR tube was irradiated with a 520 nm LED until the fluorescence vanished and a ^1^H NMR spectrum was recorded (motor step 3, mainly **C** isomer was obtained). Cylcohexane-*d*_12_ was removed in vacuo and replaced with acetonitrile-*d*_3_/D_2_O (8/2), and immediately a ^1^H NMR spectrum was recorded (motor step 3, mainly **C** isomer present). After heating to 60 °C for a few minutes, another ^1^H NMR spectrum was recorded (motor step 4, mainly **B** isomer was obtained, starting point was reached again). Without changing solvent for autonomous operation: Isomer **B**-**1** (1 mg) was dissolved in 12DCB and one drop of benzene-*d*_6_ for easier locking and shimming was added and a ^1^H NMR spectrum was recorded (starting point, **B** isomer). Afterward, the NMR tube was irradiated with a 520 nm LED, and again a ^1^H NMR spectrum was recorded (motor step 1, mainly **D** isomer was obtained). After heating to 150 °C, another ^1^H NMR spectrum was recorded (motor step 2, mainly **A** isomer was obtained). The NMR tube was irradiated again with a 520 nm LED and a ^1^H NMR spectrum was recorded (motor step 3, mainly **C** isomer was obtained). After heating to 150 °C, another ^1^H NMR spectrum was recorded (motor step 4, mainly **B** isomer was obtained, the starting point was reached again). The obtained ^1^H NMR spectra after all steps and the corresponding ratios of the isomers are shown in Supplementary Figs. 22–25.

## Supplementary information

Supplementary Information

Description of Additional Supplementary Files

Supplementary Movie 1

Supplementary Movie 2

Supplementary Movie 3

Supplementary Movie 4

## Data Availability

All data that support the findings of this study are available from the Supplementary Information or from the corresponding author H. D. upon reasonable request. The X-ray crystallographic coordinates for the structures **A**-**1** to **D**-**1** reported in this study have been deposited at the Cambridge Crystallographic Data Centre (CCDC), under CCDC numbers 1910546 (**A**-**1**), 1910547 (**B**-**1**), 1910548 (**C**-**1**), and 1910549 (**D**-**1**). These data can be obtained free of charge from the Cambridge Crystallographic Data Centre via www.ccdc.cam.ac.uk/data_request/cif.
