# Supplementary material for: Green light powered molecular state motor enabling eight-shaped unidirectional rotation
Source: Nat Commun. 2019 Oct 1;10:4449. doi: 10.1038/s41467-019-12463-4 (PMC6773862; doi:10.1038/s41467-019-12463-4)
Supplement: Supplementary file 1 — Supplementary Information [file 41467_2019_12463_MOESM1_ESM.pdf]

Supplementary Information

Green Light Powered Molecular State Motor Enabling Eight-Shaped Unidirectional Rotation

A. Gerwien et al.

Supplementary Methods

Synthesis

General experimental

Reagents and solvents were obtained from *abcr*, *Acros, Fluka, Merck, Sigma-Aldrich* or *TCI* in the qualities *puriss., p.a., or purum* and used as received. Technical solvents were distilled on a rotary evaporator (*Heidolph Hei-VAP Value*, *vacuubrand* CVC 3000) before use for column chromatography and extraction. Reactions were monitored on *Merck* Silica 60 F254 TLC plates. Detection was done by irradiation with UV light (254 nm or 366 nm).

Column chromatography was performed with silica gel 60 (*Merck*, particle size 0.063- 0.200 mm) and distilled technical solvents.

^{1}H NMR and ^{13}C NMR spectra were measured on a *Varian Mercury 200 VX, Varian 300, Inova 400, Varian 600 NMR*, or *Bruker Avance III HD 800 MHz* spectrometer. Chemical shifts (δ) are reported relative to tetramethylsilane as external standard. Residual solvent signals in the ^{1}H and ^{13}C NMR spectra were used as internal reference. Deuterated solvents were obtained from *Cambridge Isotope Laboratories* or *Eurisotop* and used without further purification. Deuterated solvents were obtained from Cambridge Isotope Laboratories or Eurisotop and used without further purification. For ^{1}H NMR: $CDCl_3$ = 7.26 ppm, CD_2Cl_2 = 5.32 ppm, benzene-d$_6$ = 7.16 ppm, $(CD_3)_2SO$ = 2.50 ppm, 1,2-dichlorobenzene-d_4 = 6.93. For ^{13}C NMR: $CDCl_3$= 77.16 ppm. Resonance multiplicity is indicated as *s* (singlet), *d* (doublet), *t* (triplet), *q* (quartet) and *m* (multiplet). The chemical shifts are given in parts per million (ppm) on the delta scale (δ). The coupling constant values (J) are given in hertz (Hz).

Electron Impact (EI) mass spectra were measured on a *Finnigan MAT95Q* or on a *Finnigan MAT90* mass spectrometer. **Electrospray ionisation (ESI) mass spectra were** measured on a *Thermo Finnigan LTQ-FT* mass spectrometer. The most important signals are reported in *m/z* units with *M* as the molecular ion.

Elemental analysis was performed in the micro analytical laboratory of the LMU department of chemistry on an *Elementar Vario EL* apparatus.

Infrared spectra were recorded on a *Perkin Elmer Spectrum BX-FT-IR* instrument equipped with a *Smith DuraSamplIR II ATR*-device. Transmittance values are qualitatively described by wavenumber (cm^{-1}) as very strong (vs), strong (s), medium (m) and weak (w).

UV/Vis and fluorescence spectra were measured on a *Varian Cary 5000* spectrophotometer. Fluorescence spectra were measured on a Varian Eclipse spectrophotometer. The spectra were recorded in a quartz cuvette (1 cm). Solvents for spectroscopy were obtained from *VWR* and *Merck*. Absorption wavelength (λ) are reported in nm and the molar absorption coefficients (ε) in L mol^{-1} cm^{-1}. Shoulders are declared as sh.

Melting points (M.p.) were measured on a *Stuart* SMP10 melting point apparatus in open capillaries and are not corrected.

Photoisomerization experiments. Continuous irradiations of the solutions were conducted in NMR tubes in different solvents (cyclohexane-d_{12}, 12DCB-d_4). Irradiations were conducted using a *Prizmatix UHP-T-520-DI LED* (520 nm) as light source.

HTI Synthesis

Precursors **2** and **3** were prepared following a previously reported protocol according to the following scheme.[1]

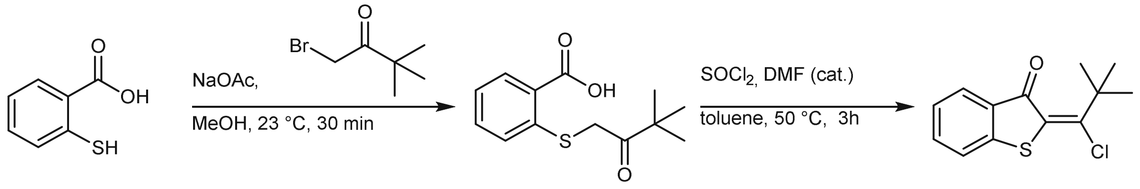

2 98% 3 92%

8-Methyl-2,3,6,7-tetrahydro-1*H*,5*H*-pyrido[3,2,1-*ij*]quinoline (4)

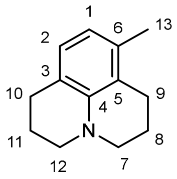

3-Toluidine (4.00 mL, 3.96 g, 37.0 mmol, 1 equiv.), 1-bromo-3-chloropropane (40.0 mL, 63.7 g, 404 mmol, 11 equiv.), sodium carbonate (15.0 g, 142 mmol, 3.8 equiv., oven dried at 300 °C *in vacuo* for 1 hour) and 4 Å molecular sieves (3.5 g, activated at 300 °C *in vacuo* for 1 hour) were added to a flame-dried 250 mL round bottom flask equipped with a reflux condenser. The reaction was heated stepwise: 1 h at 80 °C, 2 h at 100 °C and 12 h at 160 °C. Afterwards the reaction mixture was cooled to room temperature and 200 mL CH_2Cl_2 were added. The organic phase was washed successively with: HCl (2 M aqueous solution, 1 x 200 mL), H_2O (2 x 200 mL), NaOH (2 M aqueous solution, 1 x 200 mL), and again H_2O (2 x 200 mL). The organic phase was dried over sodium sulfate and after removing the solvent i*n vacuo* the crude product was purified by column chromatography (SiO_2, *i*Hex/EtOAc = 98/2 → 9/1). The title compound was isolated as colorless liquid (5.97 g, 31.9 mmol, 86%).

HR-MS (EI) for $C_{13}H_{17}N^{\cdot+}$, $[M]^{\cdot+}$, calcd. 187.1356, found 187.1352.
^{1}H NMR ($CDCl_3$, 400 MHz): δ (ppm) = 6.75 (d, J = 7.5 Hz, 1H, H-C(2)), 6.46 (d, J = 7.5 Hz, 1H, H-C(1)), 3.23 – 3.06 (m, 4H, H-C(7), H-C(12)), 2.78 (t, J = 6.6 Hz, 2H, H-C(10)), 2.67 (t, J = 6.7 Hz, 2H, H-C(9)), 2.17 (s, 3H, H-C(13)), 2.08 – 1.95 (m, 4H, H-C(8), H-C(11)).
^{13}C NMR ($CDCl_3$, 100 MHz): δ (ppm) = 143.4 (C(4)), 134.4 (C(6)), 126.6 (C(2)), 120.5 (C(5)), 119.8 (C(3)), 118.1 (C(1)), 50.6 (C(12)), 49.9 (C(7)), 27.8 (C(10)), 24.9 (C(9)), 22.5(C(11)), 22.4 (C(8)), 19.8 (C(13)).
IR: $\tilde{\upsilon}$/cm^{-1} = 2929 (s), 2836 (m), 2771 (m), 1672 (w), 1599 (m), 1578 (m), 1489 (vs), 1453 (s), 1439 (s), 1422 (m), 1392 (m), 1352 (m), 1327 (m), 1304 (vs), 1240 (w), 1208 (m), 1194 (s), 1186 (s), 1154 (m), 1120 (s), 1075 (w), 1047 (w), 1002 (w), 920 (m), 887 (w), 867 (w), 785 (vs), 737 (m), 718 (m).
R_f (SiO_2, *i*hex/EtOAc = 95/5) = 0.56.

9-Bromo-8-methyl-2,3,6,7-tetrahydro-1*H*,5*H*-pyrido[3,2,1-*ij*]quinoline (5)

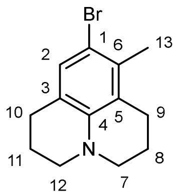

Compound **4** (3.58 g, 19.1 mmol, 1 equiv.) was dissolved in CH_2Cl_2 (10 mL) and cooled to -78 °C. Bromine (1.03 mL, 3.21 g, 20.1 mmol, 1.05 equiv.) was added over 10 min. After addition the reaction was immediately warmed to 22 °C and sodium thiosulfate solution (2 mL) and saturated sodium carbonate solution (10 mL) were added. The aqueous phase was extracted with CH_2Cl_2 (3 x 20 mL), and the combined organic phases were dried over Na_2SO_4. After removing the solvent i*n vacuo* the crude product was purified by column chromatography (SiO_2, *i*Hex/EtOAc = 98/2). The title compound was isolated as white solid (4.426 mg, 16.7 mmol, 87 %).

HR-MS (EI) for $C_{13}H_{16}{}^{79}BrN^{\cdot+}$, $[M]^{\cdot+}$, calcd. 265.0461, found 265.0464.
^{1}H NMR ($CDCl_3$, 400 MHz): δ (ppm) = 7.02 (s, 1H, H-C(2)), 3.13 – 3.03 (m, 4H, H-C(7), H-C(12)), 2.75 – 2.65 (m, 4H, H-C(9), H-C(10)), 2.25 (s, 3H, H-C(13)), 2.04 – 1.91 (m, 4H, 9 H-C(8), H-C(11)).
^{13}C NMR ($CDCl_3$, 100 MHz): δ (ppm) = 142.8 (C(4)), 133.2 (C(6)), 130.0 (C(2)), 122.1 (C(5)), 121.6 (C(3)), 111.8 (C(1)), 50.4 (C(12)), 49.4 (C(7)), 27.5 (C(10)), 26.3 (C(9)), 22.4 (C(8)), 22.2 (C(11)), 19.2 (C(13)).
IR: $\tilde{\upsilon}$/cm^{-1} = 2921 (m), 2792 (m), 1585 (w), 1558 (m), 1478 (s), 1458 (s), 1443 (m), 1415 (s), 1387 (m), 1348 (m), 1339 (w), 1323 (m), 1300 (vs), 1203 (s), 1185 (vs), 1158 (s), 1118 (m), 1081 (m), 1052 (m), 1015 (m), 1003 (m), 894 (w), 881 (vs), 859 (m), 844 (w), 825 (w), 738 (s), 726 (m).
Melting point: 49 °C.
R_f (SiO_2, *i*hex/EtOAc = 95/5) = 0.56.

8-Methyl-9-(4,4,5,5-tetramethyl-1,3,2-dioxaborolan-2-yl)-2,3,6,7-tetrahydro-1*H*,5*H*-pyrido[3,2,1-*ij*]quinoline (6)

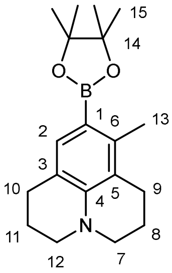

Compound **5** (1.00 g, 3.76 mmol, 1 equiv.) was dissolved in dry THF (20 mL) and cooled to -78 °C. *n*-BuLi (1.8 M in cyclohexane, 2.50 mL, 4.51 mmol, 1.2 equiv.) was added dropwise and the mixture was stirred at -78 °C for 30 min. Then, 2-isopropoxy-4,4,5,5-tetramethyl-1,3,2-dioxaborolane (0.92 mL, 4.51 mmol, 1.2 equiv.) was added and the mixture was warmed to 22 °C. H_2O (20 mL) was added, the mixture was extracted with CH_2Cl_2 (3 x 20 mL) and the combined organic phases were dried over Na_2SO_4. After removing the solvent i*n vacuo* the crude product was purified by column chromatography (SiO_2, *i*Hex/EtOAc = 99/1 → 97/3). The title compound was isolated as white solid (1.020 g, 3.27 mmol, 87 %).

HR-MS (EI) for $C_{19}H_{28}BNO^{\cdot+}$, $[M]^{\cdot+}$, calcd. 313.2208, found 313.2210.

^{1}H NMR ($CDCl_3$, 600 MHz): δ (ppm) = 7.30 (s, 1H, H-C(2)), 3.15 (t, J = 5.7 Hz, 2H, H-C(12)), 3.12 (t, J = 5.5 Hz, 2H, H-C(7)), 2.75 (t, J = 6.5 Hz, 2H, H-C(10)), 2.66 (t, J = 6.6 Hz, 2H, H-C(9)), 2.39 (s, 3H, H-C(13)), 2.02 – 1.97 (m, 2H, H-C(8)), 1.97 – 1.92 (m, 2H, H-C(11)), 1.32 (s, 12H, H-C(15)).

^{13}C NMR ($CDCl_3$, 100 MHz): δ (ppm) = 145.5 (C(4)), 141.9 (C(6)), 135.0 (C(2)), 119.9 (C(5)), 118.3 (C(3)), 82.8 (C(14)), 50.6 (C(12)), 49.6 (C(7)), 27.7 (C(10)), 25.1 (C(9)), 24.9 (C(15)), 22.3 (C(8), C(11)), 18.2 (C(13)). The signal of C(1) was not found, due to quadrupolar relaxation of the adjacent ^{11}B.

IR: $\tilde{\upsilon}$/cm^{-1} = 2972 (w), 2930 (w), 2837 (w), 2782 (w), 1593 (m), 1557 (m), 1499 (w), 1461 (w), 1434 (w), 1404 (m), 1387 (w), 1369 (m), 1357 (s), 1336 (vs), 1357 (vs), 1304 (vs), 1271 (s), 1238 (m), 1212 (m), 1182 (s), 1142 (vs), 1108 (m), 1075 (w), 1055 (m), 1028 (m), 1005 (m), 874 (m), 951 (w), 918 (w), 872 (w), 860 (m), 839 (w), 746 (m), 725 (w), 705 (w), 689 (m).

Melting point: 103 °C.

R_f (SiO_2, *i*hex/EtOAc = 95/5) = 0.44.

2-(2,2-dimethyl-1-(8-methyl-2,3,6,7-tetrahydro-1*H*,5*H*-pyrido[3,2,1-*ij*]quinolin-9-yl)propylidene)benzo[*b*]thiophen-3(2*H*)-one (7)

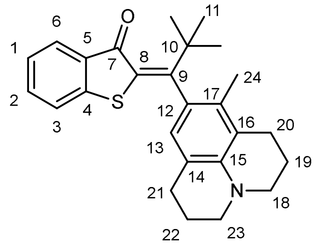

2-(1-Chloro-2,2-dimethylpropylidene)benzo[*b*]thiophen-3(2*H*)-one (**3**, 1.57 g, 6.22 mmol 1.0 equiv.) was dissolved in dioxane (18 mL). 8-Methyl-9-(4,4,5,5-tetramethyl-1,3,2-dioxaborolan-2-yl)-2,3,6,7-tetrahydro-1*H*,5*H*-pyrido[3,2,1-*ij*]quinoline (**6**, 1.95 g, 6.22 mmol, 1.0 equiv.), KO*t*Bu (1.72 g, 12.5 mmol, 2.0 equiv.) and H_2O (2 mL) were added. After bubbling argon through the solution for 10 min, $Pd(PPh_3)_4$ (360 mg, 0.31 mmol, 5 mol%) was added and the reaction mixture was stirred at 90 °C for 6 h. Subsequently, a saturated sodium carbonate solution (20 mL) was added, the aqueous phase was extracted with CH_2Cl_2 (3 x 50 mL), and the combined organic phases were dried over Na_2SO_4. After removing the solvent i*n vacuo* the crude product was purified by column chromatography (SiO_2, *i*Hex/EtOAc = 99/1 → 9/1). The title compound was isolated as red resin (550 mg, 0.22 mmol, 22 %).

HR-MS (EI) for $C_{26}H_{29}NO^{32}S^{\cdot+}$, $[M]^{\cdot+}$, calcd. 403.1964, found 403.1960.

^{1}H NMR ($CDCl_3$, 400 MHz): δ (ppm) = 7.80 (dd, J = 7.8, 1.4 Hz, 1H, H-C(6)), 7.45 – 7.39 (m, 1H, H-C(2)), 7.22 – 7.13 (m, 2H, H-C(1), H-C(3)), 6.41 (s, 1H, H-C(13)), 3.19 – 3.08 (m, 4H, H-C(18), H-C(23)), 2.80 – 2.70 (m, 2H, H-C(21)), 2.70 – 2.60 (m, 2H, H-C(20)), 2.11 – 1.94 (m, 7H, H-C(20), H-C(22), H-C(24)), 1.34 (s, 9H, H-C(11)).

^{13}C NMR ($CDCl_3$, 100 MHz): δ (ppm) = 186.4 (C(7)), 167.8 (C(9)), 146.4 (C(4)), 142.7 (C(15)), 134.7 (C(8)), 134.4 (C(0)), 134.1 (C(5)), 133.5 (C(12)), 129.8 (C(17)), 126.8 (C(6)), 124.5 (C(13)), 124.4 (C(1)), 122.8 (C(3)), 119.9 (C(16)), 119.1 (C(14)), 50.6 (C(23)), 49.7 (C(18)), 39.3 (C(10)), 29.1 (C(11)), 27.8 (C(21)), 25.1 (C(20)), 22.4 (C(22)), 22.3 (C(19)), 17.3 (C(24)).

IR: $\tilde{\upsilon}$/cm^{-1} = 2927 (s), 2856 (m), 1664 (vs), 1588 (s), 1531 (m), 1491 (m), 1642 (s), 1450 (vs), 1391 (w), 1376 (w), 1360 (m), 1326 (m), 1306 (s), 1279 (vs), 1213 (s), 1188 (w), 1157 (w), 1135 (w), 1073 (s), 1062 (m), 1042 (w), 1019 (w), 995 (w), 924 (w), 907 (w), 887 (w), 862 (w), 797 (w), 774 (w), 744 (s), 734 (s), 681 (w).

R$_f$ (SiO_2, *i*hex/EtOAc = 95/5) = 0.44.

2-(2,2-Dimethyl-1-(8-methyl-2,3,6,7-tetrahydro-1*H*,5*H*-pyrido[3,2,1-*ij*]quinolin-9-yl)propylidene)benzo[*b*]thiophen-3(2*H*)-one 1-oxide (1)

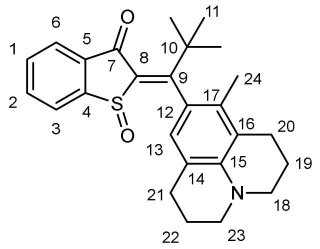

Compound **7** (550 mg, 1.36 mmol, 1 equiv.) was dissolved in a mixture of 4 mL CH_2Cl_2 and 2 mL TFA. *Meta*-chloroperoxybenzoic acid (235 mg, 1.36 mmol, 1 equiv.) was added at 0 °C in portions and the solution was further stirred for 10 min at 0 °C. Subsequently, a saturated sodium carbonate solution was added until the pH was adjusted to 7, the aqueous phase was extracted with CH_2Cl_2 (3 x 50 mL), and the combined organic phases were dried over Na_2SO_4. After removing the solvent i*n vacuo* the crude product was purified by column chromatography (SiO_2, *i*hex/EtOAc = 7/3 → 1/1). The isomers **1-A** and **1-D** were further purified by HPLC (*Chiralpak* IC Semiprep column, *n*heptane/EtOAc = 7/3). All isomers were separately crystallized from *n*heptane/EtOAc. The four isomers of the title compound were isolated as orange to red crystals (405 mg, 0.97 mmol, 71 %, sum of all isomers).

1-A

^{1}**H NMR** ($CDCl_3$, 400 MHz): δ (ppm) = 7.96 (dt, J = 7.6, 1.0 Hz, 1H, H-C(6)), 7.81 (dt, J = 7.7, 0.9 Hz, 1H, H-C(3)), 7.75 (td, J = 7.4, 1.3 Hz, 1H, H-C(2)), 7.66 (td, J = 7.4, 1.2 Hz, 1H, H-C(1)), 6.35 (s, 1H, H-C(13)), 3.19 – 3.08 (m, 4H, H-C(18), H-C(23)), 2.77 – 2.57 (m, 4H, H-C(20), H-C(21)), 2.10 – 1.93 (m, 7H, H-C(19), H-C(22), H-C(24)), 1.35 (s, 9H, H-C(11)).

^{13}**C NMR** ($CDCl_3$, 100 MHz): δ (ppm) = 185.1 (C(9)), 184.8 (C(7)), 148.7 (C(4)), 143.4 (C(15)), 143.1 (C(8)), 136.4 (C(5)), 135.5 (C(2)), 132.5 (C(1)), 132.2 (C(17)), 129.7 (C(12)), 127.1 (C(3)), 126.2 (C(6)), 124.4 (C(13)), 119.9 (C(16)), 118.9 (C(14)), 50.5 (C(23)), 49.6 (C(18)), 40.5 (C(13)), 29.1 (C(11)), 27.9 (C(21)), 24.9 (C(20)), 22.4 (C(22)), 22.2 (C(19)), 19.7 (C(24)).

IR: $\tilde{\upsilon}$/cm^{-1} = 2942 (m), 2858 (w), 1679 (vs), 1587 (m), 1548 (s), 1490 (s), 1459 (s), 1448 (m), 1392 (w), 1374 (w), 1353 (w), 1325 (w), 1305 (vs), 1273 (s), 1214 (vs), 1188 (m), 1157 (w), 1134 (w), 1119 (w), 1059 (m), 1028 (vs), 926 (m), 906 (w), 860 (w), 799 (w), 767 (m), 748 (vs), 739 (s), 699 (m), 684 (s), 663 (m).

Melting point: 190 °C.

$\mathbf{R_f}$ (SiO_2, *i*hex/EtOAc = 6/4) = 0.35.

1-B

^{1}H NMR ($CDCl_3$, 599 MHz): δ (ppm) = 7.95 (dt, J = 7.6, 1.0 Hz, 1H, H-C(6)), 7.79 (dt, J = 7.7, 1.0 Hz, 1H, H-C(3)), 7.75 (td, J = 7.4, 1.2 Hz, 1H, H-C(2)), 7.67 (td, J = 7.4, 1.2 Hz, 1H, H-C(1)), 6.55 (s, 1H, H-C(13)), 3.22 – 3.08 (m, 4H, H-C(18), H-C(13)), 2.87 – 2.79 (m, 1H, H-C(21)), 2.75 – 2.68 (m, 1H, H-C(21)), 2.64 (t, J = 6.7 Hz, 2H, H-C(20)), 2.07 – 1.95 (m, 4H, H-C(19), H-C(22)), 1.94 (s, 3H, H-C(24)), 1.34 (s, 9H, H-C(11)).

^{13}C NMR ($CDCl_3$, 151 MHz): δ (ppm) = 185.9 (C(7)), 183.7 (C(9)), 148.8 (C(4)), 143.4 (C(15)), 142.5 (C(8)), 136.5 (C(5)), 135.6 (C(2)), 132.5 (C(1)), 129.0 (C(17)), 128.1 (C(12)), 127.2 (C(3)), 127.0 (C(13)), 126.3 (C(6)), 119.8 (C(16)), 118.3 (C(14)), 50.5 (C(23)), 49.6 (C(18)), 40.3 (C(10)), 29.5 (C(11)), 27.8 (C(21)), 25.1 (C(20)), 22.3 (C(19), C(22)), 17.9 (C(24)).

IR: $\tilde{\upsilon}$/cm^{-1} = 2942 (w), 1683 (vs), 1589 (s), 1554 (s), 1493 (s), 1462 (s), 1393 (m), 1360 (m), 1323 (s), 1305 (vs), 1272 (s), 1214 (vs), 1185 (s), 1156 (w), 1134 (m), 1119 (m), 1060 (m), 1034 (vs), 994 (w), 924 (m), 902 (w), 845 (w), 801 (w), 755 (vs), 729 (vs), 697 (s), 688 (vs), 661 (m).

Melting point: 123 °C.

R_f (SiO_2, *i*hex/EtOAc = 6/4) = 0.26.

1-C

^{1}H NMR ($CDCl_3$, 599 MHz): δ (ppm) = 8.03 (dt, J = 7.3, 1.0 Hz, 1H, H-C(3)), 7.83 – 7.79 (m, 2H, H-C(2), H-C(6)), 7.64 (td, J = 7.5, 1.0 Hz, 1H, H-C(1)), 6.31 (s, 1H, H-C(13)), 3.18 – 3.04 (m, 4H, H-C(18), H-C(23)), 2.80 – 2.59 (m, 4H, H-C(20), H-C(21)), 2.11 – 1.94 (m, 4H, H-C(19), H-C(22)), 1.87 (s, 3H, H-C(24)), 1.47 (s, 9H, H-C(11)).

^{13}C NMR ($CDCl_3$, 151 MHz): δ (ppm) = 184.9 (C(7)), 180.3 (C(11)), 147.6 (C(4)), 142.6 (C(15)), 139.4 (C(8)), 135.6 (C(2)), 134.6 (C(5)), 132.7 (C(1)), 130.1 (C(17)), 128.3 (C(12)), 127.1 (C(3)), 126.0 (C(6)), 122.9 (C(13)), 119.9 (C(16)), 118.9 (C(14)), 50.6 (C(23)), 49.7 (C(18)), 41.9 (C(10)), 30.3 (C(11)), 27.7 (C(21)), 25.0 (C(20)), 22.5 (C(25), C(28)), 18.0 (C(24)).

IR: $\tilde{\upsilon}$/cm^{-1} = 2928 (m), 2803 (w), 2773 (w), 1693 (s), 1591 (m), 1580 (m), 1545 (s), 1488 (s), 1454 (s), 1391 (w), 1373 (w), 1358 (w), 1326 (m), 1304 (s), 1272 (vs), 1189 (s), 1157 (s), 1120 (s), 1107 (m), 1070 (m), 1055 (w), 1033 (vs), 997 (s), 962 (w), 891 (w), 874 (w), 852 (w), 796 (w), 756 (vs), 735 (m), 713 (m), 701 (w), 682 (s), 655 (s).

Melting point: 184 °C.

R_f (SiO_2, *i*hex/EtOAc = 6/4) = 0.50.

1-D

^{1}H NMR ($CDCl_3$, 400 MHz): δ (ppm) = 7.99 (dd, J = 8.1, 1.1 Hz, 1H, H-C(3)), 7.82 – 7.77 (m, 2H, H-C(2), H-C(6)), 7.65 (td, J = 7.4, 1.0 Hz, 1H, H-C(1)), 6.12 (s, 1H, H-C(13)), 3.19 – 3.05 (m, 4H, H-C(18), H-C(23)), 2.76 – 2.59 (m, 4H, H-C(20), H-C(21)), 2.09 – 1.92 (m, 7H, H-C(19), H-C(22), H-C(24)), 1.46 (s, 9H, H-C(11)).

^{13}C NMR ($CDCl_3$, 101 MHz): δ (ppm) = 187.0 (C(7)), 178.7 (C(11)), 147.5 (C(4)), 142.6 (C(15)), 139.0 (C(8)), 135.6 (C(2)), 134.2 (C(5)), 133.0 (C(1)), 130.8 (C(17)), 127.9 (C(12)), 127.3 (C(3)), 126.3 (C(6)), 122.4 (C(13)), 120.0 (C(16)), 118.7 (C(14)), 50.6 (C(23)), 49.7 (C(18)), 41.5 (C(10)), 31.7 (C(11)), 27.8 (C(20)), 25.0 (C(21)), 22.4 (C(19), C(22)), 17.4 (C(24)).

IR: $\tilde{\upsilon}$/cm^{-1} = 2923 (m), 1694 (vs), 1592 (m), 1528 (m), 1495 (m), 1463 (s), 1450 (m), 1396 (w), 1357 (w), 1325 (m), 1307 (s), 1277 (s), 1215 (vs), 1188 (m), 1156 (m), 1134 (m), 1123 (m), 1109 (m), 1071 (m), 1034 (vs), 997 (m), 889 (w), 850 (w), 797 (w), 759 (vs), 733 (m), 712 (w), 700 /m), 681 (s), 681 (m).

Melting point: 176 °C.

R_f (SiO_2, *i*hex/EtOAc = 6/4) = 0.38.

For all isomers:

HR-MS (EI) for $C_{26}H_{29}NO_2{}^{32}S^{\cdot+}$, $[M]^{\cdot+}$, calcd. 419.1914, found 419.1898.

Physical and photophysical properties

Thermal isomerizations

Amberized NMR tubes were charged with 1 mg to 2.5 mg of the respective isomer **A**, **B**, **C**, or **D** and 0.7 mL of 1,2-dichlorobenzene-d_4, DMSO-d_6, or MeCN-d_4/D_2O: 8/2. Subsequently the NMR tubes were heated to the appropriate temperatures for isomerization reactions to occur and the kinetics were followed by ^{1}H NMR measurements in defined time intervals. The unchanging equilibrium ratio of isomers after prolonged heating were obtained from integration of the corresponding signals in the ^{1}H NMR spectrum.

The kinetics of thermal isomer interconversions differ strongly in the different solvents, however the most prominent isomerization is the thermal Hula-Twist isomerization of *E* configured **C** and **D** to *Z* configured **B** and **A**, respectively in all solvents. These isomerizations are unimolecular first order reactions and proceed towards an equilibrium composition with only the two interconverting isomers present. The kinetics can therefore generally expressed for the reaction **X→Y** according to Supplementary Equation 1.

$$\ln\left(\frac{[\mathbf{X}]_{t_0}-[\mathbf{X}]_{\mathrm{eq}}}{[\mathbf{X}]_{t}-[\mathbf{X}]_{\mathrm{eq}}}\right) = (k_{\mathbf{X/Y}} + k_{\mathbf{Y/X}})t \qquad \text{(Supplementary Equation 1)}$$

With $[\mathbf{X}]_{t_0}$ being the initial concentration of the starting isomer **X** at the time $t = 0$, $[\mathbf{X}]_{\mathrm{eq}}$ being the concentration of the isomer **X** at equilibrium, $[\mathbf{X}]_t$ representing the concentration of the isomer **X** at a specific time t during the measurement, $k_{\mathbf{X/Y}}$ being the rate constant k of the **X** to **Y** conversion, $k_{\mathbf{Y/X}}$ being the rate constant k of the **Y** to **X** conversion, and t being the elapsed time. When plotting the logarithmic left part of Supplementary Equation 1 versus time t the obtained slope m contains the rate constants for both isomerization reactions taking place. The rate constant can then be calculated according to Supplementary Equation 2:

$$k_{\mathbf{X/Y}} = \frac{m}{1+\frac{[\mathbf{X}]_{\mathrm{eq}}}{[\mathbf{Y}]_{\mathrm{eq}}}} \qquad \text{(Supplementary Equation 2)}$$

when taking into account the corresponding law of mass action (Supplementary Equation 3):

$$\frac{[\mathbf{X}]_{\mathrm{eq}}}{[\mathbf{Y}]_{\mathrm{eq}}} = \frac{k_{\mathbf{Y/X}}}{k_{\mathbf{X/Y}}} \qquad \text{(Supplementary Equation 3)}$$

Likewise the reverse rate constant is defined and can be determined from separate measurements starting from the opposite isomer Y.

By using the *Eyring* equation (Supplementary Equation 4) the free activation enthalpies ΔG^* can be calculated from the rate constants of the corresponding reaction.

$$\Delta G^* = \ln\left(\frac{k*h}{k_B*T}\right) * R * T \qquad \text{(Supplementary Equation 4)}$$

The obtained free activation enthalpies ΔG^* for the thermal isomerizations between **A** to **B**, **C** to **D**, and *vice versa* in the different solvents as well as the corresponding extrapolated half-lives at 25 °C are given in Table S1 together with the equilibrium compositions obtained at high temperatures.

However in nonpolar solvents like 1,2-dichlorobenzene-d_4 not only the thermal hula twist reaction occurs but also single bond rotations and double bond isomerizations. To model the kinetics of this more complicated scenario the Markov matrix approach from our previous publication[2] was used by replacing the probability elements of the matrix with the converted *Eyring* equation (Supplementary Equation5).

$$M_1 = \begin{pmatrix} k_{AA} & k_{AB} & k_{AC} & k_{AD} \\ k_{BA} & k_{BB} & k_{BC} & k_{BD} \\ k_{CA} & k_{CB} & k_{CC} & k_{CD} \\ k_{DA} & k_{DB} & k_{DC} & k_{DD} \end{pmatrix} \quad \text{with } k_{AA} = 1 - (k_{AB} + k_{AC} + k_{AD}) \; \ldots \; \text{and } k_{AB} = \frac{k_B \cdot T}{h} \cdot e^{\frac{-\Delta G^{\ddagger}_{AB}}{R \cdot T}} \; \ldots$$

(Supplementary Equation 5)

The kinetics were then modelled by multiplication of M_1 with the concentrations of the isomers **A**, **B**, **C**, **D** to give the corresponding concentration vector of the next time point (Supplementary Equation6). The sum of the concentrations of the isomers were previously normalized to 100 to enable an easier presentation of the data.

$$\begin{pmatrix} [A] \\ [B] \\ [C] \\ [D] \end{pmatrix}_{t_{x+1}} = \begin{pmatrix} k_{AA} & k_{AB} & k_{AC} & k_{AD} \\ k_{BA} & k_{BB} & k_{BC} & k_{BD} \\ k_{CA} & k_{CB} & k_{CC} & k_{CD} \\ k_{DA} & k_{DB} & k_{DC} & k_{DD} \end{pmatrix} \cdot \begin{pmatrix} [A] \\ [B] \\ [C] \\ [D] \end{pmatrix}_{t_x}$$

(Supplementary Equation 6)

The different ΔG^{*} values for each isomerization were optimized against a refinement factor R, which was calculated according to Supplementary Equation 7.

$$R = \frac{\sum\frac{[\mathrm{Xm}]_t}{[\mathrm{Xc}]_t}}{x} \qquad \text{(Supplementary Equation7)}$$

With [**X**m]$_t$ being the measured concentration of **X** at time *t*, [**Xc**]$_t$ being the calculated concentration of **X** at time *t*, and *x* being the total amount of acquired measurement points. The closer *R* becomes to 1 the better is the obtained fit of the data. Measurement points below a concentration of 0.01 were not taken into account for the calculation of the *R* factor as the error of integration might be very high at low concentrations.
From these measurements we could quantify the full ground state energy profile of HTI **1** in 12DCB. The equilibrium isomer composition at high temperatures delivers the energy differences between the corresponding states according to the relation of the change of *Gibbs* free energy and the equilibrium constant.

Quantum yield measurements in different solvents

The photochemical quantum yields of the different photoconversion reactions ϕ were calculated as the ratio between the numbers of isomerized molecules *n*(molecules isomerized) and the number of absorbed photons *n*(*hv*) according to Supplementary Equation 8:

$$\phi = \frac{n(\text{molecules isomerized})}{n(hv)} \qquad \text{(Supplementary Equation 8)}$$

To determine the quantum yields ϕ, a sample with known concentration of each pure isomer **A**, **B**, **C**, or **D** in different solvents was irradiated with a focused light beam of a 520 nm LED within the published instrumental setup from the group of *E. Riedle*.[3] The number of absorbed photons over time *n*(*hv*) was measured directly at the thermal photometer of the instrument according to Supplementary Equation 9:

$$n(hv) = \frac{\Delta P \cdot \lambda_{\mathrm{ex}} \cdot t}{c \cdot h} \qquad \text{(Supplementary Equation 9)}$$

Where *c* is the speed of light ($2.99792 \cdot 10^{8}$ m·s^{-1}), *h* is Planck's constant ($6.62607 \cdot 10^{-34}$ J·s), λ_{ex} is the excitation wavelength in m, *t* is the elapsed time during irradiation, and ΔP is the difference in power read-outs at the thermal photometer between a solvent filled cuvette (P_0) and a cuvette containing the sample solution (P_t) during the irradiation period in Watt (Supplementary Equation 10). The power read out during

irradiation did not change substantially (<5%) over the irradiation periods in the control experiment with the solvent filled cuvette.

$$\Delta P = P_t - P_0 \qquad \text{(Supplementary Equation 10)}$$

Since more than one photoproduct can be formed during irradiation, the number of each type of photoconverted molecules *n*(molecules isomerized) was determined by ^{1}H NMR spectroscopy directly after the irradiation step. Multiple measurements with increasing time of irradiation were conducted and the obtained ϕ values were averaged.

Theoretical description of the ground state of 1

A relaxed optimization of the four isomeric states and all four transition state structures of compound **1** at the ω–B97XD/6-311G(d,p) level of theory has been conducted using the Gaussian16 Revision A.03 program package.[4] To account for solvent effects, the calculations have been carried out using the Polarizable Continuum Model (PCM) with DMSO parameters. The convergence criteria have been set tight and an ultrafine integration grid has been used. Frequency analysis confirmed four structures (**A**, **B**, **C**, **D**) to be minimum structures since no imaginary frequencies have been found. All other structures were shown to be first order saddle points on the potential energy surface since only one imaginary vibrational mode has been found confirming them to be transition state structures.

Crystal structure analysis

All X-ray intensity data were measured on a Bruker D8 Venture TXS system equipped with a multilayer mirror optics monochromator and a Mo Kα rotating-anode X-ray tube (λ = 0.71073 Å). The data collections were performed at 103 K. The frames were integrated with the Bruker SAINT Software package [5]. Data were corrected for absorption effects using the Multi-Scan method (SADABS) [6]. The structures were solved and refined using the Bruker SHELXTL Software Package [7]. All C-bound hydrogen atoms were calculated in positions having ideal geometry riding on their parent atoms. The data have been deposited with the Cambridge Crystallographic Data Centre (CCDC) and can be obtained free of charge from: https://www.ccdc.cam.ac.uk/structures/. The deposition numbers are: CCDC 1910546 (**A-1**), CCDC 1910547 (**B-1**), CCDC 1910548 (**C-1**) and CCDC 1910549 (**D-1**).

Supplementary Figures

Determination of constitutions and conformations in the crystalline state and in solution

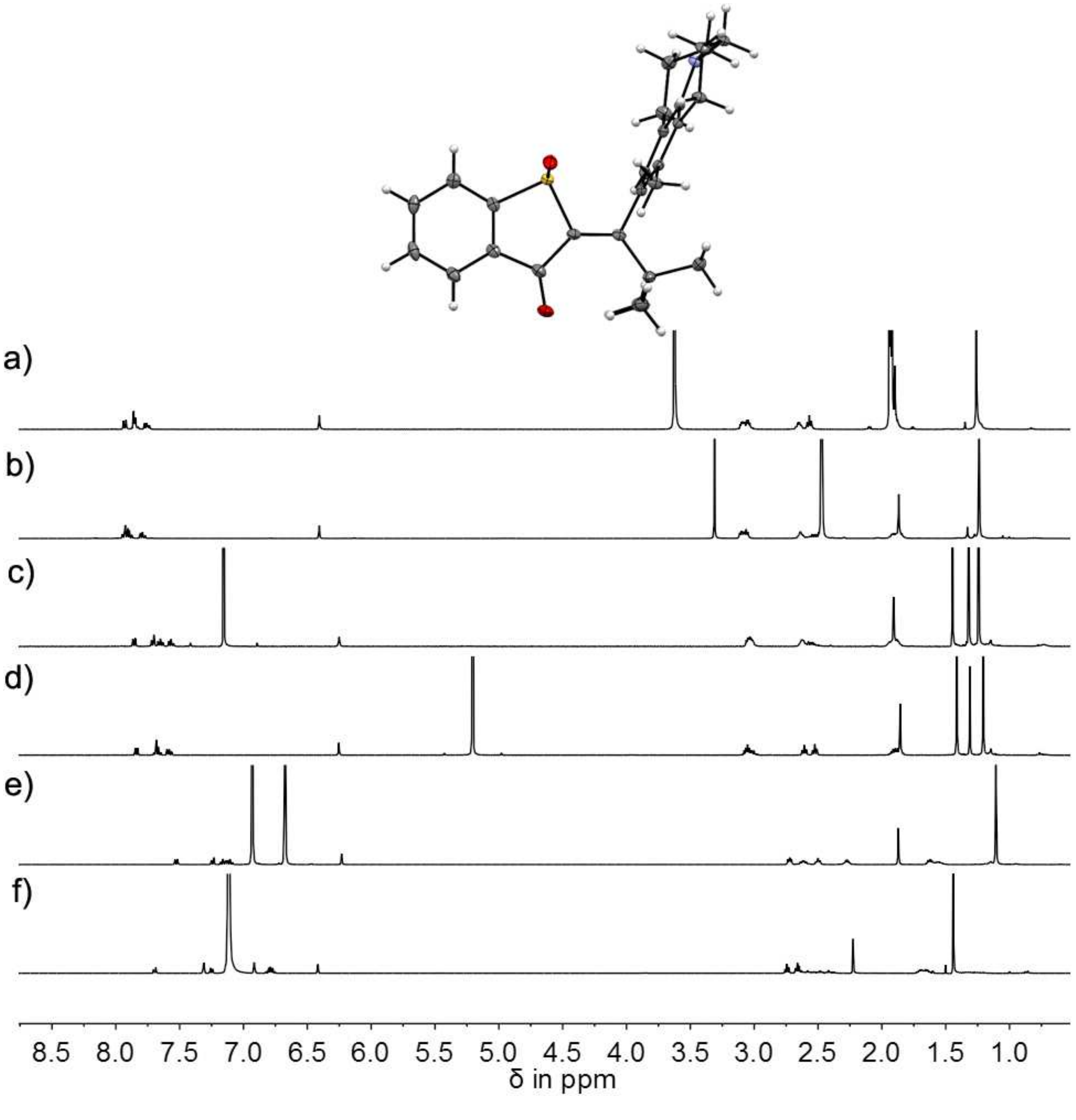

Supplementary Figure 1 | Structure assignment of A-1. Structure of **A-1** in the crystalline state and the corresponding ^{1}H NMR solution spectra (400 MHz, 20 °C) obtained from the same crystal batch in: a) MeCN-d_4/D_2O: 8/2, b) DMSO-d_6, c) $CDCl_3$, d) CD_2Cl_2, e) 1,2-dichlorobenzene-d_4, f) benzene-d_6.

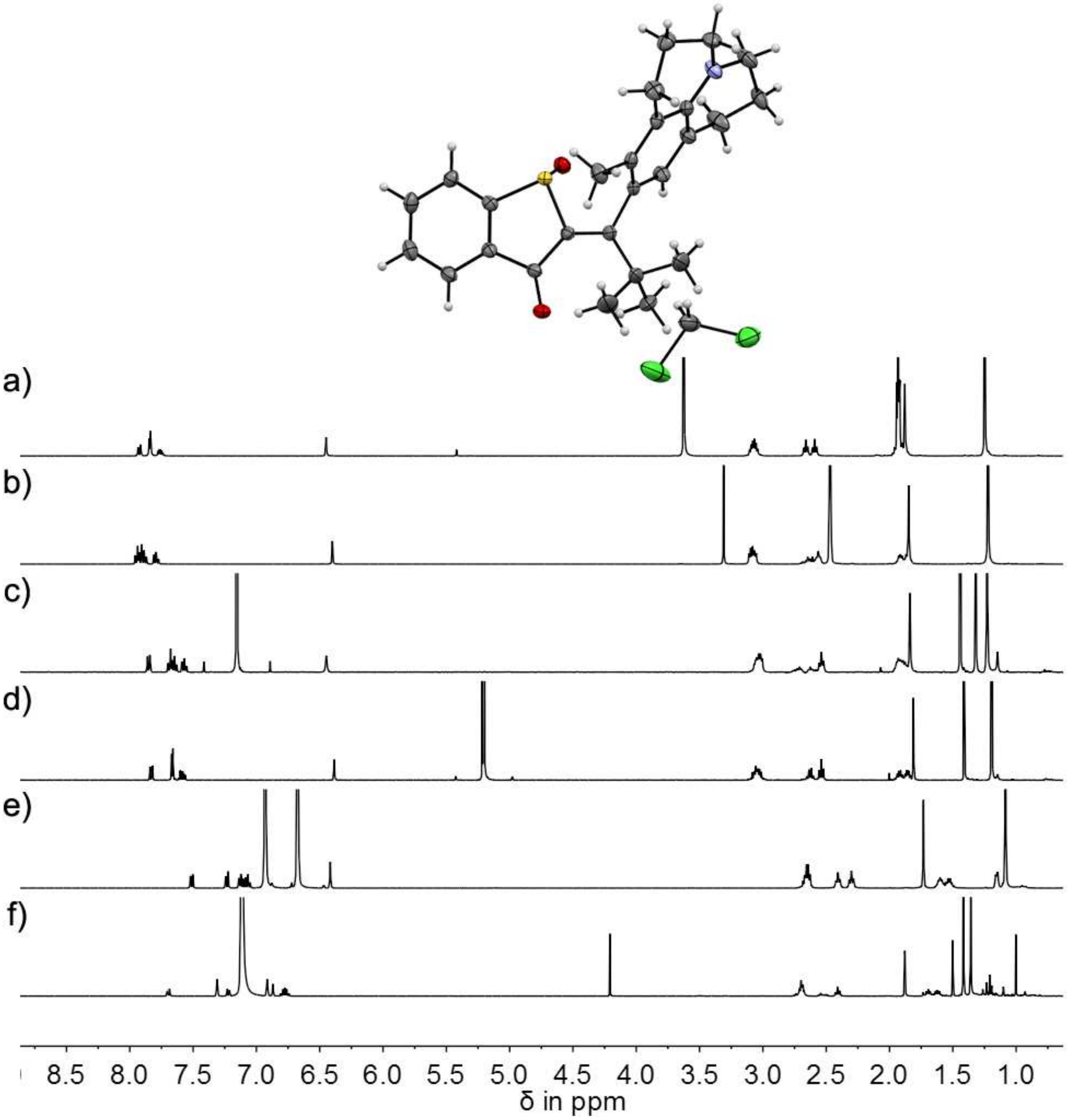

Supplementary Figure 2 | Structure assignment of B-1. Structure of **B-1** in the crystalline state and the corresponding ^{1}H NMR solution spectra (400 MHz, 20 °C) obtained from the same crystal batch in: a) MeCN-d_4/D_2O: 8/2, b) DMSO-d_6 (co-crystallized CH_2Cl_2 was removed by evaporation), c) $CDCl_3$ (co-crystallized CH_2Cl_2 was removed by evaporation), d) CD_2Cl_2, e) 1,2-dichlorobenzene-d_4 (co-crystallized CH_2Cl_2 was removed by evaporation), f) benzene-d_6.

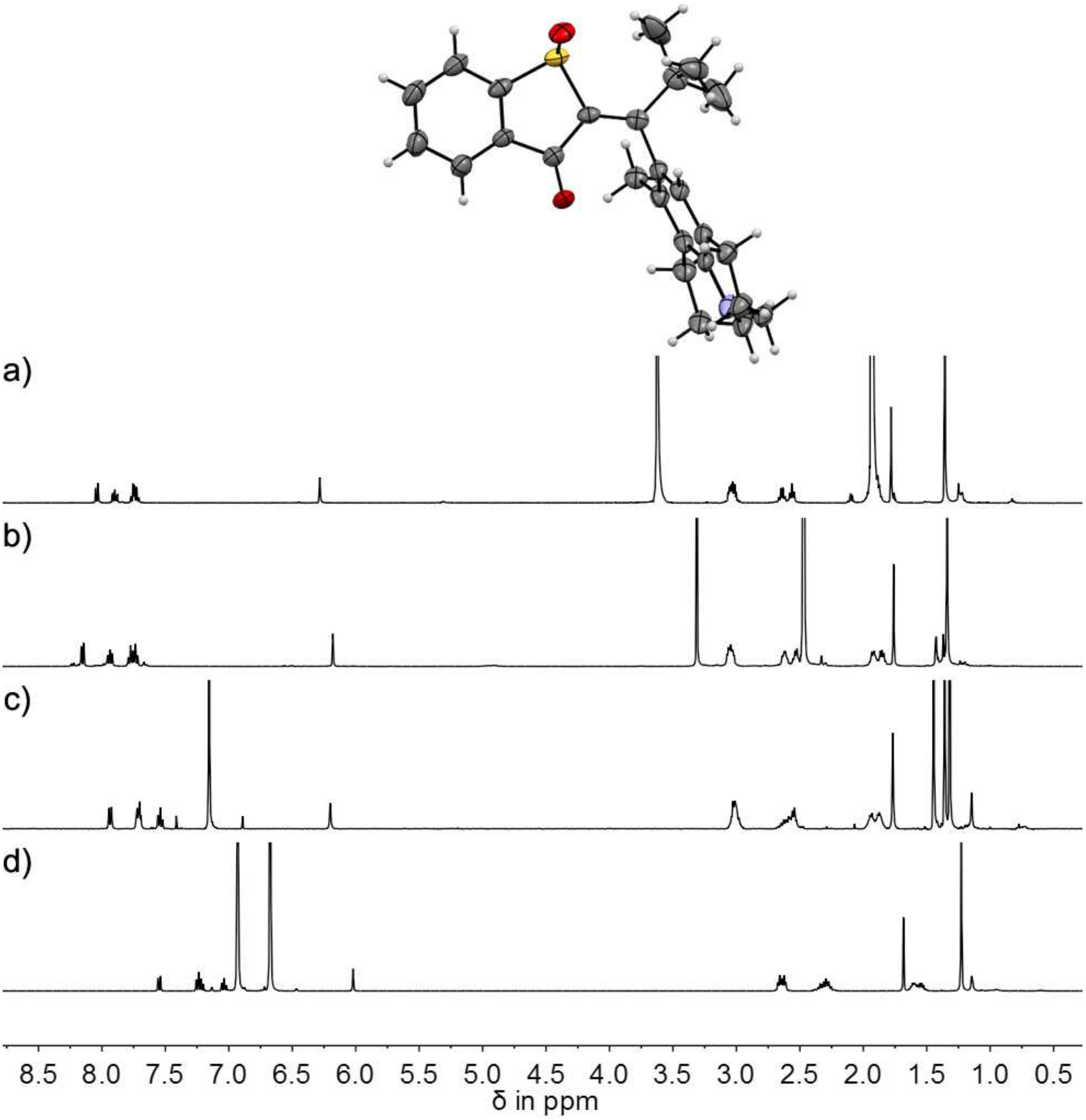

Supplementary Figure 3 | Structure assignment of C-1. Structure of **C-1** in the crystalline state and the corresponding ^{1}H NMR solution spectra (400 MHz, 20 °C) obtained from the same crystal batch in: a) MeCN-d_4/D_2O: 8/2, b) DMSO-d_6, c) $CDCl_3$, d) 1,2-dichlorobenzene-d_4.

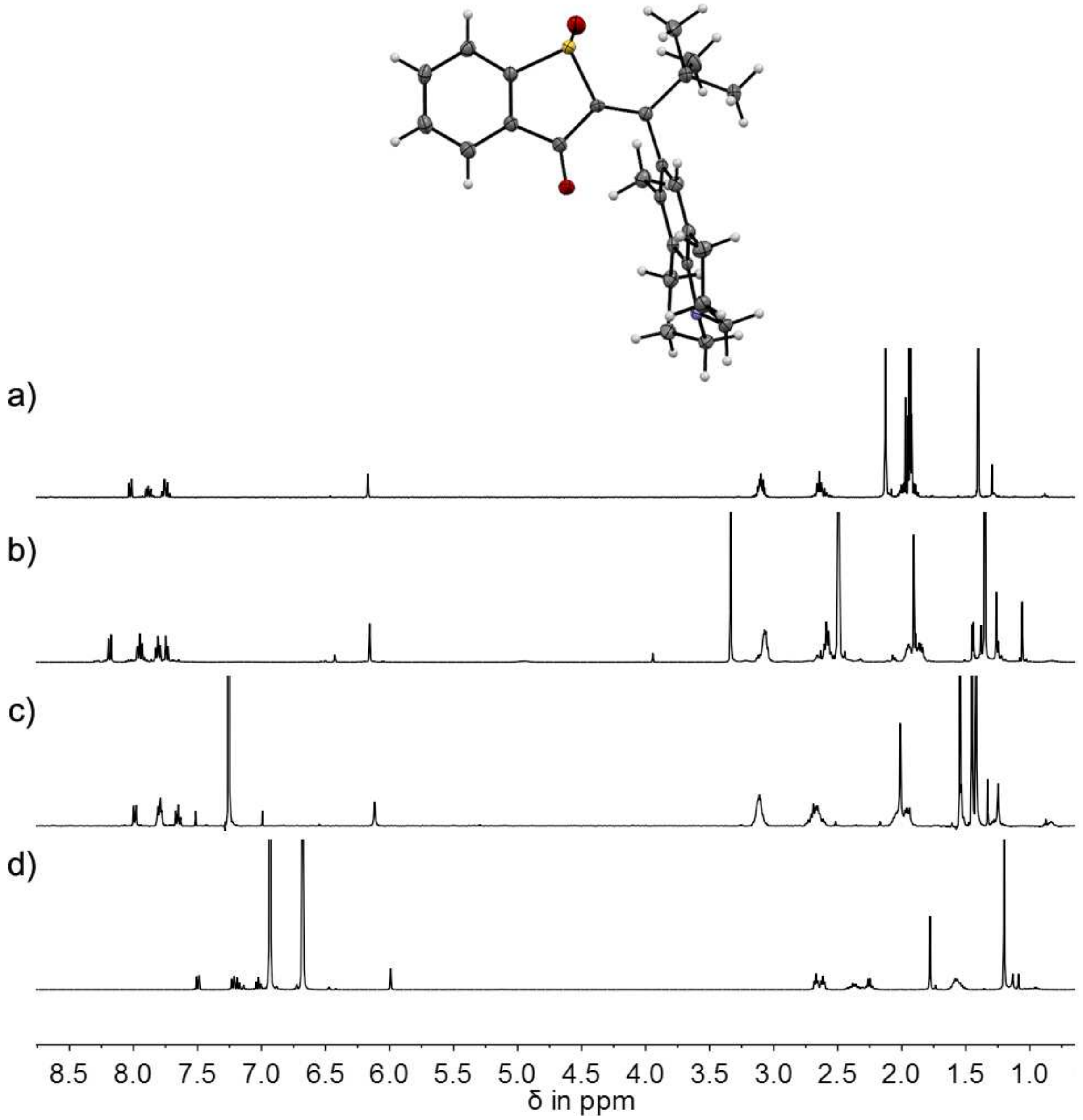

Supplementary Figure 4 | Structure assignment of D-1. Structure of **D-1** in the crystalline state and the corresponding ^{1}H NMR solution spectra (400 MHz) obtained from the same crystal batch in: a) MeCN-*d*$_4$ (27 °C), b) DMSO-*d*$_6$ (20 °C), c) $CDCl_3$ (20 °C), d) 1,2-dichlorobenzene-*d*$_4$ (20 °C).

Thermal isomerizations

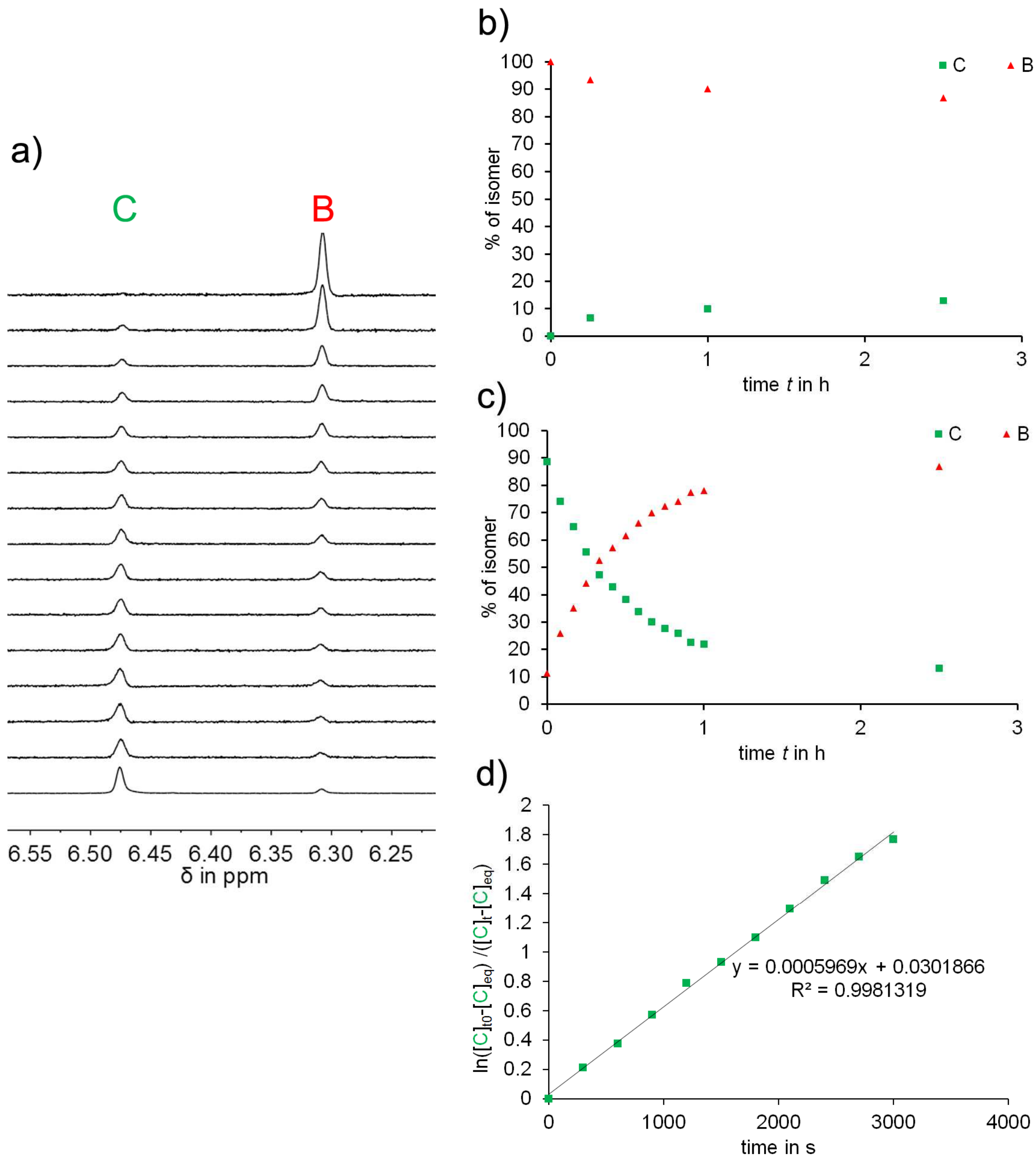

Supplementary Figure 5 | Thermal isomerization between C and B in the dark. a) Thermal isomerization of **C** to **B** in MeCN-d_4/D_2O: 8/2 at 60 °C followed by ^{1}H NMR spectroscopy (400 MHz, 60 °C) in regular time intervals. b) Thermal isomer conversion from **B** to **C** used as control experiment for determination of the isomer composition in thermal equilibrium. These data were not used to determine ΔG^* values as the changes in concentration are very smallc) Thermal isomer conversion from **C** to **B**. d) First order kinetic analysis of the thermal isomerization of **C** to **B**. Taking into account the dynamic equilibrium by plotting according to Supplementary Equation 1 gives a linear relationship. The slope m can be translated into the rate constant $k_{(\mathbf{C/B})}$ according to Supplementary Equation 2. The corresponding Gibbs free energy of activation ΔG^* for thermal **C** to **B** isomerization is given in Supplementary Table 1.

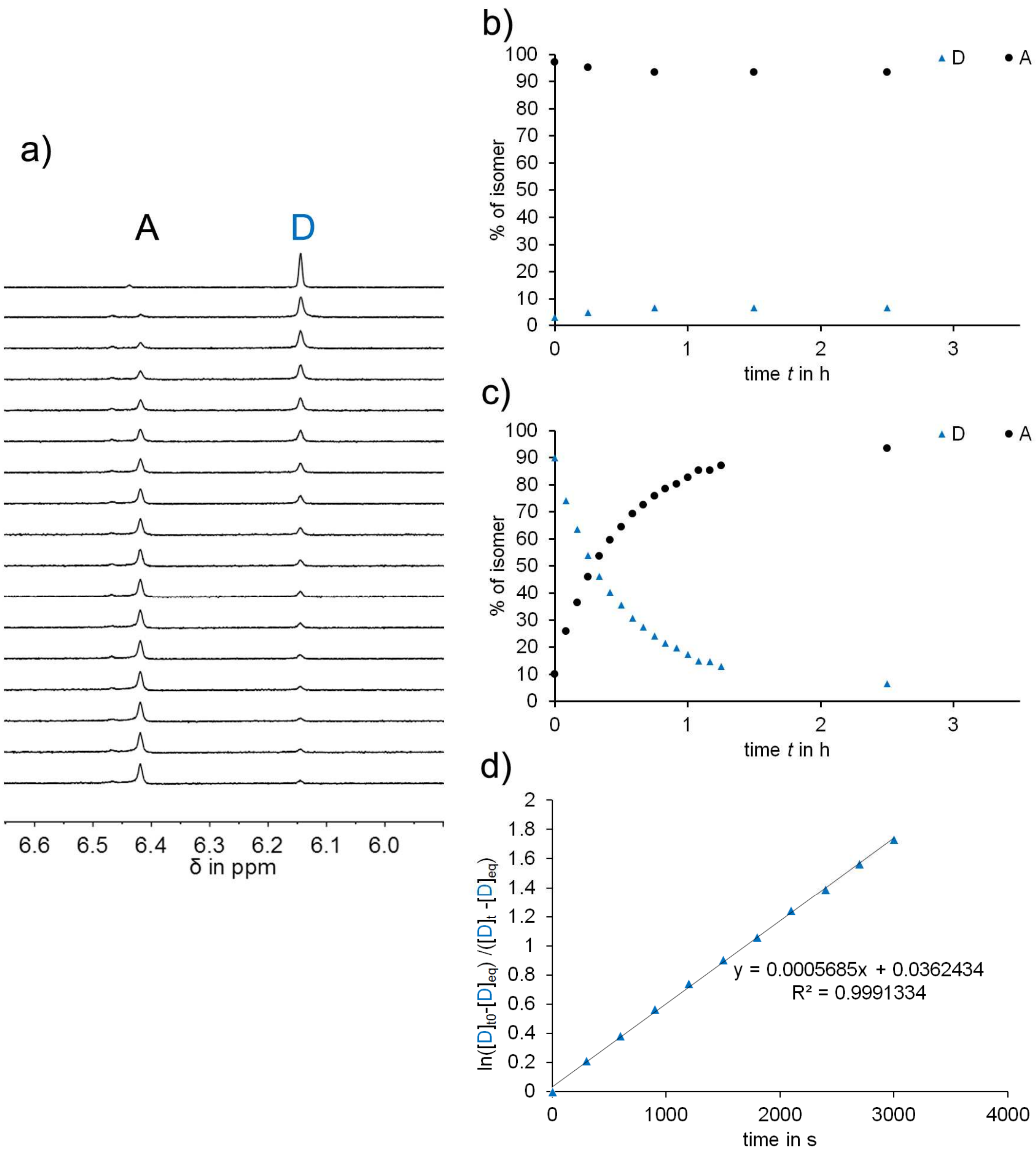

Supplementary Figure 6 | Thermal isomerization between D and A in the dark. a) Thermal isomerization of **D** to **A** in MeCN-d_4/D_2O: 8/2 at 27 °C followed by ^{1}H NMR spectroscopy (400 MHz, 27 °C) in regular time intervals. b) Thermal isomer conversion from **A** to **D** used as control experiment for determination of the isomer composition in thermal equilibrium. These data were not used to determine ΔG^* values as the changes in concentration are very small. c) Thermal isomer conversion from **D** to **A**. d) First order kinetic analysis of the thermal isomerization of **D** to **A**. Taking into account the dynamic equilibrium by plotting according to Supplementary Equation 1 gives a linear relationship. The slope m can be translated into the rate constant $k_{(\mathbf{D/A})}$ according to Supplementary Equation 2. The corresponding Gibbs free energy of activation ΔG^* for thermal **D** to **A** isomerizations is given in Supplementary Table 1.

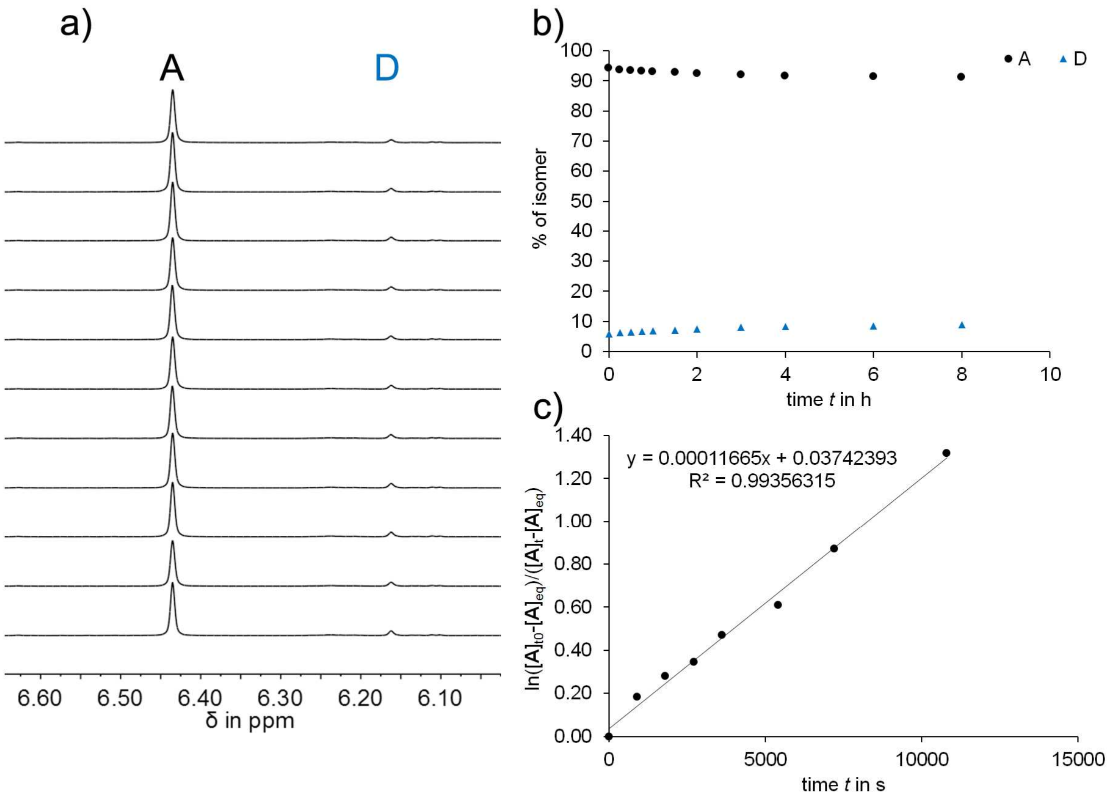

Supplementary Figure 7 | Thermal isomerization from A to D in the dark. a) Thermal isomerization of **A** to **D** in DMSO-d_6 at 60 °C followed by ^{1}H NMR spectroscopy (400 MHz, 20 °C) in regular time intervals. b) Thermal isomer conversion from **A** to **D**. c) First order kinetic analysis of the thermal isomerization of **A** to **D**. Taking into account the dynamic equilibrium by plotting according to Supplementary Equation 1 gives a linear relationship. The slope m can be translated into the rate constant $k_{(\mathbf{A/D})}$ according to Supplementary Equation 2. The corresponding Gibbs free energy of activation ΔG^* for the thermal **A** to **D** isomerization is given in Supplementary Table 2.

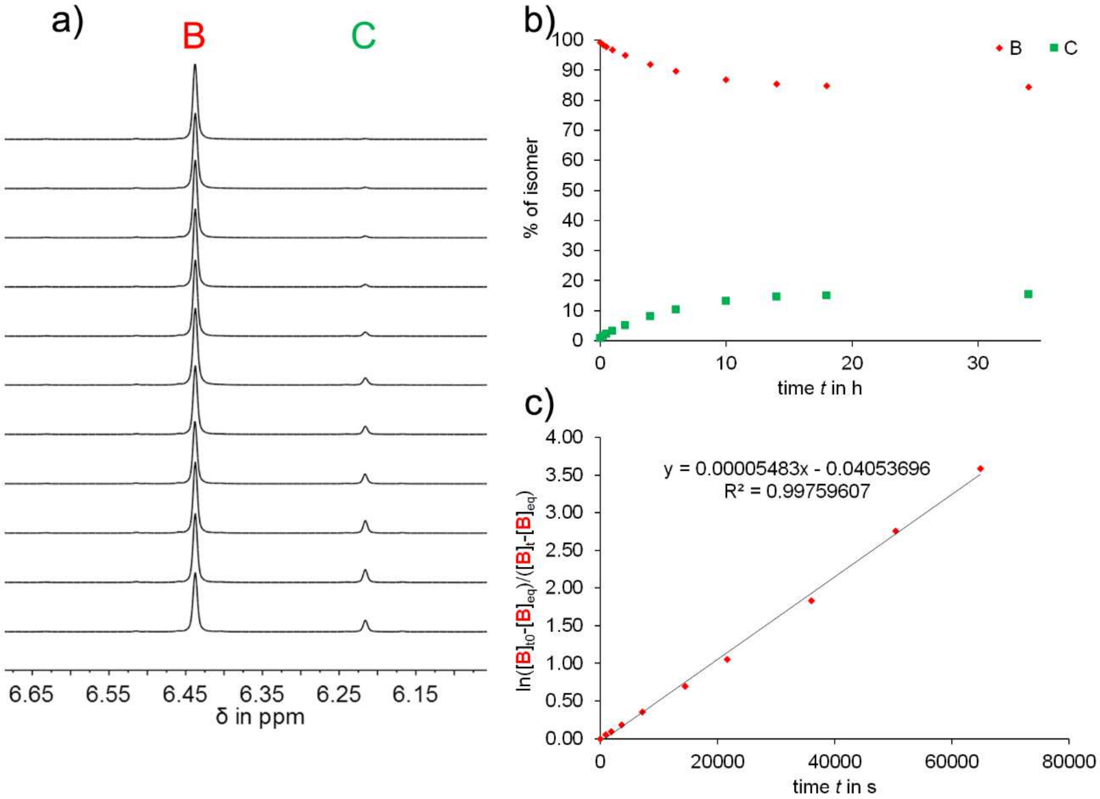

Supplementary Figure 8 | Thermal isomerization from B to C in the dark. a) Thermal isomerization of **B** to **C** in DMSO-d_6 at 80 °C followed by ^{1}H NMR spectroscopy (400 MHz, 20 °C) in regular time intervals. b) Thermal isomer conversion from **B** to **C**. c) First order kinetic analysis of the thermal isomerization of **B** to **C**. Taking into account the dynamic equilibrium by plotting according to Supplementary Equation 1 gives a linear relationship. The slope m can be translated into the rate constant $k_{(\mathbf{B/C})}$ according to Supplementary Equation 2. The corresponding Gibbs free energy of activation ΔG^* for the thermal **B** to **C** isomerization is given in Supplementary Table 2.

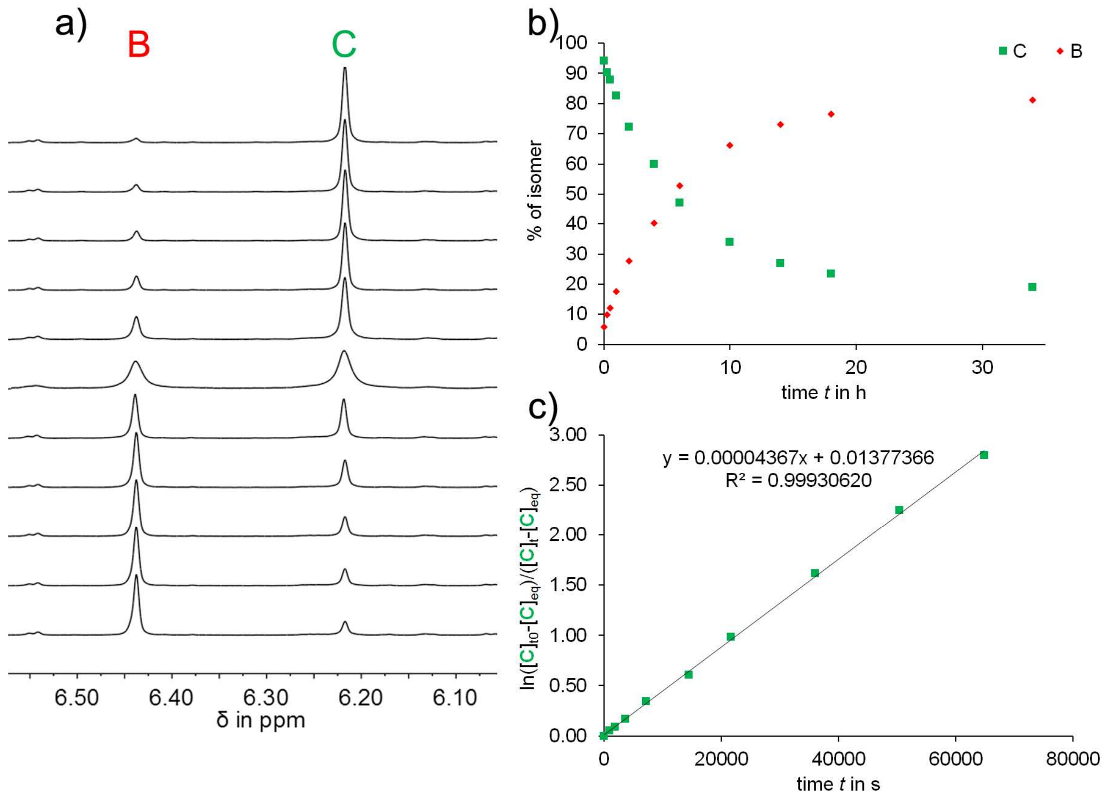

Supplementary Figure 9 | Thermal isomerization from C to B in the dark. a) Thermal isomerization of **C** to **B** in DMSO-d_6 at 80 °C followed by ^{1}H NMR spectroscopy (400 MHz, 20 °C) in regular time intervals. b) Thermal isomer conversion from **C** to **B**. c) First order kinetic analysis of the thermal isomerization of **C** to **B**. Taking into account the dynamic equilibrium by plotting according to Supplementary Equation 1 gives a linear relationship. The slope m can be translated into the rate constant $k_{(\mathbf{C/B})}$ according to Supplementary Equation 2. The corresponding Gibbs free energy of activation ΔG^* for the thermal **C** to **B** isomerization is given in Supplementary Table 2.

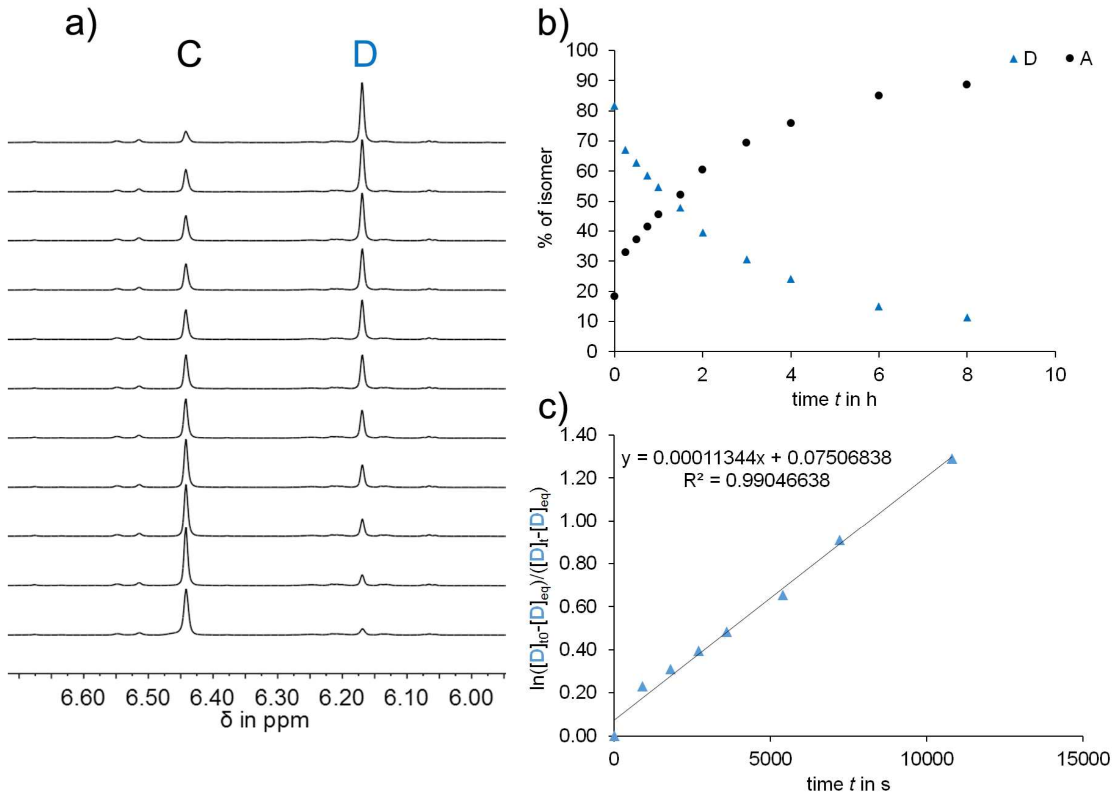

Supplementary Figure 10 | Thermal isomerization from D to A in the dark. a) Thermal isomerization of **D** to **A** in DMSO-d_6 at 60 °C followed by ^{1}H NMR spectroscopy (400 MHz, 20 °C) in regular time intervals. b) Thermal isomer conversion from **D** to **A**. c) First order kinetic analysis of the thermal isomerization of **D** to **A**. Taking into account the dynamic equilibrium by plotting according to Supplementary Equation 1 gives a linear relationship. The slope m can be translated into the rate constant $k_{(\mathbf{D/A})}$ according to Supplementary Equation 2. The corresponding Gibbs free energy of activation ΔG^* for the thermal **D** to **A** isomerization is given in Supplementary Table 2.

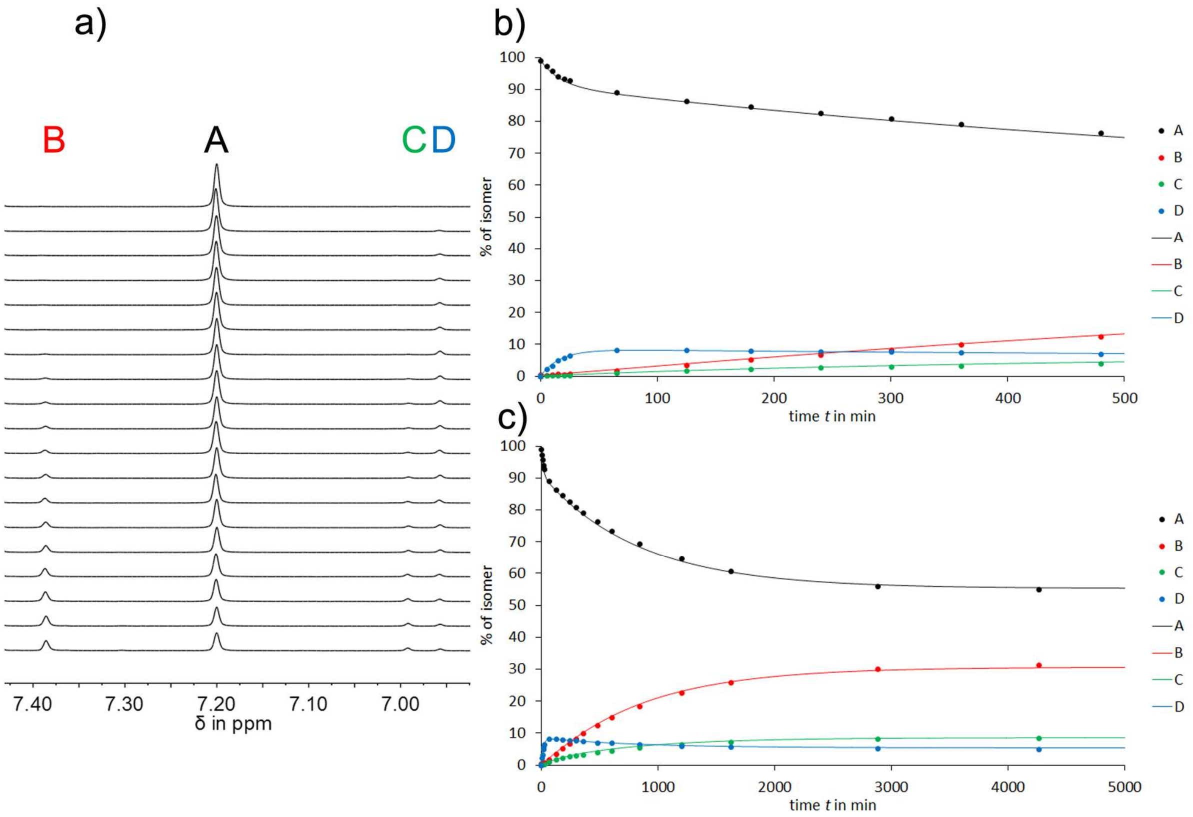

Supplementary Figure 11 | Thermal isomerization of A in 12DCB in the dark. a) Thermal isomerization of **A** in 12DCB-d_4 at 130 °C followed by ^{1}H NMR spectroscopy (400 MHz, 20 °C) in regular time intervals. b) Isomer conversion at the start of the thermal reaction of **A**. c) Isomer conversion over the whole thermal reaction of **A**. The corresponding Gibbs free energies of activation ΔG^* for the thermal reactions of **A** were obtained by fitting the experimental data according to Supplementary Equation 6. A refinement factor of 0.98 was obtained for the best fit. The values are given in Supplementary Table 3.

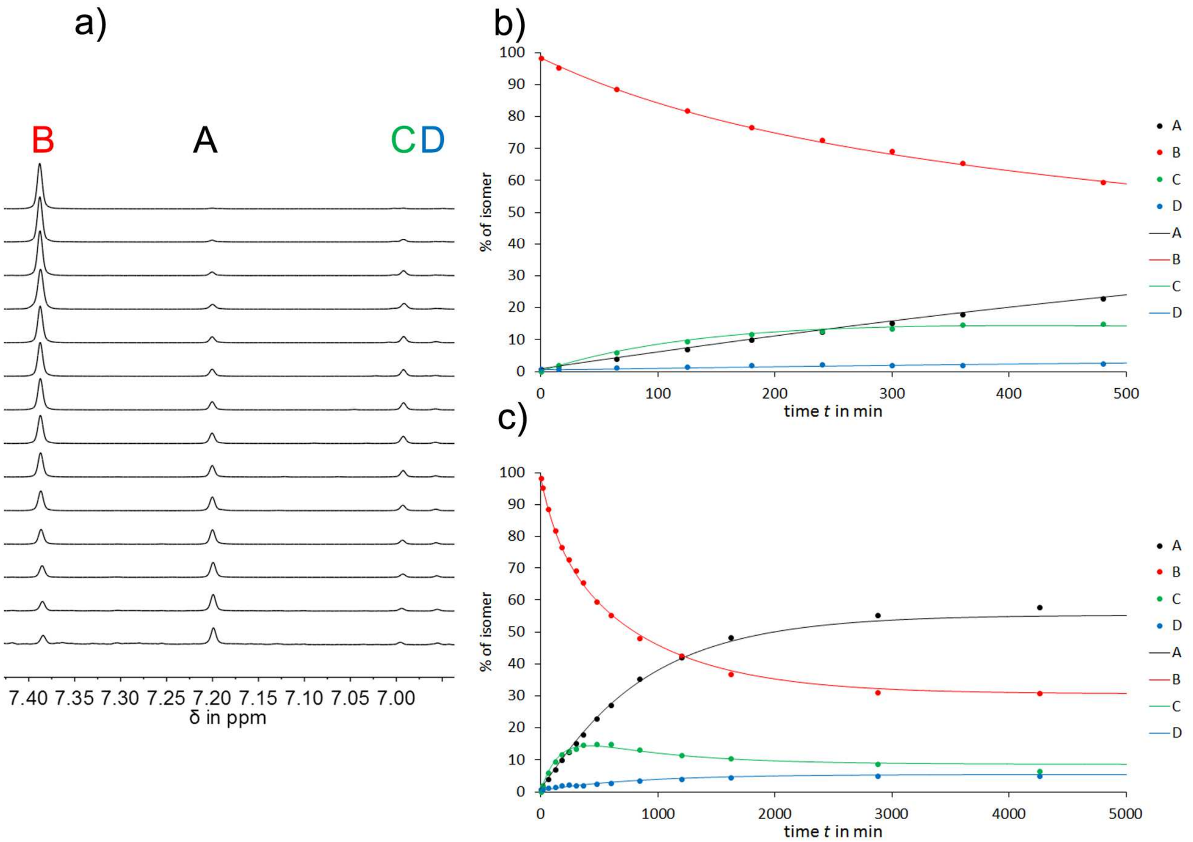

Supplementary Figure 12 | Thermal isomerization of B in 12DCB in the dark. a) Thermal isomerization of **B** in 12DCB -d_4 at 130 °C followed by ^{1}H NMR spectroscopy (400 MHz, 20 °C) in regular time intervals. b) Isomer conversion at the start of the thermal reaction of **B**. c) Isomer conversion over the whole thermal reaction of **B**. The corresponding Gibbs free energies of activation ΔG^* for the thermal reactions of **B** were obtained by fitting the experimental data according to Supplementary Equation 6. A refinement factor of 0.98 was obtained for the best fit. The values are given in Supplementary Table 3.

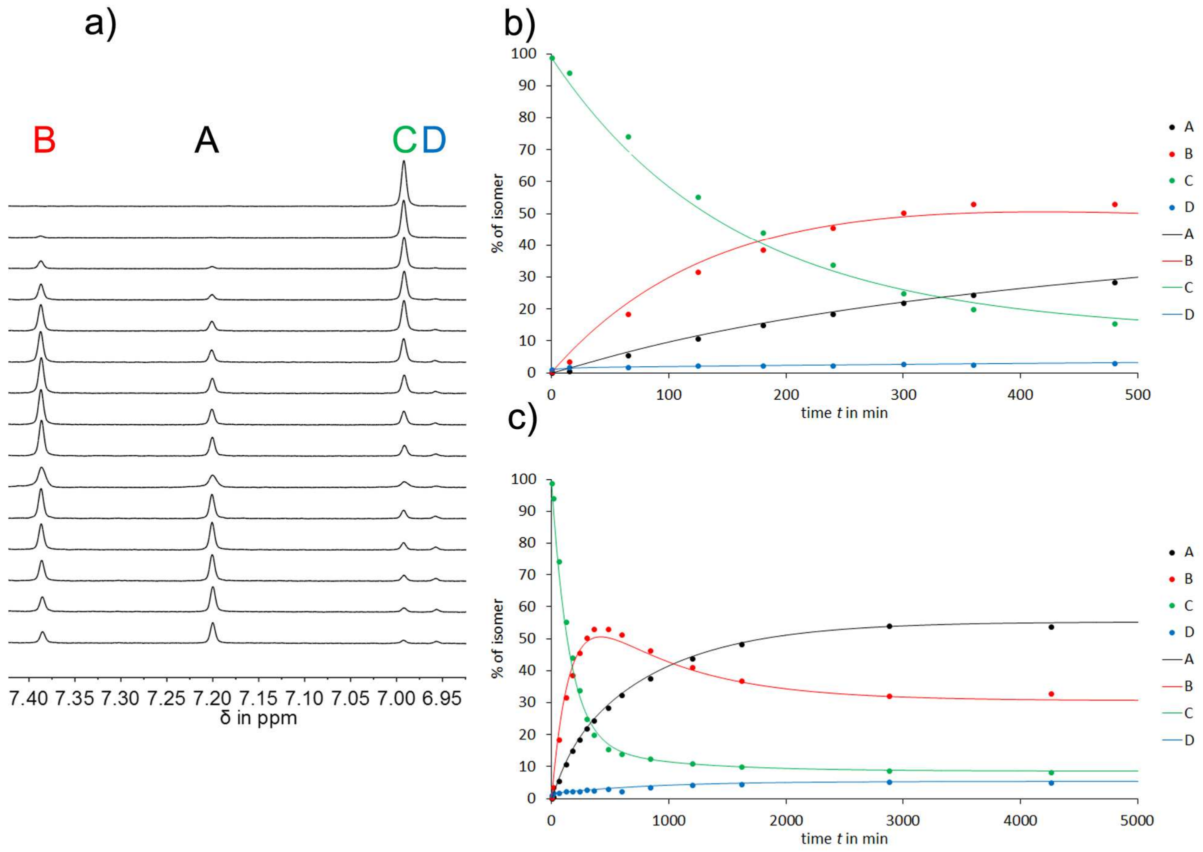

Supplementary Figure 13 | Thermal isomerization of C in 12DCB. a) Thermal isomerization of **C** in 12DCB-d_4 at 130 °C followed by ^{1}H NMR spectroscopy (400 MHz, 20 °C) in regular time intervals. b) Isomer conversion at the start of the thermal reaction of **C**. c) Isomer conversion over the whole thermal reaction of **C**. The corresponding Gibbs free energies of activation ΔG^* for the thermal reactions of **C** were obtained by fitting the experimental data according to Supplementary Equation 6. A refinement factor of 0.98 was obtained for the best fit. The values are given in Supplementary Table 3.

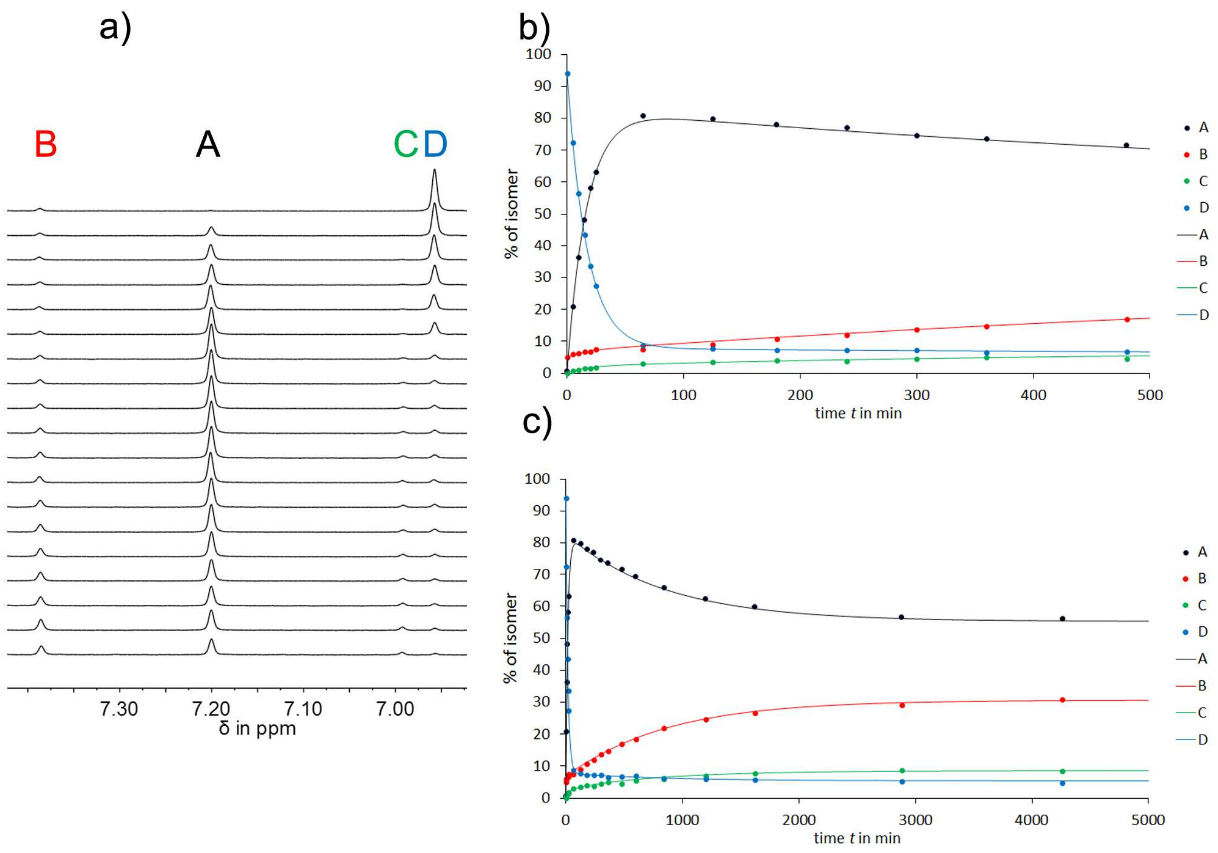

Supplementary Figure 14 | Thermal isomerization of D in 12DCB in the dark. a) Thermal isomerization of **D** in ODCB-d_4 at 130 °C followed by ^{1}H NMR spectroscopy (400 MHz, 20 °C) in regular time intervals. b) Isomer conversion at the start of the thermal reaction of **D**. c) Isomer conversion over the whole thermal reaction of **D**. The corresponding Gibbs free energies of activation ΔG^* for the thermal reactions of **A** were obtained by fitting the experimental data according to Supplementary Equation 6. A refinement factor of 0.98 was obtained for the best fit. The values are given in Supplementary Table 3.

Molar absorption coefficients

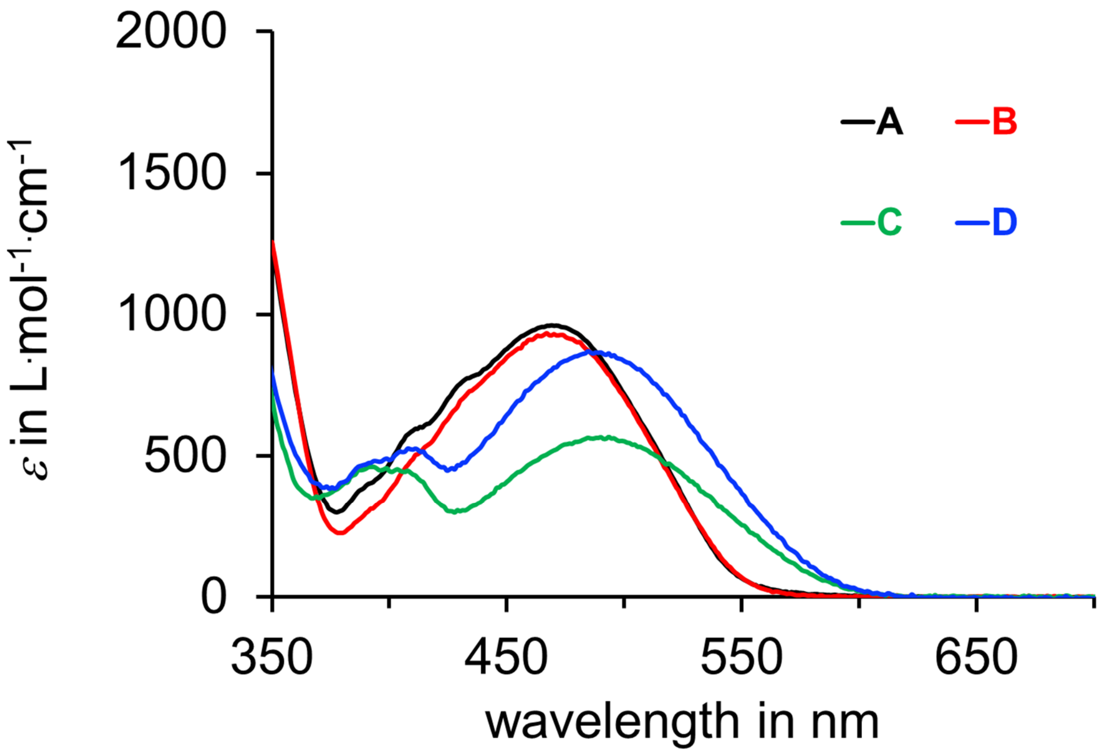

Supplementary Figure 15 | Comparison of molar absorption coefficients of all four isomers of 1 in *c*hexane.

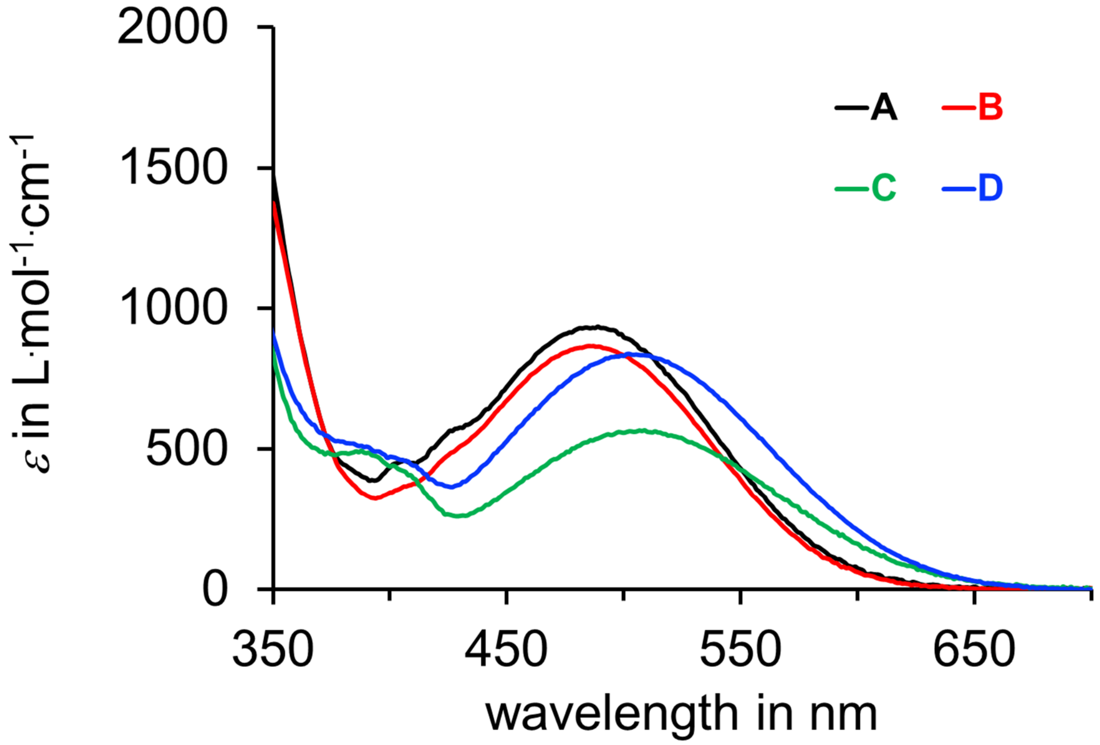

Supplementary Figure 16 | Comparison of molar absorption coefficients of all four isomers of 1 in 12DCB.

Fluorescence spectra in *c*hexane

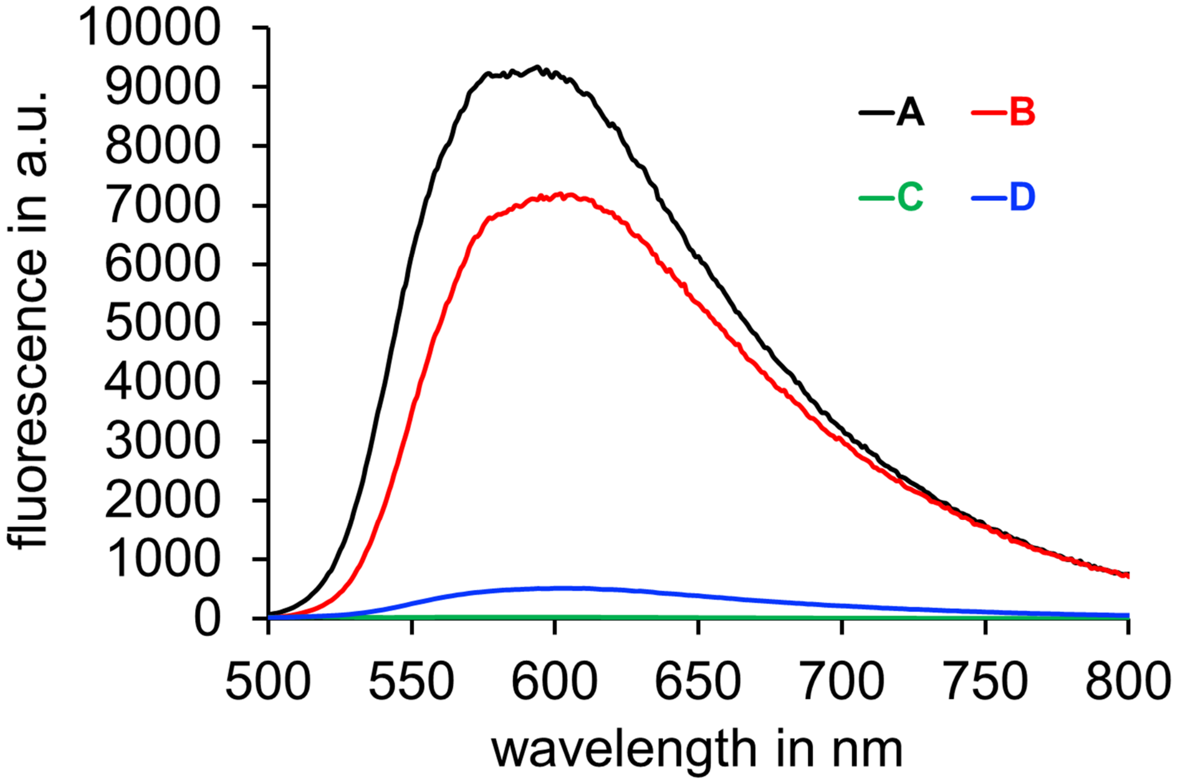

Supplementary Figure 17 | Fluorescence spectra (excitation with 450 nm) of 1 in *c*hexane. The concentration of **1-A** in *c*hexane was 0.26 mM. The fluorescence spectra in the other solvents were multiplied with a factor to account for different concentrations used and to enable quantitative comparison of the spectra with the spectrum of **1-A**.

Quantum yield measurements in different solvents

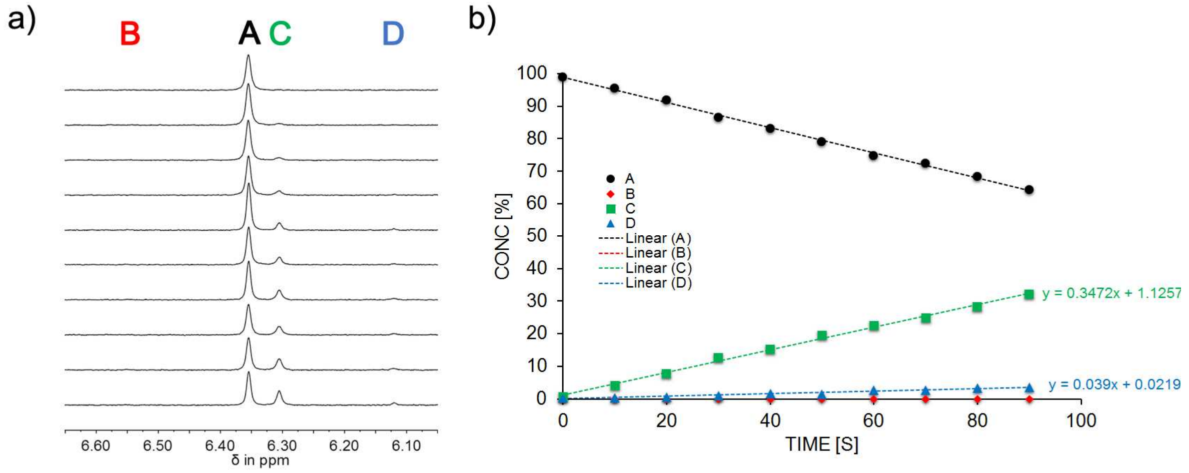

Supplementary Figure 18 | Quantum yields of the photoreactions of A in *c*hexane solution. Quantum yield ϕ measurement for the photoconversion of **A-1** at 25 °C in *c*hexane solution (1.01 mM) using a focused 520 nm LED. a) ^{1}H NMR spectra (400 MHz, 18 °C, *c*hexane was removed *in vacuo* and replaced with $CDCl_3$ prior to NMR measurements) recorded after different irradiation durations. Signals of individual isomers are assigned. b) Relative changes of the isomer composition plotted against different duration times of irradiation. Each point represents an individual measurement, the relative isomer ratios were determined by ^{1}H NMR spectroscopy. Quantum yields were determined by averaging over all experiments and are given in Supplementary Table 4.

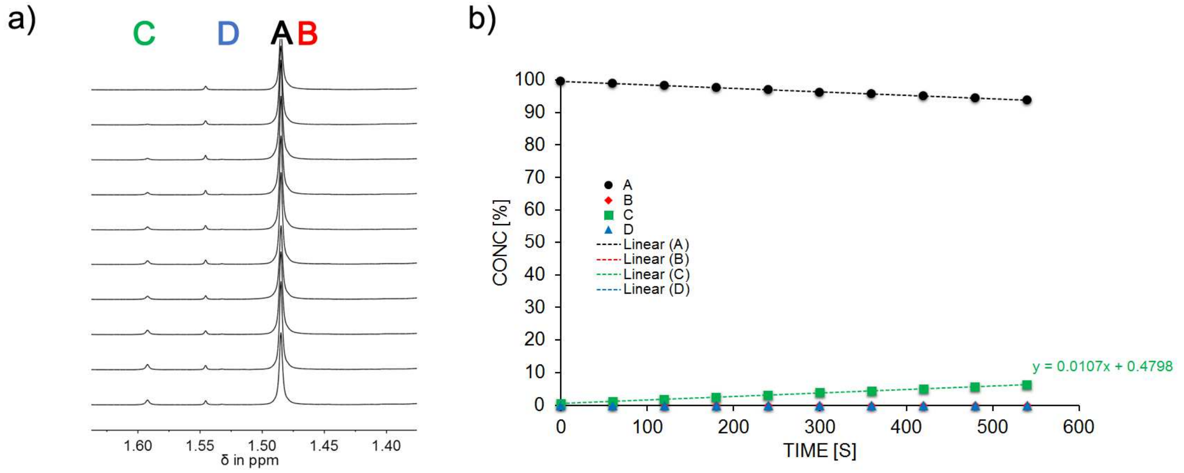

Supplementary Figure 19 | Quantum yields of the photoreactions of A in 12DCB solution. Quantum yield ϕ measurement for the photoconversion of **A-1** at 25 °C in 12DCB solution (0.97 mM) using a focused 520 nm LED. a) ^{1}H NMR spectra (400 MHz, 18 °C, 12DCB was removed *in vacuo* and replaced with benzene-d_6 prior to NMR measurements) recorded after different irradiation durations. Signals of individual isomers are assigned. b) Relative changes of the isomer composition plotted against different duration times of irradiation. Each point represents an individual measurement, the relative isomer ratios were determined by ^{1}H NMR spectroscopy. Quantum yields were determined by averaging over all experiments and are given in Supplementary Table 4.

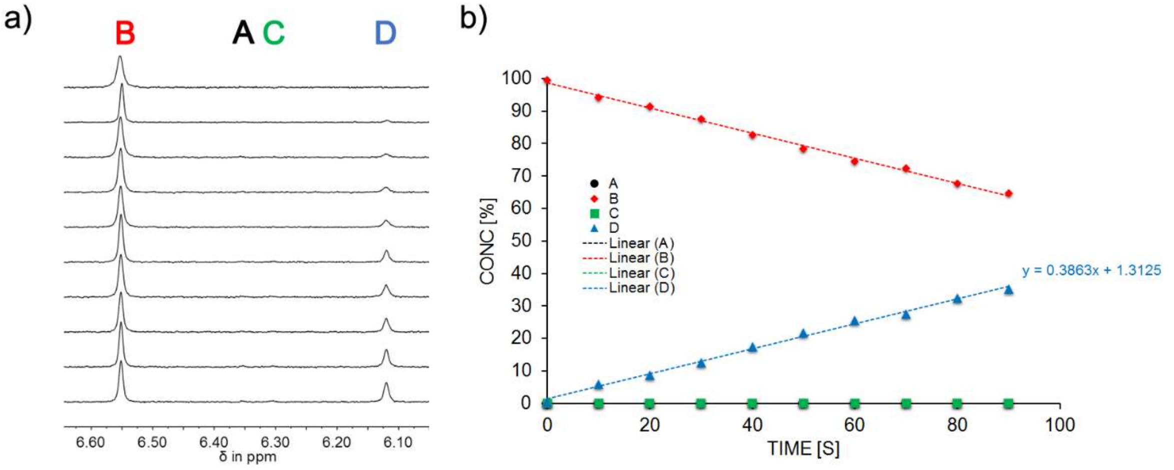

Supplementary Figure 20 | Quantum yields of the photoreactions of B in *c*hexane solution. Quantum yield ϕ measurement for the photoconversion of **B-1** at 25 °C in *c*hexane solution (0.87 mM) using a focused 520 nm LED. a) ^{1}H NMR spectra (400 MHz, 18 °C, *c*hexane was removed *in vacuo* and replaced with $CDCl_3$ prior to NMR measurements) recorded after different irradiation durations. Signals of individual isomers are assigned. b) Relative changes of the isomer composition plotted against different duration times of irradiation. Each point represents an individual measurement, the relative isomer ratios were determined by ^{1}H NMR spectroscopy. Quantum yields were determined by averaging over all experiments and are given in Supplementary Table 4.

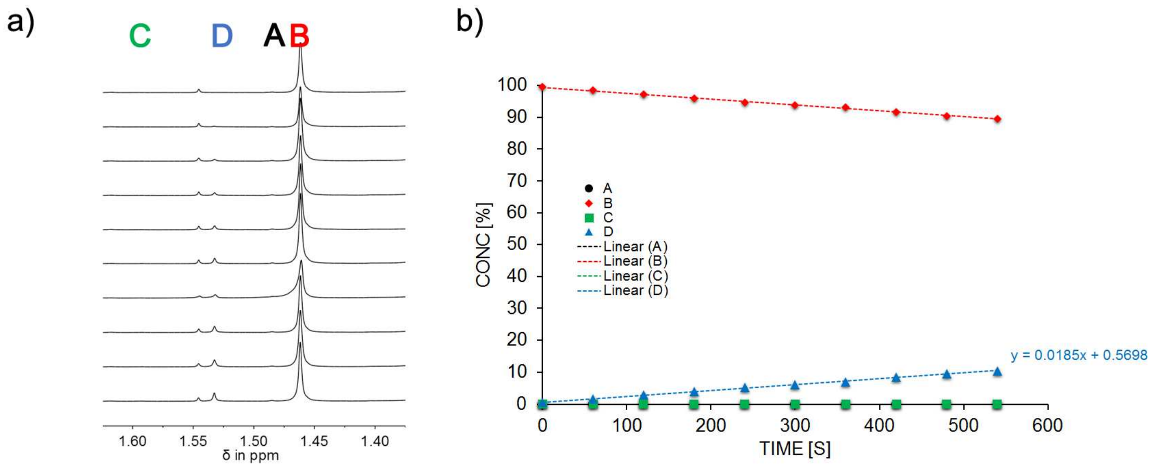

Supplementary Figure 21 | Quantum yields of the photoreactions of B in 12DCB solution. Quantum yield ϕ measurement for the photoconversion of **B-1** at 25 °C in 12DCB solution (0.97 mM) using a focused 520 nm LED. a) ^{1}H NMR spectra (400 MHz, 18 °C, 12DCB was removed *in vacuo* and replaced with benzene-d_6 prior to NMR measurements) recorded after different irradiation durations. Signals of individual isomers are assigned. b) Relative changes of the isomer composition plotted against different duration times of irradiation. Each point represents an individual measurement, the relative isomer ratios were determined by ^{1}H NMR spectroscopy. Quantum yields were determined by averaging over all experiments and are given in Supplementary Table 4.

Motor operation followed by NMR-spectroscopy

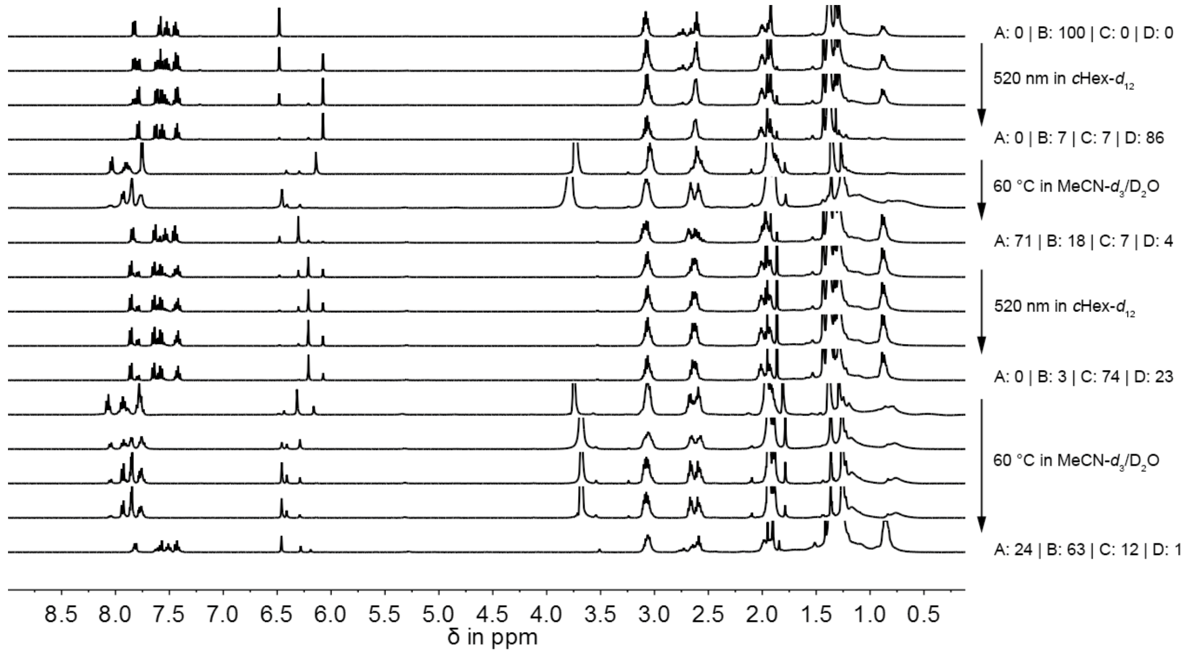

Supplementary Figure 22 | Motor operation of 1 followed by ¹H NMR spectroscopy (400 MHz, 18 °C) in different solvents. All steps were recorded for a single sample in a row starting with pure isomer **B**. Solvent changes were done by removing the current solvent *in vacuo* and replacing it with a new one.

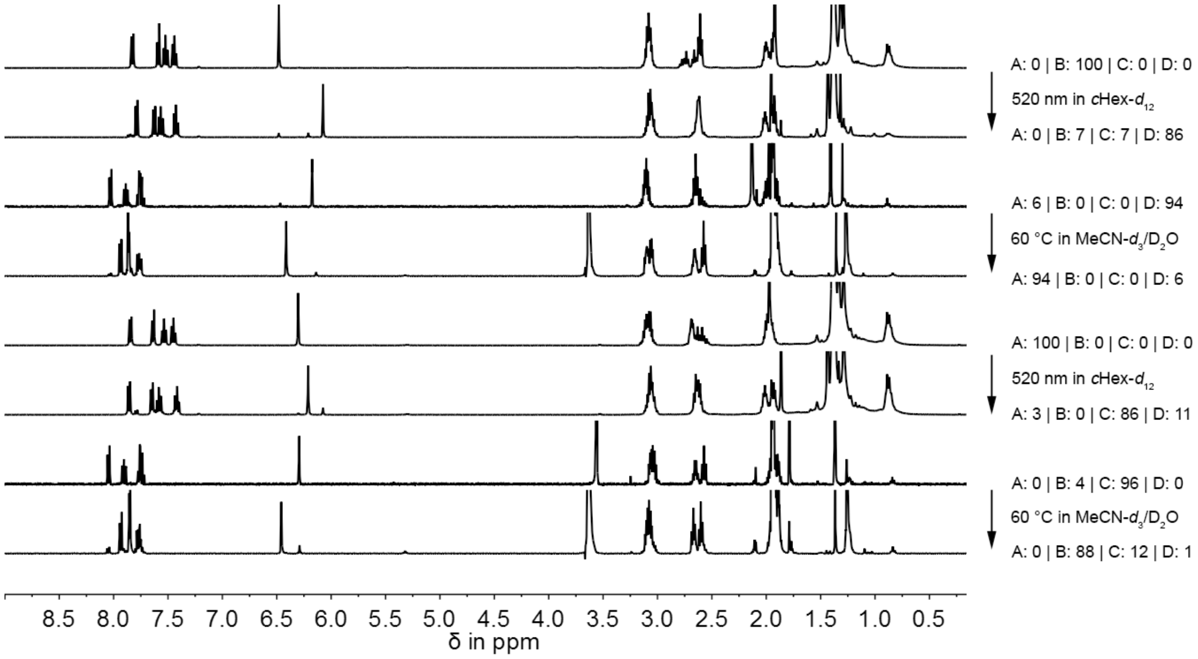

Supplementary Figure 23 | Motor operation of 1 followed by ¹H NMR spectroscopy (400 MHz, 18 °C) in different solvents separately for each transformation. A new solution of each respective pure isomer in the respective solvent was used for each step allowing to quantify the corresponding selectivity and degree of completeness individually.

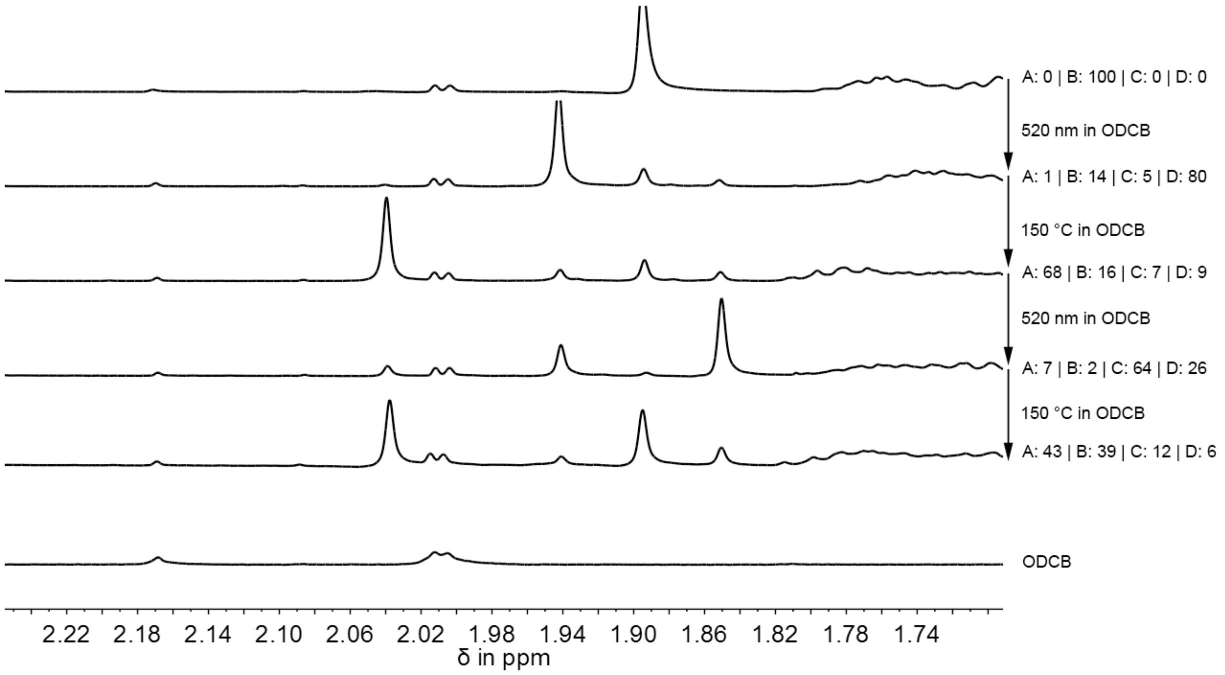

Supplementary Figure 24 | Motor operation of 1 followed by ^{1}H NMR spectroscopy (400 MHz, 18 °C) in non-deuterated 12DCB. All steps were recorded for a single sample in a row starting with pure isomer **B**.

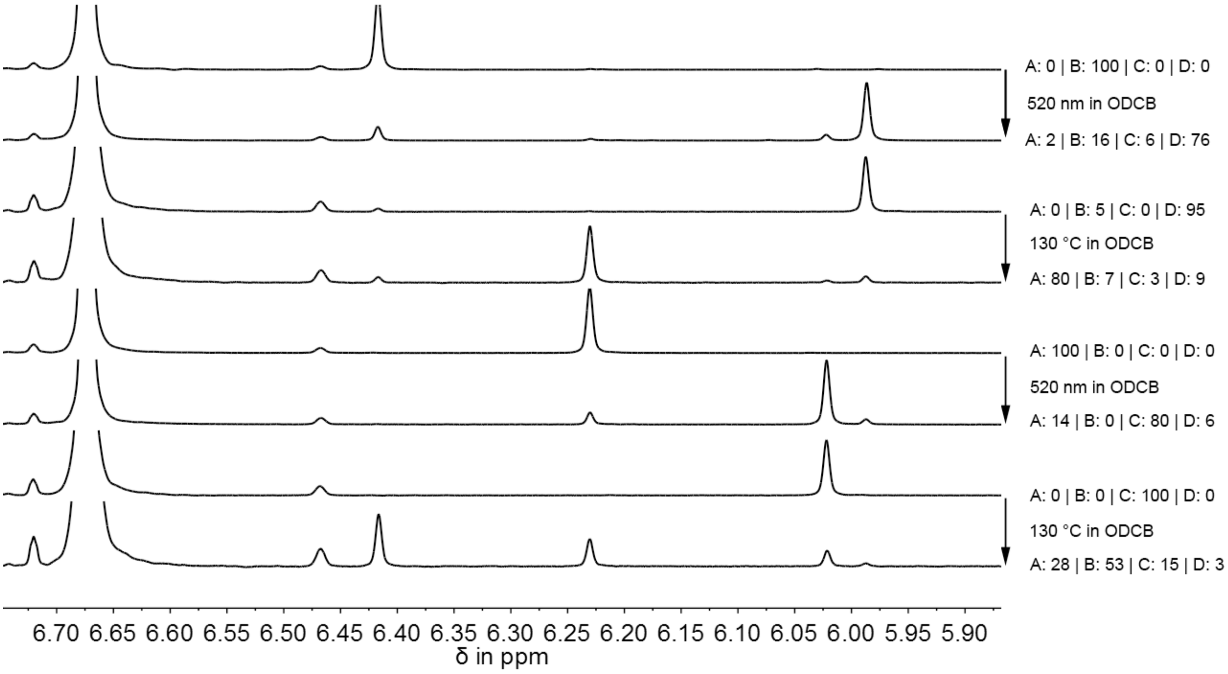

Supplementary Figure 25 | Motor operation of 1 followed by ^{1}H NMR spectroscopy (400 MHz, 18 °C) in 12DCB-*d*$_4$ separately for each transformation. A new solution of each respective pure isomer was used for each step allowing to quantify the corresponding selectivity and degree of completeness individually.

Theoretical description of the ground state of 1

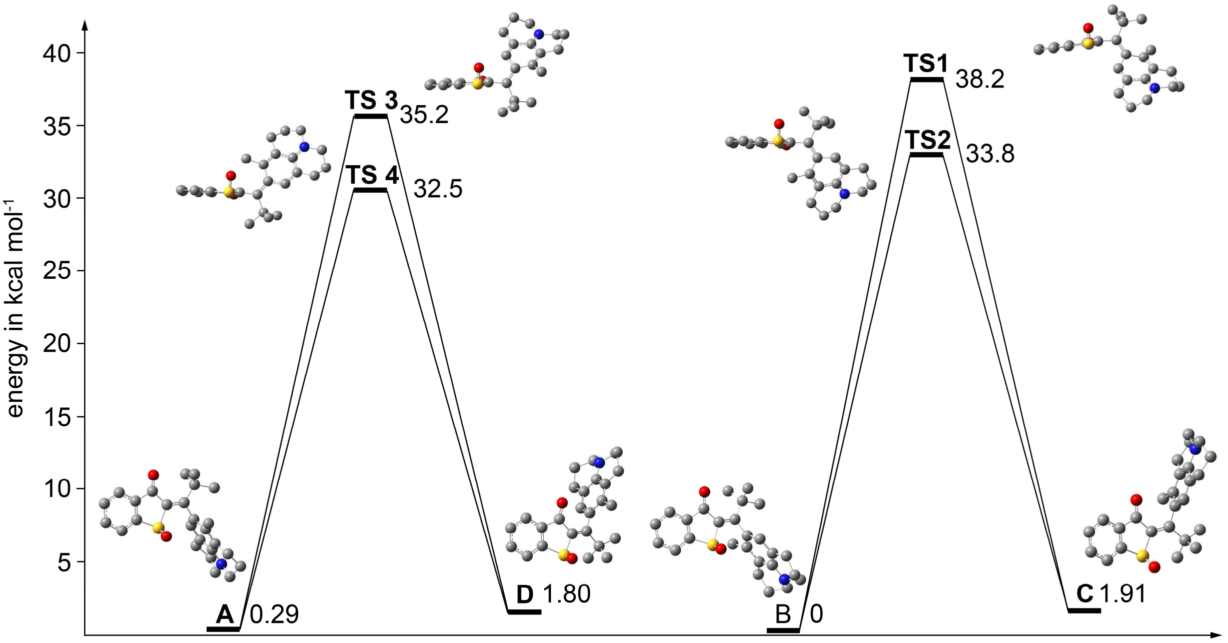

Supplementary Figure 26 | Calculated ground state energy profile of 1 in DMSO solution. All energy values are given in kcal mol^{-1} and were obtained by DFT calculations at the ω–B97XD/6-311G(d,p) level of theory.

NMR Spectra

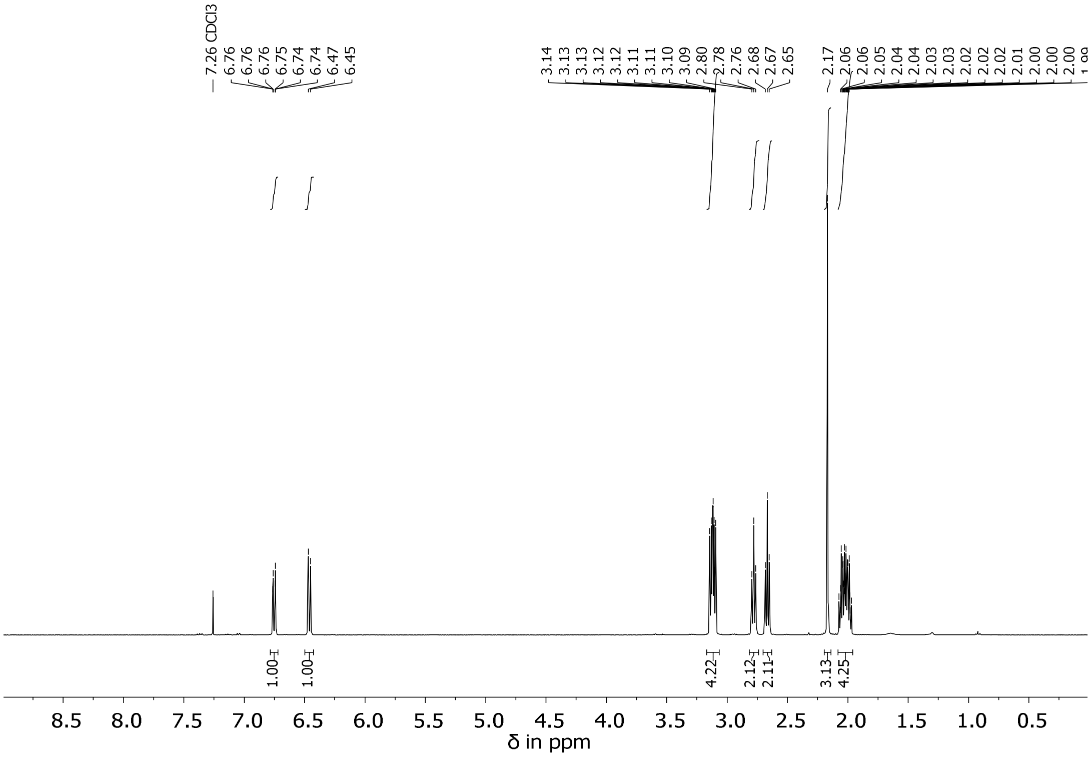

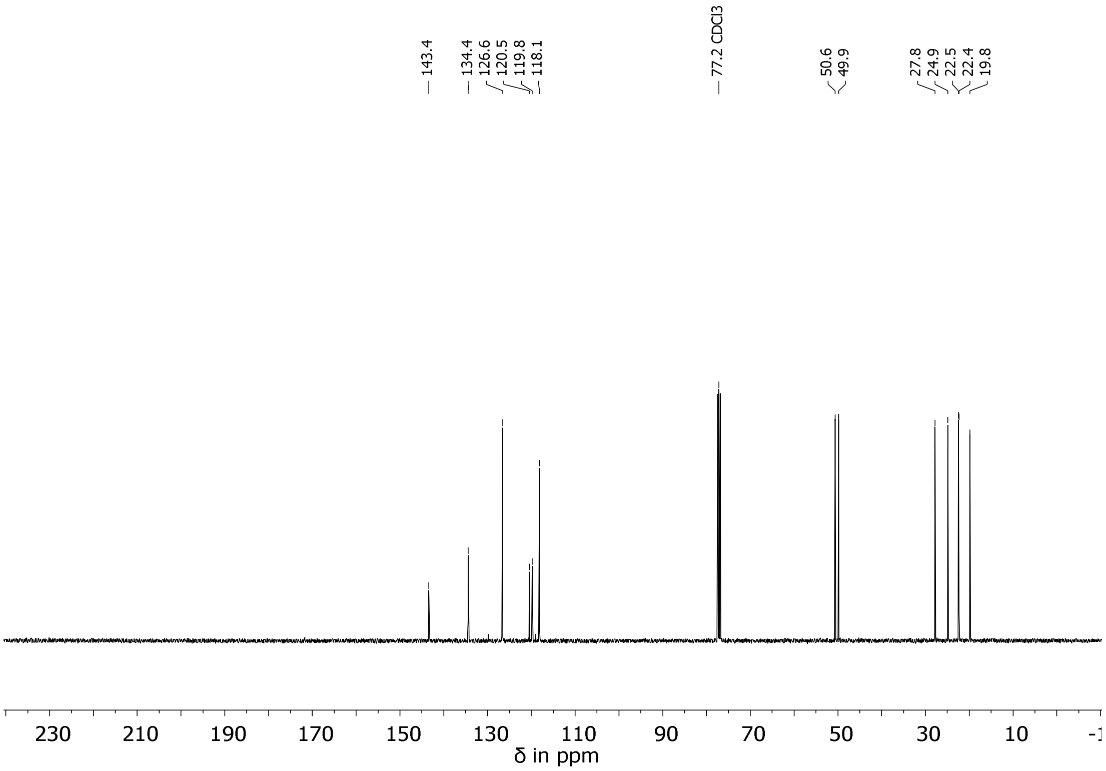

Supplementary Figure 27 | 400 MHz ^{1}H NMR spectrum (top) and 100 MHz ^{13}C NMR spectrum (bottom) of 4 in $CDCl_3$.

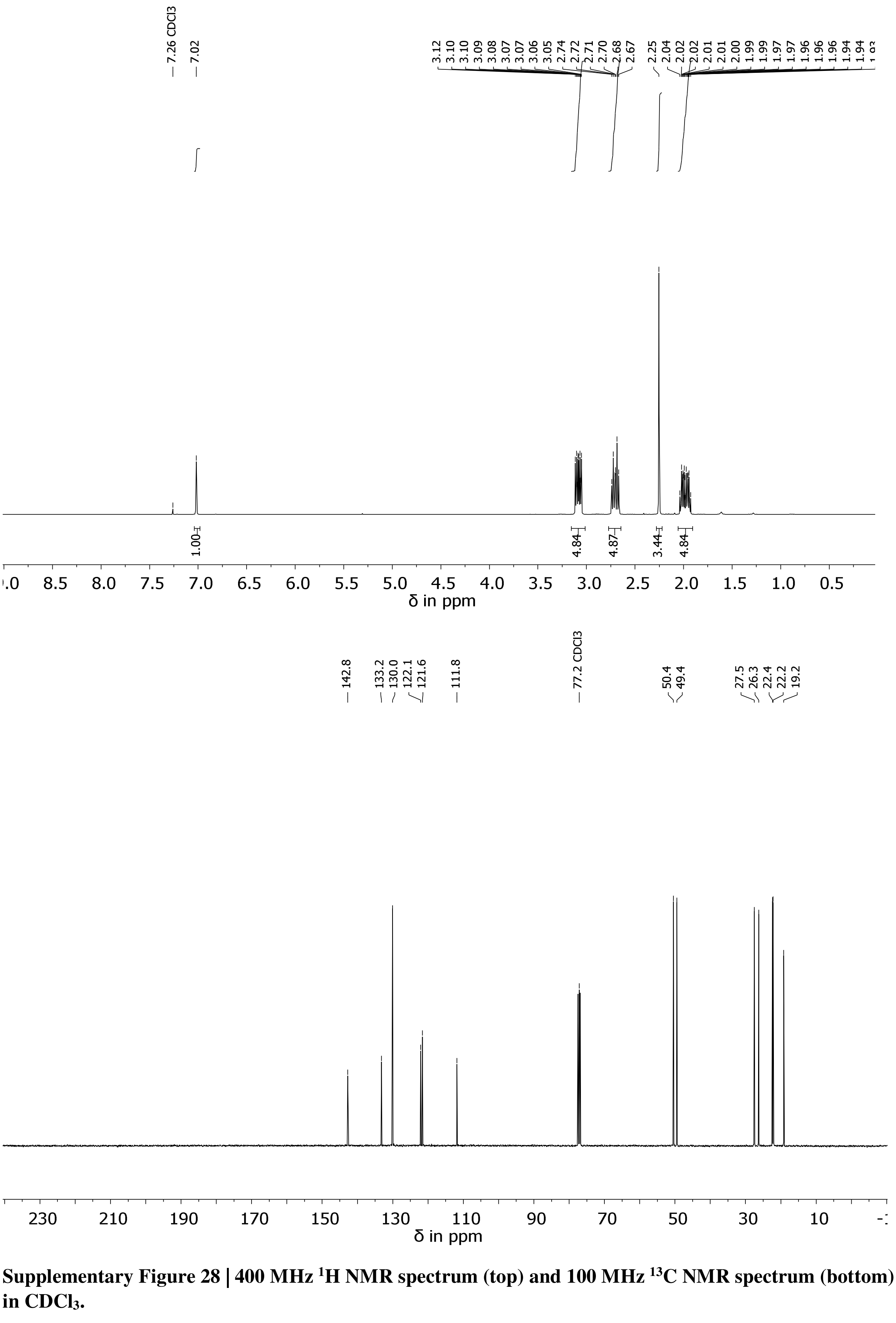

Supplementary Figure 28 | 400 MHz ^{1}H NMR spectrum (top) and 100 MHz ^{13}C NMR spectrum (bottom) of 5 in $CDCl_3$.

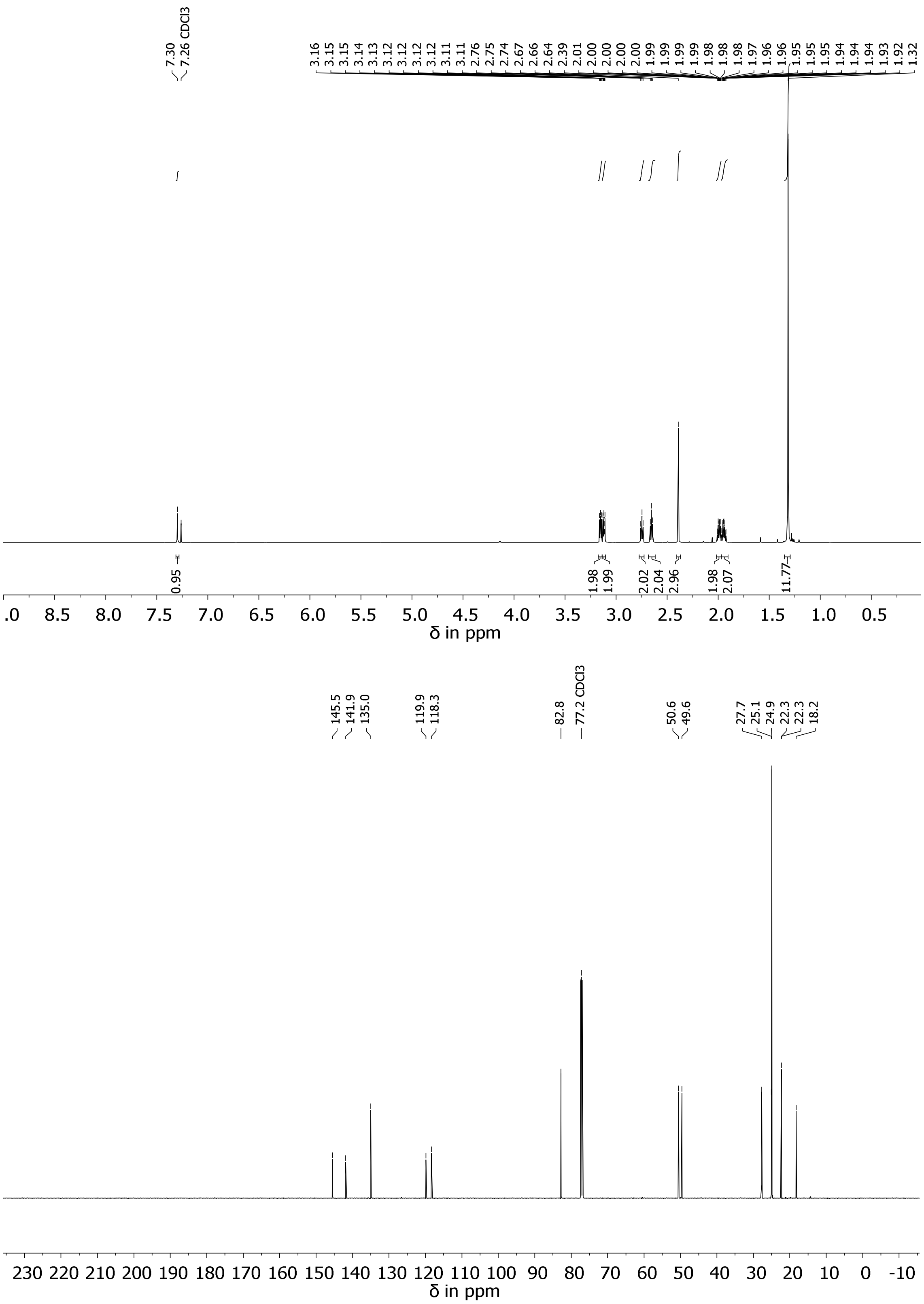

Supplementary Figure 29 | 400 MHz ^{1}H NMR spectrum (top) and 100 MHz ^{13}C NMR spectrum (bottom) of 6 in $CDCl_3$.

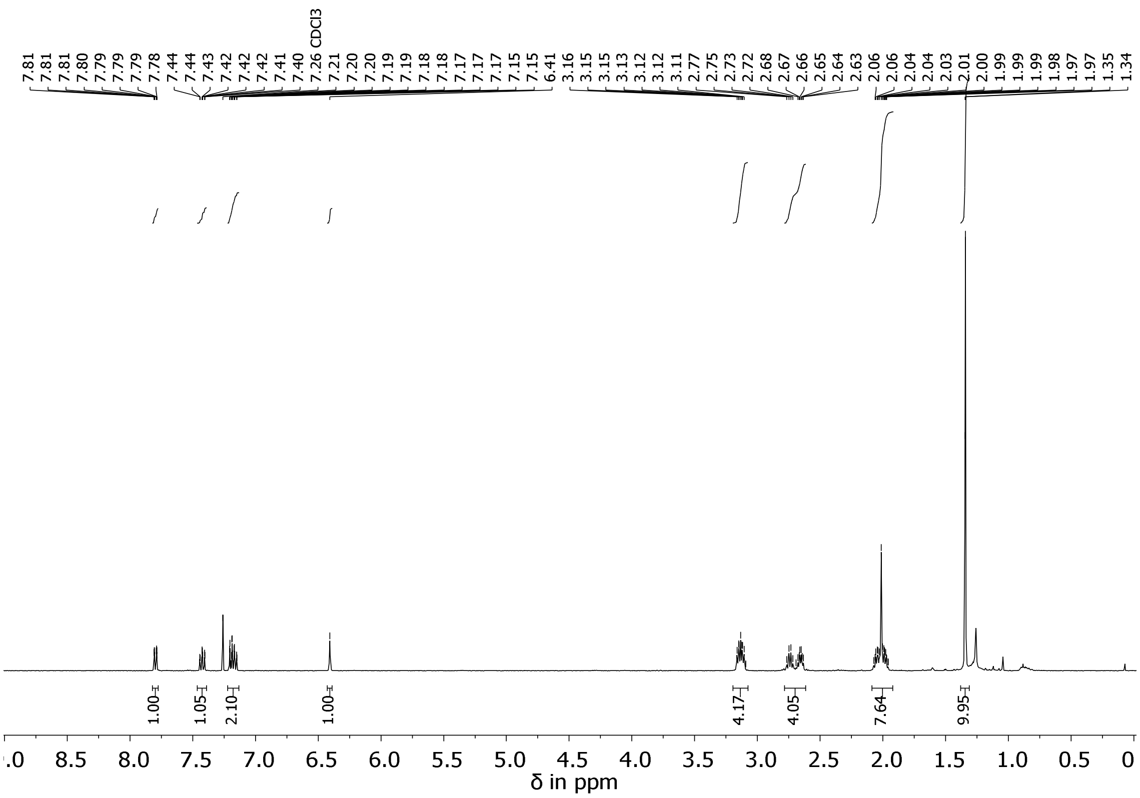

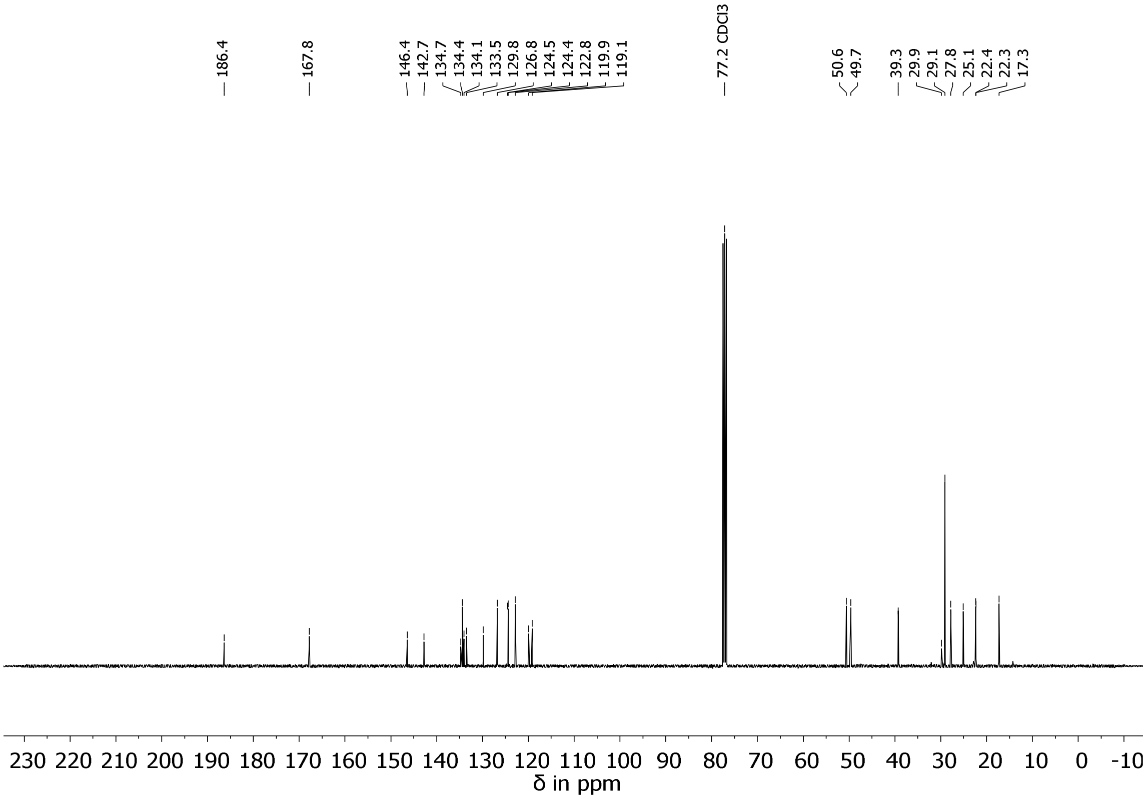

Supplementary Figure 30 | 400 MHz ^{1}H NMR spectrum (top) and 100 MHz ^{13}C NMR spectrum (bottom) of 7 in $CDCl_3$.

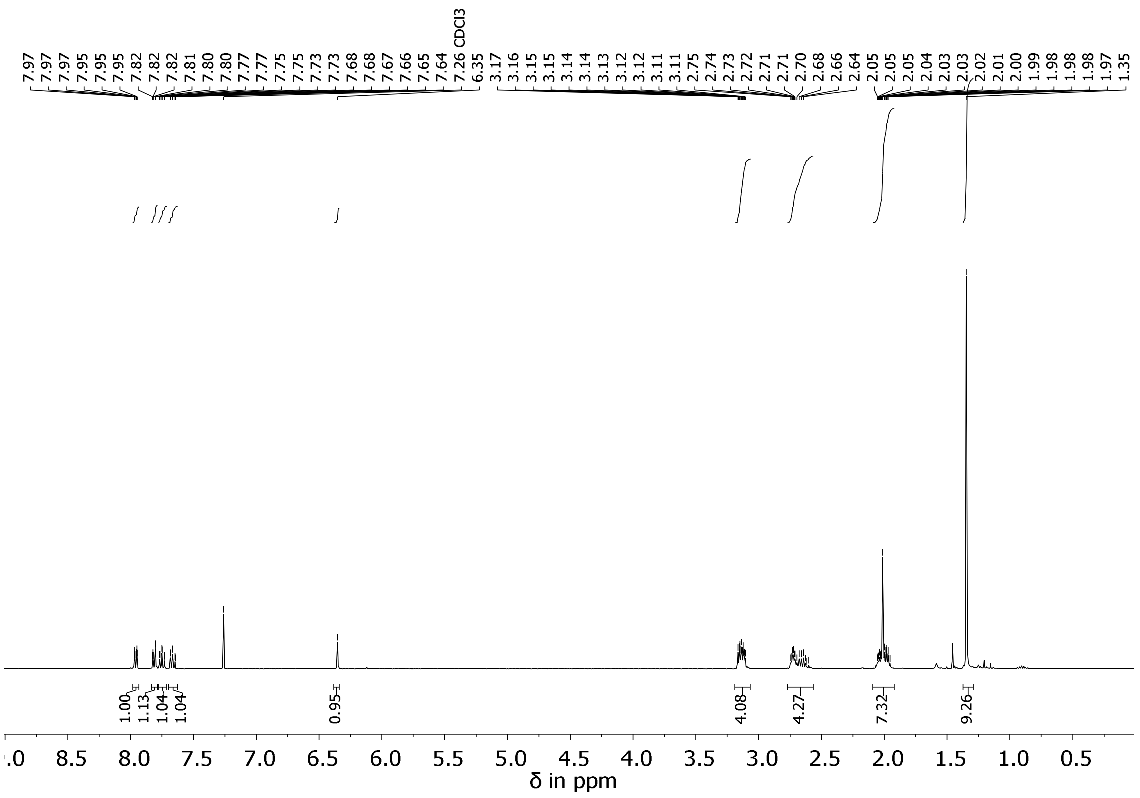

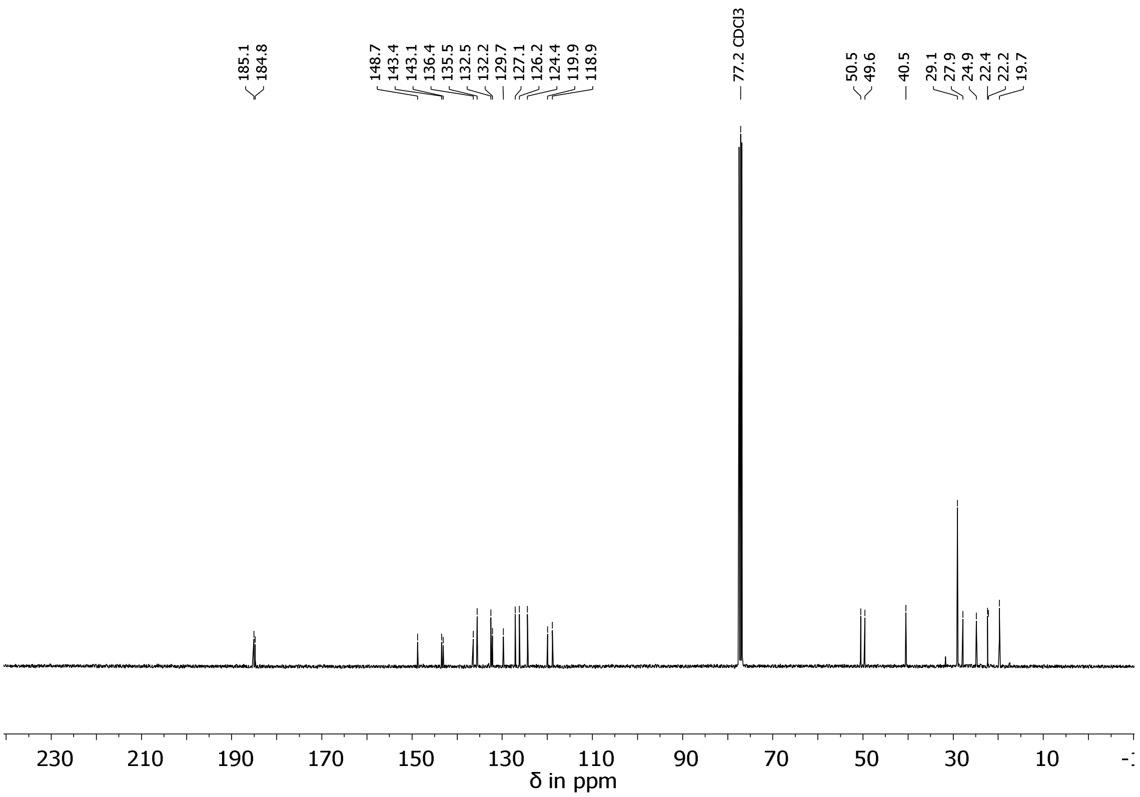

Supplementary Figure 31 | 400 MHz ^{1}H NMR spectrum (top) and 100 MHz ^{13}C NMR spectrum (bottom) of A-1 in $CDCl_3$.

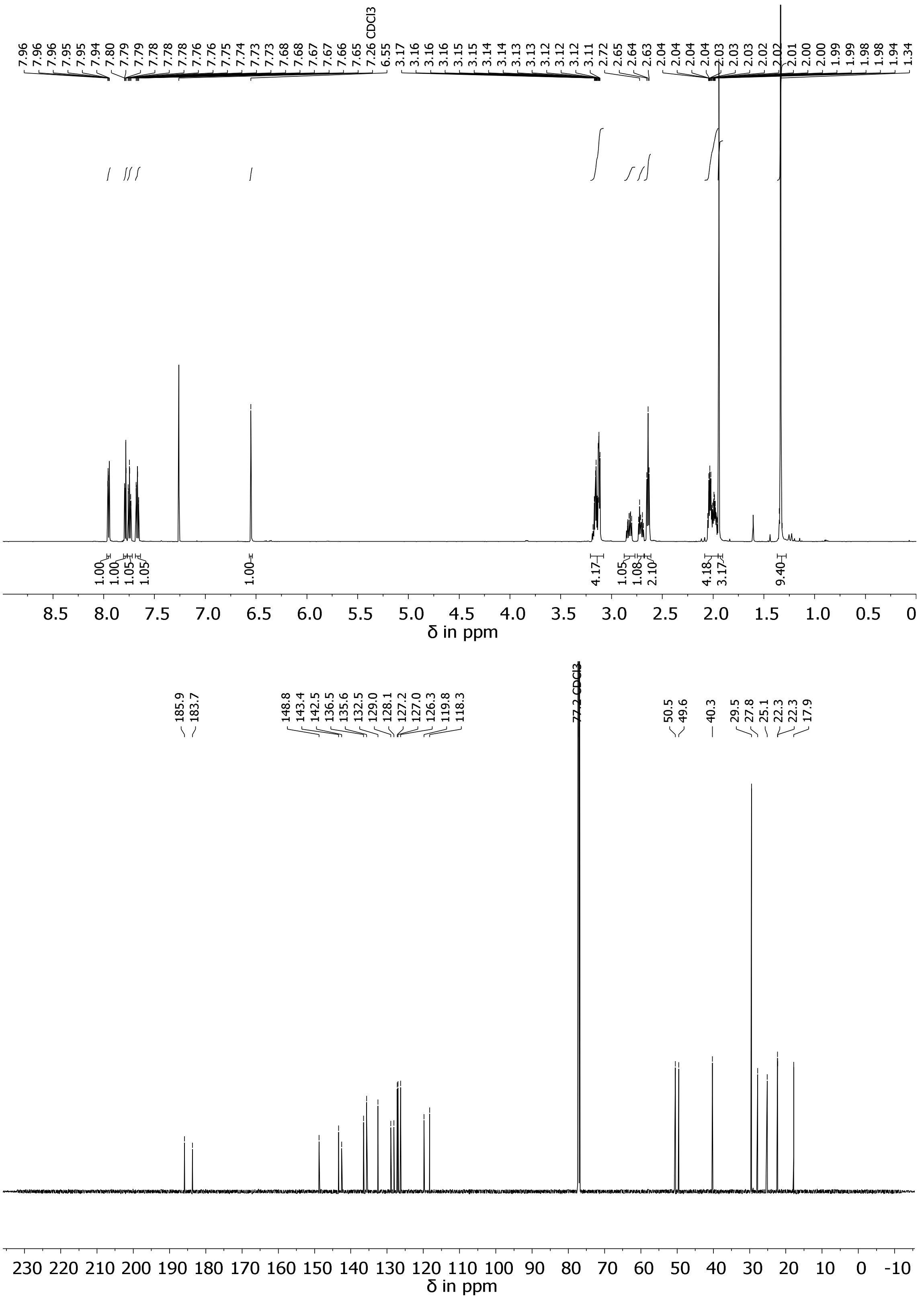

Supplementary Figure 32 | 599 MHz ^{1}H NMR spectrum (top) and 151 MHz ^{13}C NMR spectrum (bottom) of B-1 in $CDCl_3$.

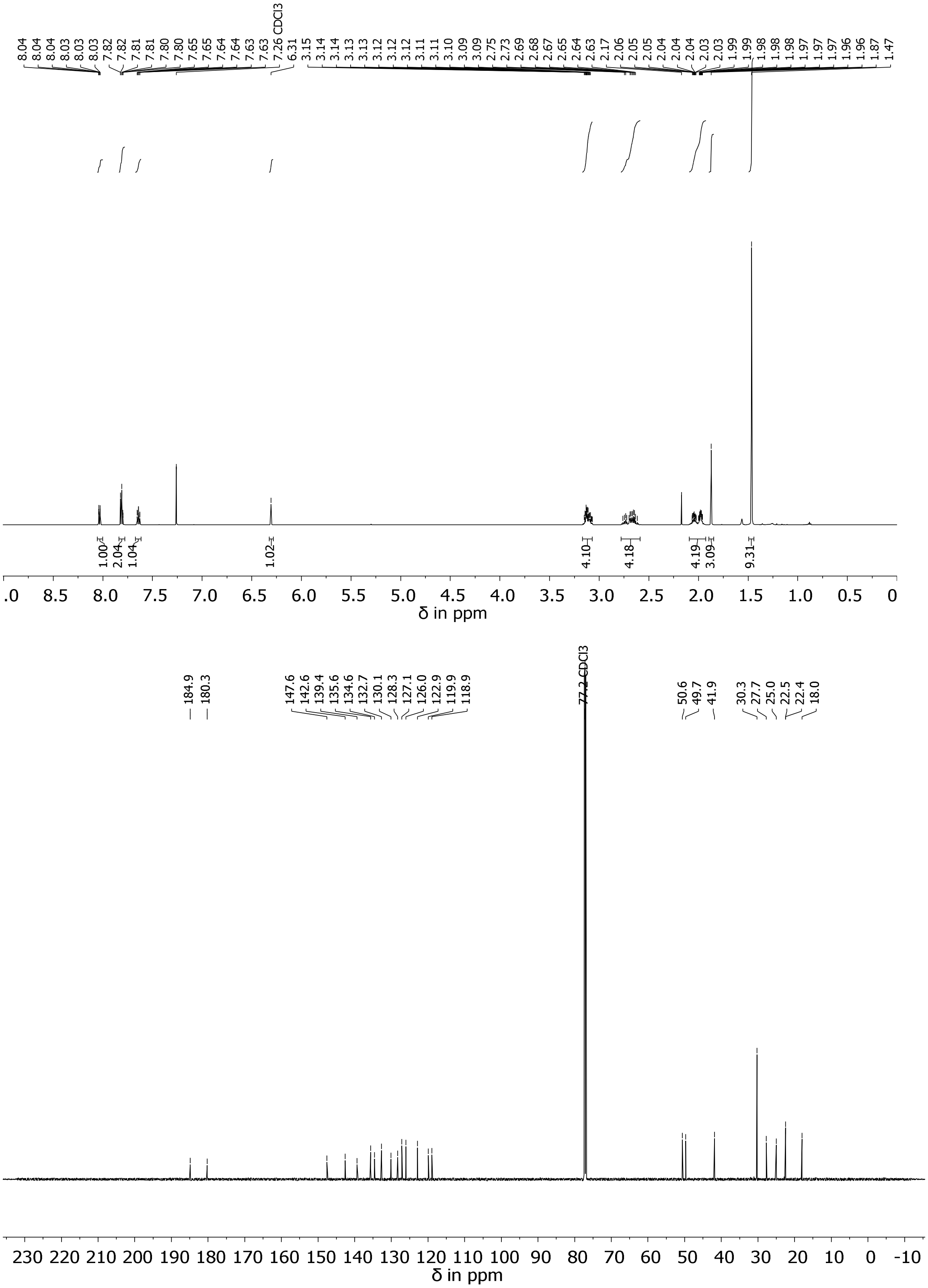

Supplementary Figure 33 | 599 MHz ^{1}H NMR spectrum (top) and 151 MHz ^{13}C NMR spectrum (bottom) of C-1 in $CDCl_3$.

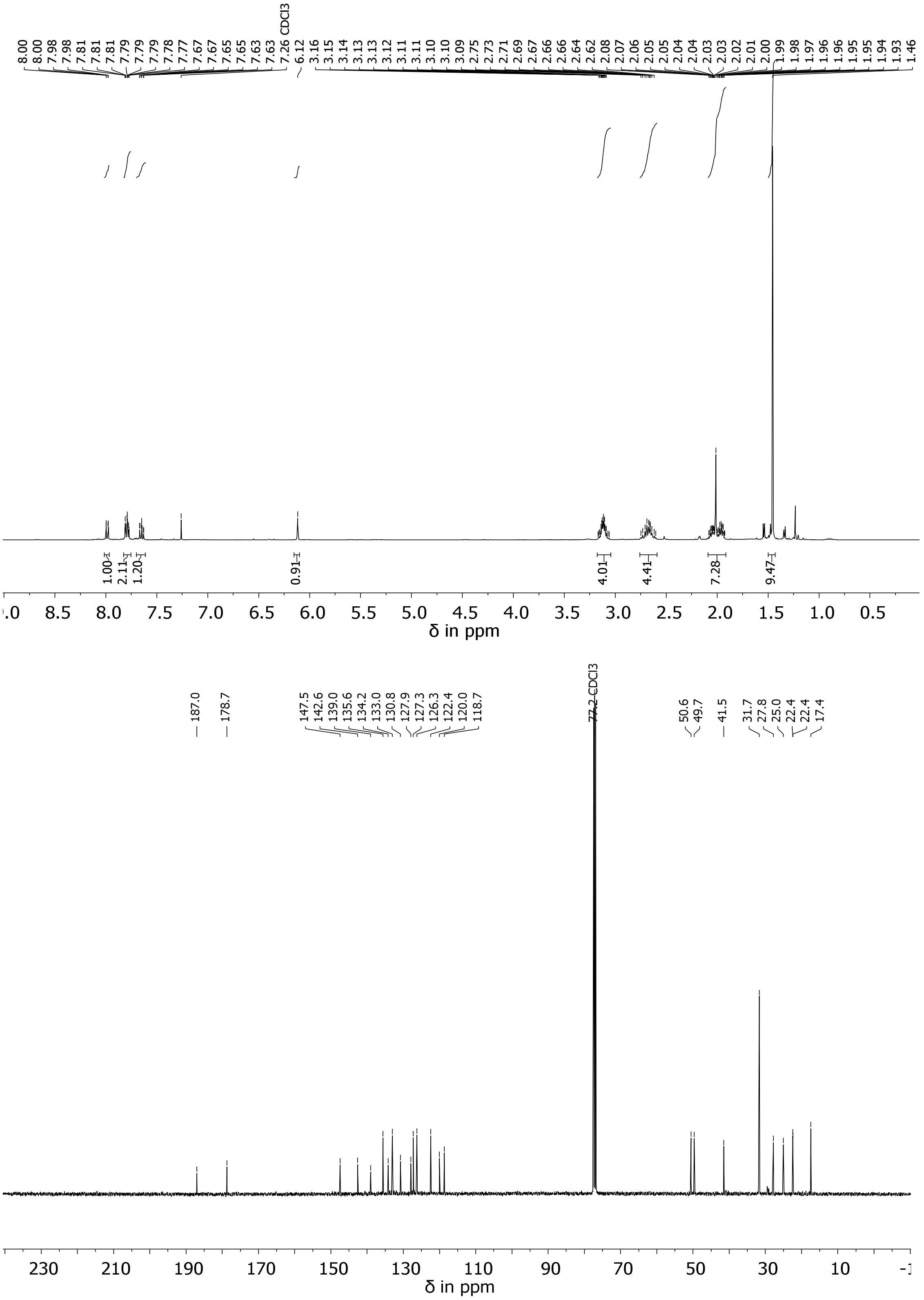

Supplementary Figure 34 | 400 MHz ^{1}H NMR spectrum (top) and 100 MHz ^{13}C NMR spectrum (bottom) of D-1 in $CDCl_3$.

Supplementary Tables

Physical and photophysical properties and theoretical description of the ground state

Supplementary Table 1 | Thermal isomerization behavior of HTI 1. Thermal isomerization behavior of HTI **1** in MeCN-d_3/D_2O (8:2) at elevated temperature.

Isomer	$k_{(isom.\,1/isom.\,2)}$ / s^{-1} (at T / °C)	ΔG* (therm. isomer equilibration) /kcal mol^{-1}	Equilibration half-life of pure isomer at 27 °C	Thermo-dynamic isomer 1/isomer 2 equilibrium in the dark (at T / °C)	ΔΔG^0(isomer 1 / isomer 2) / kcal mol^{-1}	Slope m / s^{-1} (at T / °C)
**C**	$k_{(\mathbf{C/B})}$ = 1.46x10^{-4} (60 °C)	ΔG*(**C/B**) = 25.42	4.2 d	**C/B** = 13/87 (60 °C)	ΔΔG^0(**C/B**) = 1.26	5.97x10^{-4} (60 °C)
**D**	$k_{(\mathbf{D/A})}$ = 9.38x10^{-5} (27 °C)	ΔG*(**D/A**) = 23.10	125 min	**D/A** = 7/93 (27 °C)	ΔΔG^0(**D/A**) = 1.54	5.68x10^{-4} (27 °C)

Supplementary Table 2 | Thermal isomerization behavior of HTI 1. Thermal isomerization behavior of HTI **1** in DMSO-d_6 at elevated temperature.

Isomer	$k_{(isom.\,1/isom.\,2)}$ / s^{-1} (at T / °C)	ΔG* (therm. isomer equilibration) /kcal mol^{-1}	Equilibration half-life of pure isomer at 27 °C	Thermo-dynamic isomer 1/isomer 2 equilibrium in the dark (at T / °C)	ΔΔG^0(isomer 1 / isomer 2) / kcal mol^{-1}	Slope m / s^{-1} (at T / °C)
**A**	$k_{(\mathbf{A/D})}$ = 1.03x10^{-5} (60 °C)	ΔG*(**A/D**) = 27.17	80 d	**A/D** = 91/9 (60 °C)	ΔΔG^0(**A/D**) = -1.53	1.17x10^{-4} (60 °C)
**B**	$k_{(\mathbf{B/C})}$ = 8.50x10^{-6} (80 °C)	ΔG*(**B/C**) = 28.98	4.6 a	**B/C** = 85/15 (80 °C)	ΔΔG^0(**B/C**) = -1.21	5.48x10^{-5} (80 °C)
**C**	$k_{(\mathbf{C/B})}$ = 3.54x10^{-5} (80 °C)	ΔG*(**C/B**) = 27.98	0.86 a	**C/B** = 19/81 (80 °C)	ΔΔG^0(**C/B**) = 1.02	4.37x10^{-5} (80 °C)
**D**	$k_{(\mathbf{D/A})}$ = 1.01x10^{-4} (60 °C)	ΔG*(**D/A**) = 25.67	6.5 d	**D/A** = 11/89 (60 °C)	ΔΔG^0(**D/A**) = 1.38	1.13x10^{-4} (60 °C)

Supplementary Table 3 | Thermal isomerization behavior of HTI 1 in 12DCB-d_4 at 130 °C. The values were obtained by fitting the experimental obtained kinetic data to Supplementary Equation 6. A refinement factor of 0.98 was obtained. *High barriers can only be determined with large uncertainty as this reactions were only performed by a small amount of molecules. The given values are therefore only a lower limit for these barriers.

Reaction	ΔG* /kcal mol^{-1}
**A → B**	34*
**A → C**	34.8*
**A → D**	31.28
**B → A**	33.5*
**B → C**	32.5
**B → D**	33.8*
**C → A**	33.4*
**C → B**	31.5
**C → D**	32.7
**D→ A**	29.52
**D → B**	32.3*
**D → C**	32.45*

Supplementary Table 4 | Quantum yields of HTI 1. Quantum yields ϕ in % for the different photoconversions of isomers **A-1** and **B-1** after 520 nm illumination in different solvents at 25 °C. SBR = single-bond rotation, DBI = double-bond isomerization, HT = hula twist.

	*c*Hex	ODCB
**A to B SBR**	<0.1%[a]	<0.01%[a]
**A to C DBI**	16%[a]	0.10%[a]
**A to D HT**	1.1%[a]	<0.01%[a]
**B to A SBR**	<0.1%[a]	<0.01%[a]
**B to C HT**	<0.1%[a]	<0.01%[a]
**B to D DBI**	14%[a]	0.18%[a]

Supplementary Table 5 | Ground state energies of HTI 1. Summary of the calculated energies for important stationary points of the ground state energy profile of **1**.

Isomer	G_0 (Hartree)	ΔG (Hartree)	ΔG (kcal mol^{-1})
**A**	-1611.0717	0.000444	0.2786142
**B**	-1611.0722	0	0
**C**	-1611.0691	0.003038	1.90637377
**D**	-1611.0693	0.002875	1.80408972
**TS 1**	-1611.0113	0.060863	38.1921088
**TS 2**	-1611.0183	0.053814	33.7687946
**TS 3**	-1611.0161	0.056018	35.1518254
**TS 4**	-1611.0203	0.051834	32.5263258

Calculated ground state geometries - xyz coordinates

Supplementary Table 6 | Calculated ground state geometries of A-1 and B-1.

A-1				B-1			
S	-1.481773	-1.246402	0.2849	S	1.469092	-1.272251	-0.24029
O	-3.849107	1.790765	-0.52053	O	3.817627	1.829947	-0.600104
O	-1.143427	-1.416524	1.748121	O	1.159916	-1.694249	-1.659401
C	-3.227436	-1.63712	0.112206	C	3.223529	-1.606151	0.003942
C	-3.757436	-2.909618	0.258224	C	3.757592	-2.867157	0.21004
H	-3.124147	-3.76316	0.469258	H	3.122142	-3.739785	0.304317
C	-5.132985	-3.058958	0.11982	C	5.142118	-2.979512	0.297532
H	-5.576179	-4.041881	0.226391	H	5.588628	-3.952149	0.466364
C	-5.950121	-1.960719	-0.159298	C	5.962559	-1.857205	0.170561
H	-7.018455	-2.103646	-0.266998	H	7.037399	-1.970152	0.243537
C	-5.404691	-0.693076	-0.299144	C	5.412618	-0.600958	-0.046028
H	-6.022466	0.171006	-0.512927	H	6.033976	0.280873	-0.148283
C	-4.029808	-0.540938	-0.151164	C	4.029899	-0.487616	-0.12859
C	-3.296066	0.742278	-0.260439	C	3.280192	0.774563	-0.341112
C	-1.834454	0.513599	-0.063658	C	1.816881	0.517233	-0.193984
C	-0.764563	1.339678	-0.131923	C	0.761952	1.337814	0.005262
C	0.587838	0.701121	-0.084515	C	-0.593178	0.702983	0.028619
C	1.355051	0.636232	1.080589	C	-1.210954	0.290325	1.211673
C	2.64109	0.086819	1.031777	C	-2.50139	-0.248815	1.168277
C	3.162053	-0.416574	-0.181609	C	-3.194949	-0.349251	-0.058963
C	2.370938	-0.356736	-1.348623	C	-2.558781	0.068684	-1.246761
C	1.109233	0.206636	-1.275734	C	-1.271462	0.571798	-1.177958
C	-0.797423	2.865763	-0.340789	C	0.805688	2.871291	0.142762
C	0.610943	3.479334	-0.249133	C	-0.566019	3.432133	0.558046
H	1.054757	3.343606	0.738928	H	-1.344943	3.195513	-0.168921
H	1.291922	3.057482	-0.990719	H	-0.882866	3.053028	1.532649
H	0.529994	4.553783	-0.433504	H	-0.487066	4.519913	0.630825
C	-1.326818	3.14391	-1.762465	C	1.821319	3.321067	1.207926
H	-2.346468	2.786465	-1.890591	H	2.840383	3.03788	0.958577
H	-1.312582	4.222659	-1.943121	H	1.78555	4.41101	1.289669
H	-0.683677	2.669367	-2.509239	H	1.565703	2.907079	2.187047
C	-1.668633	3.553856	0.724694	C	1.161124	3.459474	-1.237583
H	-1.655348	4.633512	0.549612	H	1.151115	4.551657	-1.175263
H	-2.701245	3.216209	0.689902	H	2.149573	3.136232	-1.560867
H	-1.265474	3.373282	1.724644	H	0.423316	3.158549	-1.986846
H	0.506158	0.252095	-2.17807	H	-0.780457	0.874331	-2.097672
C	0.799673	1.131269	2.390844	C	-0.488526	0.396789	2.530407
H	1.311846	2.03856	2.728441	H	-0.25676	-0.595731	2.930248
H	-0.265066	1.347955	2.311385	H	0.455211	0.93273	2.431916
H	0.917211	0.378242	3.173148	H	-1.094326	0.914225	3.279132
N	4.449715	-0.94604	-0.237359	N	-4.478283	-0.888267	-0.107086

C	4.827715	-1.736304	-1.396525	C	-5.269319	-0.690051	-1.309206
H	4.395491	-2.749913	-1.343267	H	-5.669023	0.337075	-1.357066
H	5.914709	-1.846498	-1.381478	H	-6.124039	-1.368729	-1.25893
C	5.082978	-1.342739	1.006784	C	-5.236011	-0.964549	1.127868
H	4.647816	-2.27996	1.393277	H	-5.591222	0.032259	1.439867
H	6.137783	-1.535791	0.797979	H	-6.118348	-1.581001	0.941462
C	3.460303	0.022878	2.30384	C	-3.150212	-0.708038	2.457457
H	3.059265	-0.769544	2.948642	H	-3.432929	0.172178	3.048868
H	3.34809	0.953595	2.865746	H	-2.425007	-1.25684	3.063972
C	2.864524	-0.912869	-2.665496	C	-3.254574	-0.015227	-2.587108
H	2.410354	-1.896887	-2.832646	H	-3.611253	0.98252	-2.869386
H	2.52724	-0.271844	-3.484344	H	-2.535782	-0.316468	-3.353823
C	4.380388	-1.054344	-2.677595	C	-4.435783	-0.975293	-2.546532
H	4.708385	-1.635305	-3.542344	H	-5.050925	-0.870141	-3.442881
H	4.853694	-0.069192	-2.738781	H	-4.082976	-2.010591	-2.503884
C	4.936461	-0.238936	2.0364	C	-4.37905	-1.575964	2.219997
H	5.416358	0.665572	1.649756	H	-4.077281	-2.579789	1.905297
H	5.448124	-0.515639	2.960898	H	-4.957202	-1.677577	3.141105

Supplementary Table 7 | Calculated ground state geometries of C-1 and D-1.

C-1				D-1			
S	-3.523047	0.92962	0.007782	S	3.522394	0.934465	-0.152384
O	-0.712144	-1.707252	0.702463	O	0.773127	-1.413233	1.28036
O	-3.799199	1.451996	-1.384561	O	4.061788	1.599728	1.095855
C	-4.06003	-0.781009	0.051677	C	3.996908	-0.799157	-0.038381
C	-5.376898	-1.179728	-0.118145	C	5.262957	-1.278743	-0.328369
H	-6.165857	-0.460253	-0.301582	H	6.035518	-0.630713	-0.724808
C	-5.653051	-2.540017	-0.043208	C	5.511249	-2.628446	-0.095254
H	-6.671919	-2.884794	-0.173347	H	6.488992	-3.037262	-0.320313
C	-4.63721	-3.467373	0.203584	C	4.518796	-3.460869	0.424508
H	-4.879888	-4.521389	0.26331	H	4.736521	-4.508062	0.596035
C	-3.325398	-3.049448	0.370591	C	3.257366	-2.959871	0.719562
H	-2.523496	-3.75374	0.557385	H	2.475576	-3.592466	1.123141
C	-3.044697	-1.689828	0.28171	C	3.005532	-1.615273	0.479739
C	-1.701285	-1.076589	0.40684	C	1.715307	-0.916387	0.710293
C	-1.784091	0.403781	0.16289	C	1.788429	0.463254	0.122687
C	-0.726313	1.237507	0.062705	C	0.727805	1.206016	-0.253349
C	0.642285	0.633612	0.03534	C	-0.634576	0.606689	-0.119637
C	1.425802	0.502514	1.183534	C	-1.463983	0.861838	0.971579
C	2.725997	-0.002138	1.080581	C	-2.75775	0.329788	0.997978
C	3.245472	-0.399008	-0.172566	C	-3.235604	-0.445575	-0.082106
C	2.436732	-0.281052	-1.319457	C	-2.384605	-0.700885	-1.176075
C	1.158116	0.238625	-1.190822	C	-1.101526	-0.178093	-1.165698
C	-0.776778	2.76328	-0.154614	C	0.798726	2.588802	-0.941389
C	-0.933888	3.004784	-1.672616	C	-0.600124	3.211442	-1.092007
H	-0.078414	2.603955	-2.221966	H	-1.266036	2.585465	-1.688011
H	-1.849555	2.544119	-2.045281	H	-1.072875	3.385672	-0.122805
H	-0.975943	4.08239	-1.856893	H	-0.498118	4.176775	-1.594227
C	-1.920122	3.456836	0.607045	C	1.652574	3.589459	-0.141159
H	-1.964841	3.130379	1.649411	H	2.697711	3.299715	-0.047575
H	-1.739547	4.53445	0.599988	H	1.622578	4.559627	-0.644193
H	-2.89387	3.304274	0.14313	H	1.252904	3.721899	0.867636
C	0.536652	3.429096	0.297611	C	1.381114	2.393314	-2.356522
H	0.475609	4.496955	0.074157	H	1.370354	3.349345	-2.88707
H	0.69625	3.321356	1.372862	H	2.409462	2.033217	-2.323035
H	1.404388	3.022764	-0.222567	H	0.780708	1.681299	-2.929051
H	0.539741	0.318578	-2.079901	H	-0.448676	-0.385824	-2.008922
C	0.866988	0.855455	2.538627	C	-0.966756	1.683707	2.132935
H	1.419664	1.676898	3.005061	H	-0.902805	1.077889	3.04212
H	-0.180527	1.152312	2.476451	H	0.029069	2.085021	1.944465
H	0.927627	0.000805	3.218571	H	-1.634832	2.522371	2.349532
N	4.552464	-0.882277	-0.281797	N	-4.523893	-0.984905	-0.055699
C	4.922551	-1.590634	-1.495027	C	-5.086259	-1.475128	-1.301957
H	4.506476	-2.612877	-1.500992	H	-5.399123	-0.640848	-1.953269
H	6.011303	-1.684098	-1.504936	H	-5.982949	-2.049834	-1.057864

C	5.198368	-1.36872	0.923143	C	-5.483514	-0.397688	0.860334
H	4.780819	-2.341764	1.234731	H	-5.810753	0.597452	0.5127
H	6.255309	-1.526482	0.69559	H	-6.365548	-1.042009	0.879757
C	3.559968	-0.144642	2.336812	C	-3.64242	0.609678	2.19521
H	3.18085	-0.991639	2.923083	H	-3.958015	1.660476	2.167361
H	3.435285	0.73759	2.969874	H	-3.067593	0.494372	3.117923
C	2.924212	-0.726987	-2.680115	C	-2.846249	-1.515236	-2.364555
H	2.487137	-1.704864	-2.91558	H	-3.082307	-0.837324	-3.193565
H	2.563261	-0.033917	-3.44489	H	-2.029488	-2.154691	-2.710486
C	4.442381	-0.835339	-2.721983	C	-4.078523	-2.345899	-2.032348
H	4.769501	-1.350091	-3.628145	H	-4.526675	-2.753757	-2.941154
H	4.89473	0.161535	-2.723289	H	-3.807305	-3.187968	-1.387732
C	5.038746	-0.354862	2.039483	C	-4.87154	-0.288828	2.243917
H	5.497809	0.587102	1.723327	H	-4.59267	-1.292207	2.580433
H	5.563296	-0.69419	2.935574	H	-5.604006	0.10586	2.951691

Supplementary Table 8 | Calculated ground state geometries of TS-1 and TS-1.

TS-1				TS-2			
S	-2.071138	-0.762011	1.200955	S	2.80863	0.49377	-1.350652
O	-2.577098	1.361012	-2.027297	O	1.428482	-0.560621	2.134576
O	-2.504664	-0.293035	2.581199	O	3.549711	1.815616	-1.520805
C	-3.567249	-1.171359	0.285422	C	3.88923	-0.642218	-0.458371
C	-4.485038	-2.139431	0.647793	C	5.101963	-1.118929	-0.91785
H	-4.349084	-2.742272	1.53871	H	5.493015	-0.830251	-1.887062
C	-5.595233	-2.320833	-0.177681	C	5.810966	-1.99139	-0.090343
H	-6.334521	-3.071919	0.07526	H	6.76668	-2.383326	-0.418341
C	-5.756567	-1.548183	-1.32607	C	5.296317	-2.36499	1.14886
H	-6.621173	-1.709095	-1.960124	H	5.856595	-3.050859	1.77427
C	-4.821316	-0.57216	-1.665569	C	4.072094	-1.866346	1.592558
H	-4.934121	0.037327	-2.555167	H	3.659937	-2.147961	2.555149
C	-3.727494	-0.378837	-0.836992	C	3.375228	-0.986527	0.780441
C	-2.626255	0.626907	-1.019425	C	2.057218	-0.331417	1.080402
C	-1.720008	0.534999	0.046942	C	1.686345	0.47533	0.004488
C	-0.460734	1.323068	0.09077	C	0.437396	1.252237	-0.050998
C	0.70634	0.729483	-0.356779	C	-0.752909	0.673273	-0.438631
C	2.01438	1.338408	-0.617961	C	-0.936174	-0.767111	-0.697416
C	3.15615	0.613192	-0.440729	C	-2.159965	-1.343004	-0.477993
C	3.072296	-0.783815	-0.075526	C	-3.289852	-0.526944	-0.093185
C	1.815612	-1.467877	-0.249126	C	-3.205238	0.904911	-0.261575
C	0.707809	-0.718637	-0.431294	C	-1.982026	1.439917	-0.426342
C	-0.596463	2.726156	0.732765	C	0.549138	2.71561	0.469769
C	-1.160436	3.734487	-0.290438	C	-0.468173	3.011628	1.596781
H	-0.476004	3.944156	-1.112781	H	-1.496931	3.132206	1.263563
H	-2.086639	3.355844	-0.723608	H	-0.44618	2.207855	2.337729
H	-1.363673	4.67848	0.22394	H	-0.179585	3.939125	2.098102
C	-1.632172	2.63858	1.874608	C	1.93457	2.956573	1.101691
H	-1.341029	1.906993	2.630202	H	2.740011	2.777539	0.390045
H	-1.707643	3.620988	2.348513	H	1.984201	3.997255	1.436238
H	-2.614938	2.348341	1.506437	H	2.086395	2.31123	1.970279
C	0.692906	3.250075	1.391534	C	0.42349	3.701024	-0.71125
H	0.439646	4.102999	2.026174	H	0.523092	4.7249	-0.339183
H	1.140605	2.482858	2.029376	H	1.230009	3.522	-1.427252
H	1.444767	3.59669	0.691031	H	-0.523494	3.630041	-1.24752
H	-0.240693	-1.223855	-0.536193	H	-1.926271	2.511881	-0.529077
C	2.121269	2.662386	-1.323486	C	0.167993	-1.639515	-1.227143
H	2.481979	2.470995	-2.340054	H	-0.242035	-2.414162	-1.873999
H	1.164907	3.161822	-1.413852	H	0.88327	-1.06639	-1.806518
H	2.839491	3.337968	-0.854648	H	0.706158	-2.13776	-0.416182
N	4.149642	-1.44372	0.321632	N	-4.406563	-1.072117	0.363807
C	4.116391	-2.886047	0.594581	C	-5.586715	-0.260145	0.688099
H	3.853571	-3.040827	1.646522	H	-5.512529	0.073096	1.728918
H	5.125624	-3.266815	0.442642	H	-6.456719	-0.910134	0.605991

C	5.41288	-0.731652	0.53158	C	-4.480634	-2.510791	0.630762
H	5.364425	-0.187295	1.48056	H	-3.988494	-2.721088	1.586228
H	6.204343	-1.47451	0.609402	H	-5.531658	-2.776331	0.727163
C	4.515554	1.230029	-0.701064	C	-2.361166	-2.840086	-0.62419
H	4.686373	2.022717	0.036096	H	-1.763517	-3.352786	0.137378
H	4.516872	1.714252	-1.680509	H	-1.979375	-3.173673	-1.590451
C	1.748884	-2.97183	-0.144865	C	-4.440237	1.766363	-0.155818
H	1.352472	-3.245032	0.83933	H	-4.428332	2.287069	0.807981
H	1.049072	-3.357459	-0.88903	H	-4.416572	2.53237	-0.933617
C	3.128697	-3.587934	-0.320541	C	-5.701584	0.923164	-0.254973
H	3.113934	-4.651878	-0.080037	H	-6.582984	1.508615	0.009839
H	3.46519	-3.48444	-1.356537	H	-5.839475	0.554711	-1.27586
C	5.649828	0.216121	-0.629396	C	-3.818427	-3.263429	-0.503825
H	5.704356	-0.368757	-1.55194	H	-4.359272	-3.046552	-1.429517
H	6.606508	0.724352	-0.501933	H	-3.886714	-4.337218	-0.324374

Supplementary Table 9 | Calculated ground state geometries of TS-2 and TS-3.

TS-3				TS-4			
S	-2.860656	0.854389	-1.185133	S	-2.644409	0.446149	-1.451629
O	-1.482997	-1.110566	1.877855	O	-2.022594	-0.193953	2.336013
O	-2.376725	0.410957	-2.561239	O	-2.067042	-0.47743	-2.519793
C	-3.992838	-0.405352	-0.566815	C	-3.99653	-0.423435	-0.63664
C	-5.228461	-0.706086	-1.107407	C	-5.156161	-0.854231	-1.252529
H	-5.619195	-0.164208	-1.961437	H	-5.337006	-0.675963	-2.306576
C	-5.965793	-1.72995	-0.510409	C	-6.094381	-1.526498	-0.467984
H	-6.940407	-1.990086	-0.906683	H	-7.014383	-1.880499	-0.91832
C	-5.460295	-2.414461	0.592347	C	-5.858501	-1.741499	0.888168
H	-6.050111	-3.20182	1.047925	H	-6.602839	-2.258208	1.483453
C	-4.206361	-2.099401	1.114188	C	-4.677984	-1.302401	1.485336
H	-3.801045	-2.625951	1.970912	H	-4.48149	-1.463944	2.539335
C	-3.467807	-1.092398	0.514356	C	-3.737332	-0.649728	0.704684
C	-2.097697	-0.619924	0.910552	C	-2.404219	-0.123705	1.148203
C	-1.699445	0.421979	0.063712	C	-1.729007	0.417623	0.055287
C	-0.479651	1.247866	0.191228	C	-0.460878	1.156817	0.101124
C	0.715199	0.707207	-0.251211	C	0.744279	0.547319	-0.202928
C	1.998521	1.369911	-0.477146	C	0.96788	-0.909554	-0.202355
C	3.164177	0.671285	-0.356381	C	2.243653	-1.400411	-0.055006
C	3.128452	-0.742704	-0.050956	C	3.395105	-0.532798	-0.146382
C	1.886797	-1.454376	-0.2053	C	3.189264	0.868916	-0.425996
C	0.753096	-0.735273	-0.355065	C	1.93223	1.346537	-0.395805
C	-0.66055	2.575269	0.970604	C	-0.580144	2.65439	0.509385
C	-1.265871	3.681095	0.082589	C	-0.488482	3.546191	-0.750713
H	-0.599311	3.997709	-0.720478	H	-0.669256	4.58594	-0.463875
H	-2.203065	3.348764	-0.366572	H	0.473001	3.504768	-1.261677
H	-1.479829	4.555818	0.703229	H	-1.259859	3.258194	-1.470101
C	-1.674911	2.301206	2.108529	C	-1.961597	2.939244	1.133102
H	-1.367814	1.443721	2.711566	H	-2.136177	2.315406	2.011036
H	-1.724688	3.182439	2.752938	H	-1.991267	3.987966	1.440792
H	-2.673509	2.101285	1.720518	H	-2.774369	2.77359	0.424861
C	0.618158	3.071445	1.66958	C	0.447851	3.054595	1.589784
H	0.345816	3.854128	2.381849	H	0.227336	4.068208	1.934567
H	1.093614	2.261287	2.228823	H	0.361625	2.380731	2.44658
H	1.351862	3.504082	0.997668	H	1.484092	3.041551	1.258898
H	-0.191229	-1.252998	-0.43842	H	1.813672	2.399918	-0.587823
C	2.048864	2.740467	-1.095289	C	-0.150524	-1.909421	-0.320229
H	2.400735	2.634198	-2.127156	H	-0.672059	-2.032732	0.633102
H	1.072585	3.20875	-1.139639	H	-0.876154	-1.586532	-1.06183
H	2.748063	3.409034	-0.588858	H	0.237345	-2.879005	-0.625551
N	4.238	-1.386929	0.286175	N	4.634107	-1.002228	-0.034467
C	4.25485	-2.83655	0.517434	C	5.830748	-0.164176	-0.207547
H	4.052652	-3.027632	1.577001	H	6.191826	-0.29525	-1.233778
H	5.264327	-3.185723	0.302301	H	6.593688	-0.559003	0.46367

C	5.487367	-0.650071	0.490957	C	4.920768	-2.429526	0.139619
H	5.458488	-0.157222	1.468712	H	5.058794	-2.880085	-0.849678
H	6.300758	-1.373152	0.503123	H	5.866241	-2.508355	0.675814
C	4.497709	1.34436	-0.607857	C	2.485571	-2.877198	0.184919
H	4.663878	2.095175	0.173061	H	2.483331	-3.419915	-0.768015
H	4.460488	1.88824	-1.5548	H	1.679465	-3.291002	0.789659
C	1.857957	-2.959834	-0.11121	C	4.366185	1.755137	-0.725359
H	1.517953	-3.248975	0.889378	H	4.606547	1.693291	-1.793053
H	1.128782	-3.354546	-0.821641	H	4.110405	2.794043	-0.510856
C	3.240078	-3.542546	-0.363634	C	5.566036	1.296953	0.087499
H	3.260148	-4.610952	-0.143782	H	6.45834	1.872942	-0.161916
H	3.523662	-3.412228	-1.412412	H	5.370473	1.428906	1.155764
C	5.661541	0.362756	-0.624661	C	3.80688	-3.111585	0.897289
H	5.702867	-0.171206	-1.578297	H	3.763999	-2.712731	1.915324
H	6.60727	0.891695	-0.499278	H	4.019307	-4.179288	0.968208

Crystal structure analysis

Supplementary Table 10 | Crystal structure analysis of A-1 and B-1.

Compound	**A-1**<br>CCDC 1910546	**B-1**<br>CCDC 1910547
net formula	$C_{26}H_{29}NO_2S$	$C_{27}H_{31}Cl_2NO_2S$
M_r/g mol^{-1}	419.56	504.49
crystal size/mm	0.080 × 0.070 × 0.030	0.070 × 0.030 × 0.030
T/K	110.(2)	110.(2)
radiation	MoKα	MoKα
diffractometer	'Bruker D8 Venture TXS'	'Bruker D8 Venture TXS'
crystal system	triclinic	monoclinic
space group	'P -1'	'P 1 21/n 1'
a/Å	7.0410(5)	15.1748(8)
b/Å	8.5158(6)	10.8807(6)
c/Å	18.4408(15)	15.4174(7)
α/°	81.582(3)	90
β/°	87.739(3)	92.771(2)
γ/°	76.464(2)	90
V/Å^3	1063.40(14)	2542.6(2)
Z	2	4
calc. density/g cm^{-3}	1.310	1.318
μ/mm^{-1}	0.176	0.362
absorption correction	Multi-Scan	Multi-Scan
transmission factor range	0.93–0.99	0.91–0.99
refls. measured	10942	21971
R_{int}	0.0421	0.0404
mean σ(I)/I	0.0540	0.0338
θ range	2.577–26.370	2.304–25.349
observed refls.	3515	3620
x, y (weighting scheme)	0.0364, 0.5689	0.0375, 2.2661
hydrogen refinement	constr	constr
Flack parameter	-	-
refls in refinement	4318	4511
parameters	275	340
restraints	0	30
$R(F_{obs})$	0.0416	0.0412
$R_w(F^2)$	0.1014	0.1054
S	1.017	1.068
shift/error$_{max}$	0.001	0.001
max electron density/e Å^{-3}	0.278	0.276
min electron density/e Å^{-3}	−0.330	−0.262

Supplementary Table 11 | Crystal structure analysis of C-1 and D-1.

Compound	**C-1**<br>CCDC 1910548	**D-1**<br>CCDC 1910549
net formula	$C_{26}H_{29}NO_2S$	$C_{26}H_{29}NO_2S$
M_r/g mol^{-1}	419.56	419.56
crystal size/mm	0.100 × 0.090 × 0.020	0.090 × 0.060 × 0.020
T/K	109.(2)	111.(2)
radiation	MoKα	MoKα
diffractometer	'Bruker D8 Venture TXS'	'Bruker D8 Venture TXS'
crystal system	monoclinic	triclinic
space group	'P 1 21/n 1'	'P -1'
a/Å	14.6022(7)	7.0642(9)
b/Å	7.0310(3)	12.2255(14)
c/Å	21.6607(10)	13.2154(16)
α/°	90	78.599(4)
β/°	103.123(2)	87.959(4)
γ/°	90	75.015(4)
V/Å^3	2165.79(17)	1080.6(2)
Z	4	2
calc. density/g cm^{-3}	1.287	1.289
μ/mm^{-1}	0.172	0.173
absorption correction	Multi-Scan	Multi-Scan
transmission factor range	0.92–1.00	0.89–1.00
refls. measured	31376	11750
R_{int}	0.0485	0.0406
mean σ(I)/I	0.0321	0.0551
θ range	3.070–26.372	2.985–27.101
observed refls.	3564	3692
x, y (weighting scheme)	0.0320, 4.2363	0.0473, 0.6988
hydrogen refinement	constr	constr
Flack parameter	-	-
refls in refinement	4415	4735
parameters	299	275
restraints	36	0
$R(F_{obs})$	0.0747	0.0484
$R_w(F^2)$	0.1649	0.1259
S	1.145	1.045
shift/error$_{max}$	0.001	0.001
max electron density/e Å^{-3}	0.341	0.486
min electron density/e Å^{-3}	−0.397	−0.320

Supplementary References

1. A. Gerwien, T. Reinhardt, P. Mayer, H. Dube, Synthesis of Double-Bond Substituted Hemithioindigo Photoswitches. *Org. Lett.* **1**, 232-235 (2018).

2. A. Gerwien, P. Mayer, H. Dube, Photon-Only Molecular Motor with Reverse Temperature-Dependent Efficiency. *J. Am. Chem. Soc.* **140**, 16442-16445 (2018).

3. U. Megerle, R. Lechner, B. König, E. Riedle, Laboratory apparatus for the accurate, facile and rapid determination of visible light photoreaction quantum yields. *Photochem. Photobiol. Sci.* **9**, 1400-1406 (2010).

4. Gaussian 16, Revision A.03, M. J. Frisch, G. W. Trucks, H. B. Schlegel, G. E. Scuseria, M. A. Robb, J. R. Cheeseman, G. Scalmani, V. Barone, G. A. Petersson, H. Nakatsuji, X. Li, M. Caricato, A. V. Marenich, J. Bloino, B. G. Janesko, R. Gomperts, B. Mennucci, H. P. Hratchian, J. V. Ortiz, A. F. Izmaylov, J. L. Sonnenberg, D. Williams-Young, F. Ding, F. Lipparini, F. Egidi, J. Goings, B. Peng, A. Petrone, T. Henderson, D. Ranasinghe, V. G. Zakrzewski, J. Gao, N. Rega, G. Zheng, W. Liang, M. Hada, M. Ehara, K. Toyota, R. Fukuda, J. Hasegawa, M. Ishida, T. Nakajima, Y. Honda, O. Kitao, H. Nakai, T. Vreven, K. Throssell, J. A. Montgomery, Jr., J. E. Peralta, F. Ogliaro, M. J. Bearpark, J. J. Heyd, E. N. Brothers, K. N. Kudin, V. N. Staroverov, T. A. Keith, R. Kobayashi, J. Normand, K. Raghavachari, A. P. Rendell, J. C. Burant, S. S. Iyengar, J. Tomasi, M. Cossi, J. M. Millam, M. Klene, C. Adamo, R. Cammi, J. W. Ochterski, R. L. Martin, K. Morokuma, O. Farkas, J. B. Foresman, and D. J. Fox, Gaussian, Inc., Wallingford CT, (2016).

5. Bruker, *SAINT.* Bruker AXS Inc., Madison, Wisconsin, USA, (2012).

6. G. M. Sheldrick, *SADABS.* University of Göttingen, Germany, (1996).

7. G. M. Sheldrick, *SHELXT* – Integrated space-group and crystal-structure determination. *Acta Cryst.* A**71**, 3-8 (2015).
