# Supplementary material for: Green light powered molecular state motor enabling eight-shaped unidirectional rotation
Source: Nat Commun. 2019 Oct 1;10:4449. doi: 10.1038/s41467-019-12463-4 (PMC6773862; doi:10.1038/s41467-019-12463-4)
Supplement: Supplementary file 2 — Description of Additional Supplementary Files [file 41467_2019_12463_MOESM2_ESM.pdf]

Description of Additional Supplementary Files

File Name: Supplementary Movie 1
Description: First of four possibilities for the trajectory of the unidirectional 8-shaped motion of molecular motor 1.

File Name: Supplementary Movie 2
Description: Second of four possibilities for the trajectory of the unidirectional 8-shaped motion of molecular motor 1.

File Name: Supplementary Movie 3
Description: Third of four possibilities for the trajectory of the unidirectional 8-shaped motion of molecular motor 1.

File Name: Supplementary Movie 4
Description: Fourth of four possibilities for the trajectory of the unidirectional 8-shaped motion of molecular motor 1.
